# GLOBal river SALiniTy and associated ions (GlobSalt)

**DOI:** 10.1038/s41598-025-96222-0

**Published:** 2025-05-28

**Authors:** Alvaro Javier Moyano-Salcedo, Theresa Piana, Julie Crabot, Ben J. Kefford, Elisabeth Berger, Shelley E. Arnott, Josefin Thorslund, Michel Meybeck, Sujay S. Kaushal, Ralf B. Schäfer, Miguel Cañedo-Argüelles

**Affiliations:** 1 FEHM-Lab (Freshwater Ecology, Hydrology and Management), Departament de Biologia Evolutiva, Ecologia i Ciències Ambientals, Facultat de Biologia, Universitat de Barcelona, Barcelona, Spain; 2 Geohazards and Civil Engineering Research Group, Department of Civil Engineering, Saint Thomas Villavicencio University, C/22 No 1a, Villavicencio, 500003 Colombia; 3 Centre for Applied Water Science, Institute for Applied Ecology, University of Canberra, Canberra, ACT Australia; 4 RPTU Kaiserslautern-Landau, iES - Institute for Environmental Sciences, Landau, Germany; 5 Department of Biology, Queen’s University, Kingston, ON K7L 3N6 Canada; 6 Department of Physical Geography, Bolin Centre for Climate Research, Stockholm University, Stockholm, Sweden; 7 Department of Physical Geography, Utrecht University, Utrecht, The Netherlands; 8 UMR 7619 METIS, Sorbonne Université-CNRS-EPHE, Paris, France; 9 Department of Geology & Earth System Science Interdisciplinary Center, University of Maryland, College Park, MD USA; 10 FEHM-Lab (Freshwater Ecology, Hydrology and Management), Institute of Environmental Assessment and Water Research (IDAEA), CSIC, Carrer de Jordi Girona, SHE-218-26, 08034 Barcelona, Spain

**Keywords:** Freshwater salinization, Global salinity data, Salinization hotspots, Electrical conductivity, Major ions, Freshwater ecology, Ecological modelling, Freshwater ecology

## Abstract

Freshwater salinization (FS) is a threat to freshwater ecosystems, but its impact remains relatively poorly understood compared to other stressors (e.g. nutrient pollution), with some regions (e.g. Asia, Africa) remaining poorly explored. To assess how pervasive this issue is globally and identify salinization hotspots, we compiled global data on river salinity and associated ions. We retrieved information from different sources, harmonized it and merged it with HydroATLAS watersheds. Our global data set (GlobSalt) features 13 parameters, including electrical conductivity (EC), major ions, and nutrients. GlobSalt contains approximately fifteen million records on a monthly scale for river stations from 1980 to 2023 from all continents except Antarctica. The global median EC was 509 ± 205 μS cm^−1^, with 60% of rivers falling in the range of 50 to 500 μS cm^−1^, which is within the salinity niche of most freshwater organisms. We found a large spatial variability in EC, with some regions such as the Mediterranean, the Midwest of the US, arid regions of Argentina and Chile and Southwestern Australia having high mean salinity values. Temporally, EC was fairly stable. GlobSalt represents a critical resource for improving our understanding of FS dynamics, identifying regions at high risk and informing management strategies.

## Introduction

Freshwater salinization (FS) is a growing water quality challenge that poses a risk to the health of aquatic ecosystems. FS has the potential not only to degrade water use across sectors^1,2^ (e.g., agricultural irrigation, drinking water supply), but also to cause strong negative changes in both the structure (e.g., by shifting community composition) and functioning (e.g., e.g., biomass production) of river ecosystems ^3–8^. FS can result from a combination of natural processes such as chemical weathering (e.g., dissolution of ions), fluvial salt transport (e.g., sediment transport), soil erosion (e.g., runoff carrying saline minerals from the soil into rivers), atmospheric deposition (e.g., salt deposition by rainfall or gravitational settling), and atmospheric transport of salts (e.g., wind transport of sea salt particles in aerosols or dusts)^9^. Other natural sources are saltwater intrusion, biological (e.g., natural release of salts through decomposition and the salt transportation of biological vectors such as migratory animals), geological (e.g., salt release due to tectonic movements such as geologic faults) and climate-related processes (e.g., El Niño and La Niña events)^9^. Furthermore, human activities have strongly increased FS on a global scale, modifying and intensifying natural salt fluxes. For instance, chemical weathering can be induced by factors such as fertilizers, acid rain, or mining^10–12^. This is concurrent with other activities that increase fluvial salt transport like road de-icing, as well as domestic and industrial discharges^13–17^. Moreover, the intensification of agricultural irrigation can lead to soil erosion, which, in turn, may result in the dissolution of chemicals (e.g., sodium, chloride)^18–20^ that can alter the biological cycle of salt^9,21,22^. Also, the anthropogenic water cycle can directly affect atmospheric conditions (resulting in increased salt deposition), surface water (e.g., causing fluctuations in river flow due to water extraction), and groundwater dynamics (e.g., influenced by groundwater pumping), thereby altering salinity concentrations^9,23,24^. These effects extend to geologic uplift and resource extraction activities such as fracking^9,25,26^. Finally, FS may be driven by climatic conditions, exacerbating its impact in arid and semi-arid areas, and affecting rivers globally^27–31^.

Water quality monitoring programs should consider FS not only based on electrical conductivity (EC) as a salinity indicator but also on the concentrations of individual ions (Na^+^, K^+^, Mg^2+^, Ca^2+^, Cl^−^, SO_4_^2−^, SiO_3_^2−^) and their interactions with other chemicals. For example, river salinization often co-occurs with nutrients and pesticides from agriculture, the food industry, and energy systems^22,32,33^. Agriculture is the primary source of increased levels of NaCl, as well as NO₃⁻, PO_4_^3−^, K⁺, Ca^2⁺^, and Mg^2⁺^^34^. However, the concentration of these ions in many rivers and streams remains unknown^16,20^. Similar challenges arise from other activities, such as wastewater discharge in urban areas and industrial production (e.g., ethanol and agro-food industries), which generate by-products that release high concentrations of both nutrients and salts, significantly amplifying FS^33,35–37^. In the ethanol industry, sugarcane vinasse emerges as a substantial and continuous by-product with elevated nutrient levels (e.g., pure vinasse has a total N: 639 mg L^−1^, total P: 150 mg L^−1^) and salinity (e.g., sodium: 60 mg L^−1^, conductivity: 8420 μS cm^−1^)^32,33^. Moreover, many river basins may experience the combined effects of agricultural and wastewater discharges^38–40^. The resulting “chemical cocktails” can have profound effects on aquatic communities^18^ by disrupting ecological dynamics and potentially favoring the dominance of certain species while threatening the survival of other^22^. Therefore, monitoring chemical mixtures associated with FS is important for the conservation and sustainable management of aquatic ecosystems.

So far, although EC is routinely measured as a proxy to anthropogenic FS activities, FS has been often neglected in water quality monitoring programs^41,42^. and there are no legally enforced FS standards to protect aquatic life^2^. While there have been considerable efforts to compile and harmonize water quality information, including salinity parameters, at the global scale^34,43–46^ a significant part of this information remains scattered. For example, the global occurrence of major elements (excluding EC measurements) in rivers has been compiled^34^. Also, the GLORICH^43^ database updates and provides data on major ions and other physicochemical parameters of water. However, spatial and temporal gaps persist in both datasets. Similarly, the global dataset of surface water and groundwater salinity measurements from 1980 to 2019^46^ provides a valuable contribution to understanding the distribution and evolution of salinity over time, but includes only EC data, providing only a narrow understanding of FS dynamics. Within this context, our database presents two significant improvements: (1) We provide new data for some regions of the world that were poorly represented, mainly in Asia and Africa. (2) We provide data on specific ions and on other water quality parameters that are important to understand and manage the effects of FS^18,47^. Data related to FS (e.g., EC, water ion composition) and other pollutants (e.g., nutrients) often vary in reported parameters, units, and spatiotemporal resolution. This hampers the comparison of information across scales. Thus, the compilation, harmonization, and access to these data are crucial for the development of models and analyses that can elucidate the dynamics of FS and propose management measures at local, regional, and global scales.

We provide a global, standardized database with a set of key water quality parameters related to salinity, including nutrients, covering the period from 1980 to 2023. We collected and compiled observational data with a primary focus on EC, as it is the most widely monitored parameter for salinity worldwide. However, whenever more detailed information on other major ions parameters was available, this was considered. Data were collected from a variety of sources, including local, regional, and global water quality databases, governmental organizations, river basin management commissions, water development boards, and individual research projects. This is the most comprehensive dataset on FS to date, with the potential to be used by researchers, policymakers, conservation organizations and other stakeholders to anticipate, mitigate and restore the impacts of FS on aquatic ecosystems around the world.

## Methods

A total of 60 data sources were used to compile the GLOBal river SALiniTy and associated ions database (GlobSalt). The GlobSalt database features 13 parameters, including major ions, EC, ammonium, nitrate, and phosphate (Table 1). All datasets were publicly available online, and some have been explored and used in previous studies^43,44,46^. The number of available parameters differed strongly between countries and the parameters with the highest global coverage and temporal representativeness were selected.

**Fig. 1:**
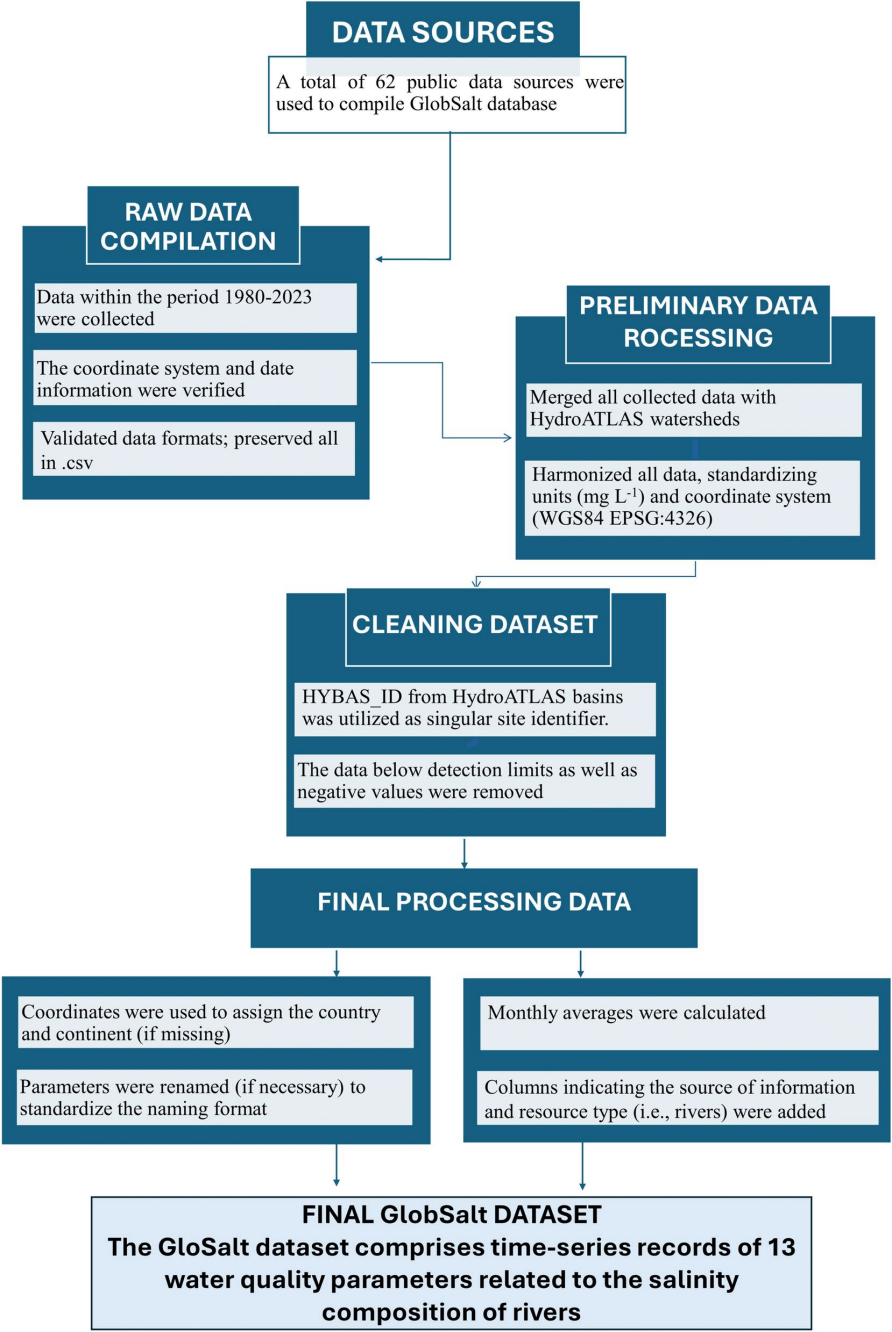
Methodology overview flowchart. The figure illustrates the data compilation, processing, and harmonizing steps of the GlobSalt dataset.

Data compilation was divided into three parts: (1) raw data was downloaded and converted into a common format and the corresponding metadata was harmonized; (2); subsequently, the data were merged based on parameters, ensuring a unified structure for all information, including aspects like identifiers and temporal scales; (3) finally, we cleaned and organized the final dataset (Fig. 1).

### Compilation and source data preparation

All raw data compilation was downloaded or queried from a total of 62 online sources listed in Table S1 including existing global datasets. Where available, direct links to these data sources have been included in Table S1 to facilitate access and reproducibility. For datasets obtained through restricted access or institutional requests, no direct link is available. In some cases (e.g. India), direct requests were made to the environmental authorities who authorized the use of the data. Preliminary processing data from each source revealed ambiguities in parameter names, codes, units, or chemical forms, as well as in the complementary information for each watershed, including the geographic coordinate systems used. This was expected when dealing with water quality data from multiple sources^48,49^.

### Parameter selection and harmonization

The selection of parameters followed those of previous studies and datasets that address the global salinization of rivers, such as Global Occurrence of Major Elements in Rivers^34^, GLORICH^43^, and more recently a global dataset of surface water and groundwater salinity measurements from 1980 to 2019^46^. The data harmonization and standardization approach used in this study was based on the methodologies of all these previous datasets and was also adapted from the Global River Water Quality Archive (GRQA)^44^ to ensure consistency and comparability across datasets. All these studies stand out as global datasets that strive to compile salinity variables with the least number of discrepancies in source data (e.g., parameters with a single matching code and consistent units).

The nomenclature for the included parameters varied across the data sources, with various abbreviations, names, and chemical forms. To address this issue, we undertook a comprehensive renaming of all variables (Table 1).

### Unit conversion

The units for each parameter were harmonized to ensure consistency, following a methodology adapted from the Global River Water Quality Archive (GRQA)^44^. Conversions were made where necessary, and all units except pH and EC (measured in μS cm^−1^ 25 °C) were standardized to milligrams per liter (mg L^−1^), which was the most prevalent unit in source data. A consistent review approach was also adopted for all parameters across continents (e.g., distinguishing between dissolved and total concentration). For GLORICH, which reported major ion parameters in micromoles per liter (µmol L^−1^), conversions to mg L^−1^ were performed. However, no conversion was required for most of the sources (e.g., WQP), as its units matched those intended for the GlobSalt dataset (i.e., mg L^−1^). A detailed list of all unit conversion procedures can be found in Table S2, where the conversion was determined using the Eq. (1):

$${x}_{2}= \frac{{x}_{1} * M{x}_{2}}{n * M{x}_{1}}$$

Here,** x**_**1**_ represents the observation value before conversion; ** x**_**2**_ represents the observation value after conversion; **Mx**_**1**_ is the molar mass corresponding to the compound (e.g. NO3 = 62.005 g mol^−1^), and **Mx**_**2**_ is the molar mass corresponding to the elemental parameter (e.g. N = 14.007 g mol⁻^1^); n is the magnitude difference between the source and converted units (e.g. µmol L⁻^1^ to mg L⁻^1^, 1000).

### Site ID duplication and watershed characteristics

The information was intersected with the basins from HydroAtlas^50^, and subsequently, a single basin identifier (i.e., HYBAS_ID) was used to link all information. We selected HydroATLAS for its global coverage and standardized watershed and river categorization. Additionally, it provides a comprehensive dataset with a wide range of hydro-environmental attributes at high spatial resolution, derived from publicly available data and categorized into six groups: hydrology, physiography, climate, land cover and land use, geology and soils, and anthropogenic impacts. The integration of these data not only unifies all EC and major ion information from GlobSalt into a single, organized structure, but also summarizes it at the sub-catchment scale and enhances its utility for future analyses. Also, to distinguish each basin from each country, the original identifiers from each source and database were retained. Duplicate identifiers were then systematically identified and eliminated. The number of watershed characteristics for a site varied strongly, where some provided details such as state, watershed, city, and river name that were lacking for other sites. This complicated the use of a common watershed characteristic (e.g., watershed name) across sites. We created a general summary for each watershed in our final dataset, including an identifier (station_ID), country, continent, water type, and data source. However, all details regarding watershed descriptions were preserved in a separate file linked to the station_ID.

### Coordinate conversion

Verification of the coordinate systems used by each data source revealed some variability between them. For example, information from countries such as the US, Germany, and France sometimes used specific datums such as the North American Datum of 1983 (NAD83), Gauss-Krueger, and Lambert 93 (EPSG:2154). In these cases, the spTransform() function in R, a component of the sp (spatial) package, was used to convert geographic coordinates to the World Geodetic System 1984 (WGS84). All information was then standardized to the chosen coordinate system (WGS84 EPSG:4326) and integrated into the final dataset (Table 1).

### Information filtering

Preliminary data cleaning included removing observations with negative or missing values, or those from unreliable sources. In addition, observations marked as below (<) or above (>) the detection limit in the source data were excluded from the GlobSalt when the information sources included data on the detection limits. This approach aligns with established methodologies from the Global River Water Quality Archive (GRQA)^44^, which similarly applies rigorous quality control measures to ensure data reliability. Four source datasets (WQP, GEMStat, GLORICH, and Waterbase) had this type of quality assessment included in the metadata. In all cases, a review was performed to identify the column representing the final validated measurement parameter result for each source, and this value was used in GlobSalt.

### Time and date processing

The source datasets used different date formats, all of which were converted to a common format (%Y-%m-%d) using the “as.Date()” function in R. Observations with incorrectly formatted or implausible dates were removed. In addition, the hour was omitted because it was a rare parameter across sources. Some sources provided daily measurements, and some provided monthly and annual averages. For date manipulation, including extraction of month and day, the “lubridate” package in R was used with functions such as “year()”, “month()” and “day()”. The final time unit for presenting data was the monthly average of all available data per month. This was done to harmonize the data into a common global unit, to reduce the volume of data, and to include as much data as possible (e.g. from areas where daily data were lacking).

### Outlier processing, time series availability, and continuity

The total number of observations varies considerably between sources. The North American WQP stands out with a large data set of 9.649.775 records, much higher than other sources. This variability in data volume is also accompanied by differences in the temporal coverage of the measurements (Table S1). For example, the European Waterbase covers several decades, while others, such as RCHB-T (Bogotá Water Quality Network, Colombia), are limited to specific years (Table S1). The specific number of observations per country and continent vary for each parameter but, as expected, EC and pH show greater continuity and number of records.

Data distribution checking involved a visual examination and a plausibility check. First, the bean plot was used to examine the distribution of the log-transformed data of each parameter (Figs. S1 to S26). The log transformation was used to normalize the data distribution by effectively mitigating the effects of extreme values or outliers, which are common in environmental data sets. Subsequently, adherence to a distribution within the lognormal distribution family was evaluated using histograms. For example, the bean plot of EC (Fig. S1) showed that, although most of the data ranged around the median, values that could be potential outliers were visible and should be considered cautiously when using our data in analyses. The histogram of EC (Fig. S2) showed a strong deviation from a theoretical log normal distribution. However, all measurements from the GlobSalt database were retained, as both EC concentration data and major ions can vary widely between geographic locations due to natural and anthropogenic processes, seasonality, and different measurement methodologies^44^.

### GlobSalt dataset records

The GlobSalt dataset consists of time series observations of water quality parameters that reflect the salinity composition of rivers. These parameters (Table 1) are consolidated into a single CSV file (“GlobSalt_dataset.csv”) along with location data and simplified identifiers. Additional information, such as catchment characteristics, is stored in a separate file 'GlobSalt_catchment_characteristics.csv’ (files available for download in the Data Availability section). Each dataset is accompanied by metadata files (tables and images) that provide comprehensive details on the spatial and temporal characteristics of the respective time series. Figures S1 to S26 illustrate the distribution of records for each parameter. Also, a data catalogue ('GlobSalt data catalogue’ section in the supplementary information) contains maps showing the spatio-temporal coverage and violin plots describing the concentration by continent, the temporal dispersion of the data, as well as correlations of all salinity parameters (Figs. C1 to C41).

**Table 1:** GlobSalt attributes, structure, and parameters statics. Variable names and descriptions, including reported units, of the GlobSalt database.

Attribute	Description	Unit	Number of total records by attribute	Number of sites	Median value per water parameter
HYBAS_ID	Sampling point basin ID HydroAtlas	#	14,274,097	–	–
x	Longitudinal coordinate of sample location	Decimal Degrees	14,274,097	–	–
y	Latitudinal coordinate of sample location	Decimal Degrees	14,274,097	–	–
year	Year of the sample in record	yyy	14,274,097	–	–
month	Month of the sample in record	mm	14,274,097	–	–
Station_ID	Sampling river point ID associated with each basin	#	14,274,097	–	–
Country	Geographic location	Name	14,274,097	–	–
Continent	Geographic location	Name	14,274,097	–	–
Source	Data source information of the dataset (See Table A1 for source names)	Name	14,274,097	–	–
Water_type	Water resource type sampled	River	14,274,097	–	–
Quality_data_flag	Flagged records. 0 for accurate, 1 for potentially erroneous data	#	14,274,097	–	–
Conductivity	Measurement of electrical EC in the surface water	μS cm^−1 @25C^	10,746,278	45,435	509
pH	Measurement of acidity or alkalinity in the surface water	–	11,054,612	44,159	7.7
Sodium, dissolved	Concentration of dissolved Sodium ions in the surface water	mg L^−1^	5,526,891	19,086	30
Potassium, dissolved	Concentration of dissolved Potassium ions in the surface water	mg L^−1^	5,206,061	17,613	4
Magnesium, dissolved	Concentration of dissolved Magnesium ions in the surface water	mg L^−1^	5,953,892	21,308	16
Calcium, dissolved	Concentration of dissolved Calcium ions in the surface water	mg L^−1^	6,086,082	22,505	46
Chloride, dissolved	Concentration of dissolved Chloride ions in the surface water	mg L^−1^	5,856,822	22,133	33
Sulphate, dissolved	Concentration of dissolved Sulphate ions in the surface water	mg L^−1^	5,494,879	31,167	57
Silicate, dissolved	Concentration of dissolved Silicate ions in the surface water	mg L^−1^	427,933	11,080	9
Nitrate	Concentration of Nitrate ions in the surface water	mg L^−1^	1,799,644	60,651	0.37
Ammonium	Concentration of Ammonium ions in the surface water	mg L^−1^	1,363,067	30,577	0.40
Phosphate	Concentration of Phosphate ions in the surface water	mg L^−1^	961,175	13,728	0.16
Total organic carbon (TOC)	Measurement of the total amount of organic carbon in the surface water	mg L^−1^	825,456	30,145	4.2

**Fig. 2:**
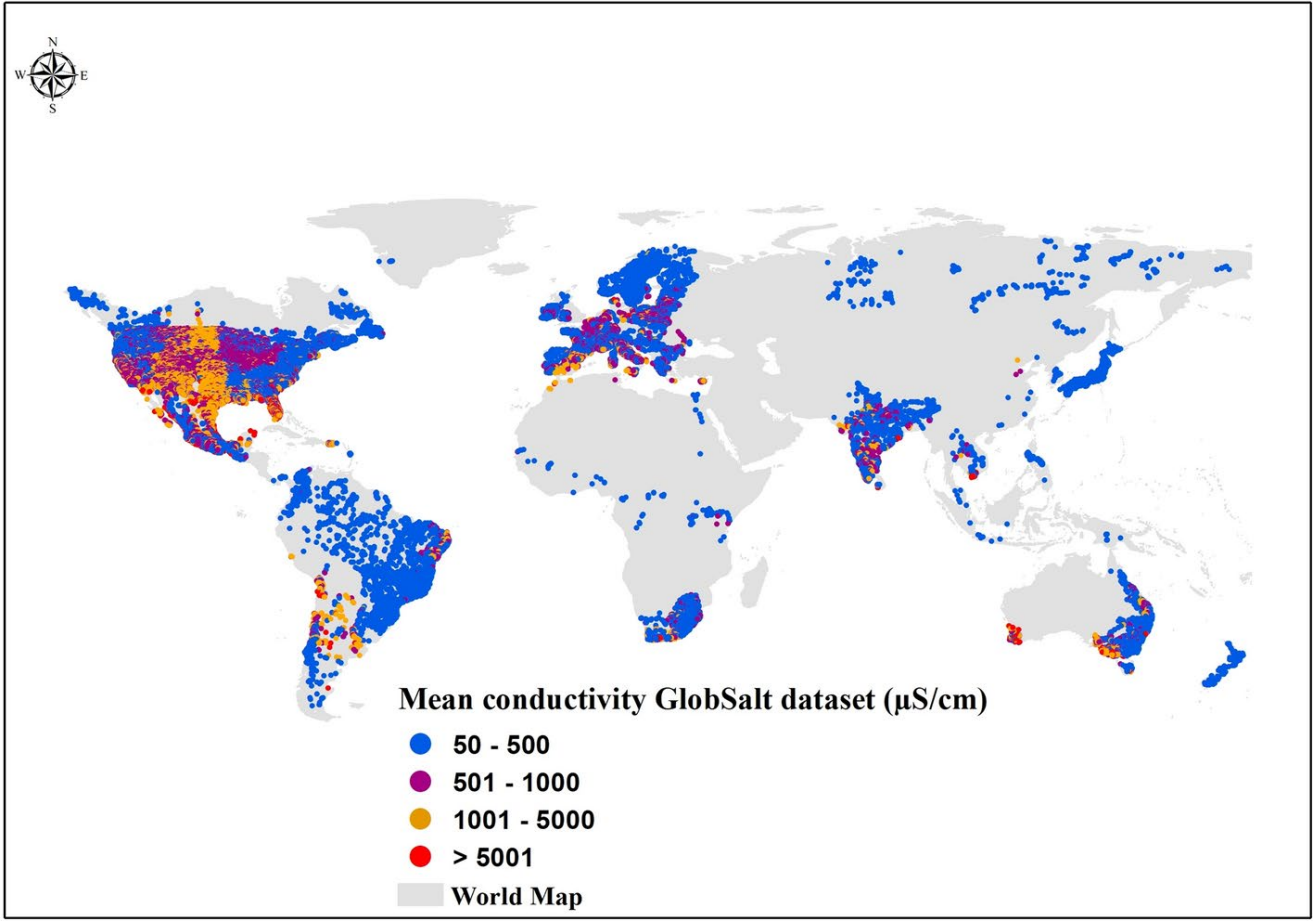
Global distribution of measured mean electrical conductivity (EC) and station density. The global map in the panel shows the measured mean EC values per river in each country, based on observations included in the GlobSalt database, over the entire data period (1980–2023). Blue, purple, orange, and red dots represent low, moderate, moderately high, and high EC, respectively.

**Fig. 3:**
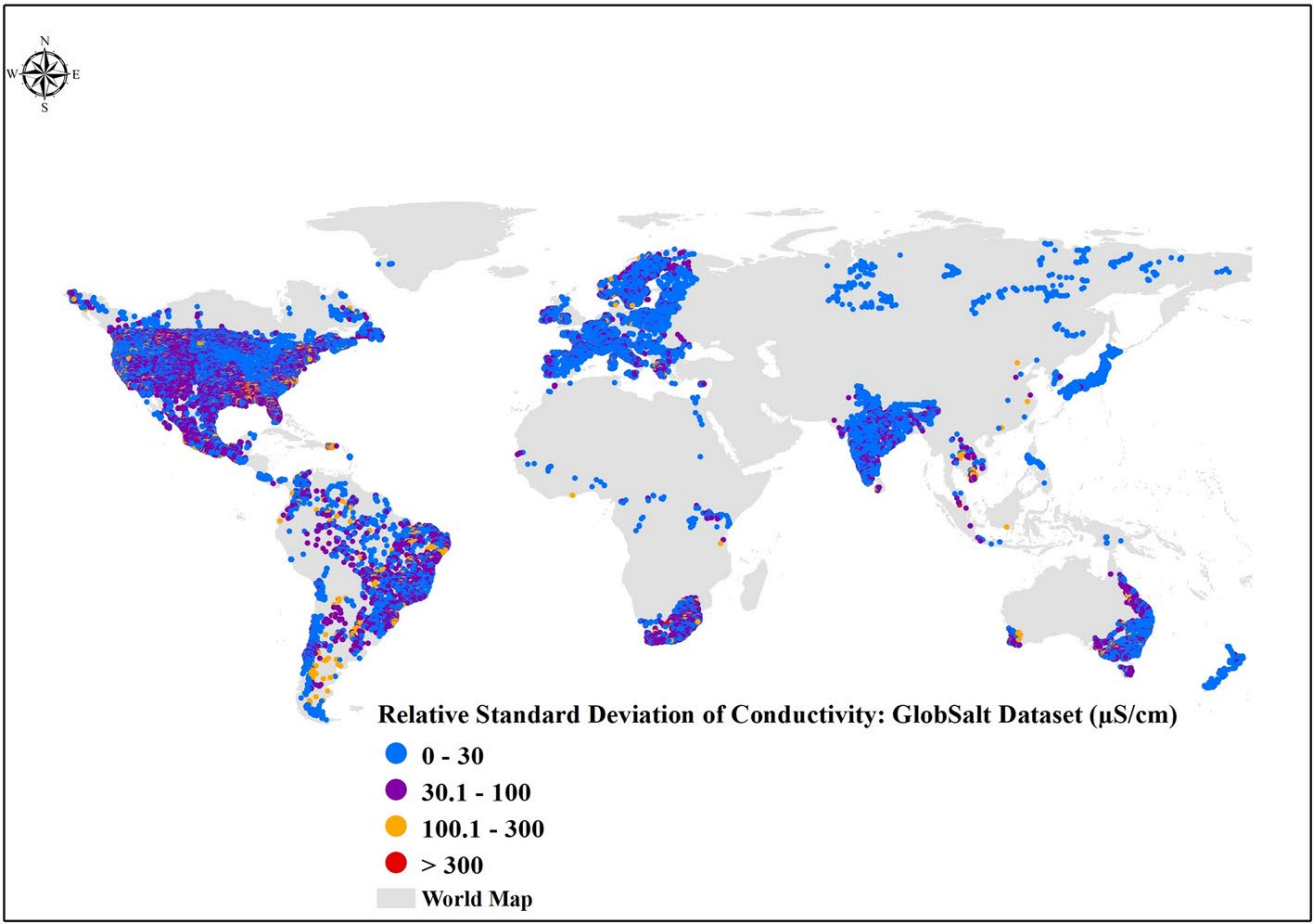
Global Distribution of Relative Standard Deviation (RSD) of Electrical Conductivity (EC) and Station Density. The global map illustrates the RSD of EC values per river in each country, calculated as (SD/Mean) × 100%, from observations in the GlobSalt database (1980–2023). Blue, purple, orange, and red dots represent low, moderate, moderately high, and high RSD of EC, indicating variability around the mean conductivity.

**Fig. 4:**
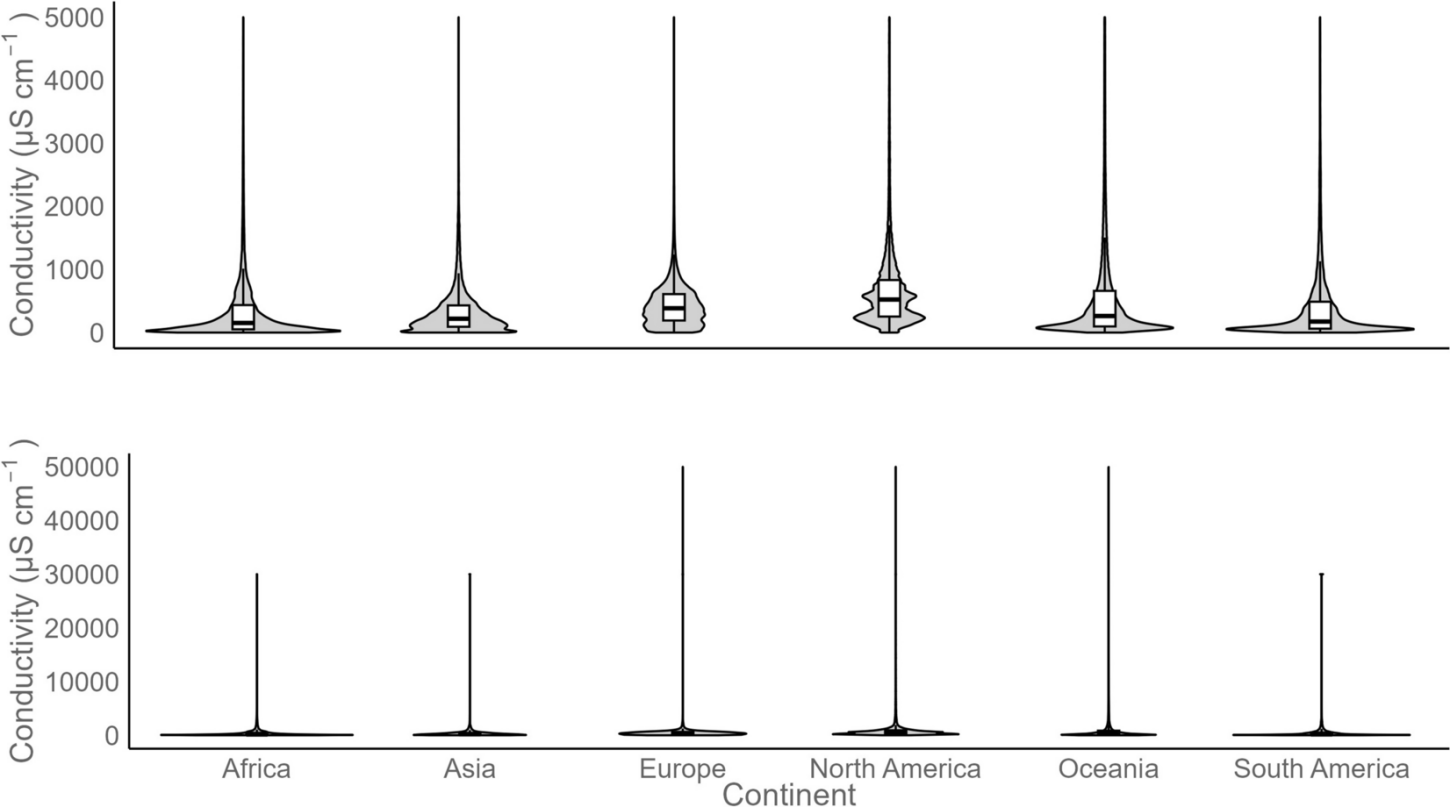
Violin plot of electrical conductivity (EC) by continent. Top: Global Violin Plot of Electrical Conductivity (EC) by Continent with Zoom to 5000 μS cm^−1^. Bottom: Violin Plot of Electrical Conductivity (EC) across the entire scale by Continent. EC observations are included in the GlobSalt database, over the entire data period (1980–2023). Each gray violin plot represents the available data set according to the number (n) of observations per continent (Africa n = 473,083, Asia n = 112,049, Europe n = 958,793, North America n = 8,728,636, Oceania n = 325,057, South America n = 110,550).

The spatial distribution and ranges of EC by continent, compiled on a global scale in the GlobSalt dataset (as a proxy for salinity), are shown in Figs. 2 and 3. Across all continents, the median EC did not exceed 800 μS cm^−1^ (Fig. 4). The global median EC was 509 ± 205 μS cm^−1^ (Table 1), with 60% of samples ranging from 50 to 500 μS cm^−1^, which is within the salinity niche of most freshwater organisms^51–53^. However, the computed median EC did not consider factors such as country size and spatiotemporal data availability by region and thus does not directly reflect FS. Note that the spatial and temporal distribution of samples within GlobSalt, and thereby the median, was strongly biased to North America and somewhat by Europe, where the largest data sets originated (Fig. 4). To capture this, a detailed breakdown of EC by country is provided in Table 2, along with its relation to the geographic area (km^2^) and the number of available monitoring data and sites in each case. Table 2 shows strong variability in the median EC by country and its relationship with area, available data, and monitoring sites. The USA stands out as the country with fewer discrepancies in the relationship between data availability and factors such as country size (Nº data/ km^2^ = 0.86), followed by Germany (Nº data/ km^2^ = 0.78).

**Table 2:** Distribution of mean conductivity (EC) data relative to area by country in GlobSalt.

Country	Number of sites	Number of data	Area (km^2^)	Nº data/ km^2^	Median Conductivity (EC)
Argentina	203	5529	2,780,400	0.0020	190
Australia	2160	284,661	7,741,220	0.0368	380
Austria	16	1474	83,871	0.0176	370
Bangladesh	5	311	148,460	0.0021	150
Belarus	1	60	207,600	0.0003	424
Belgium	63	5361	30,689	0.1747	822
Bolivia	11	118	1,098,581	0.0001	100
Bosnia and Herzegovina	14	1002	51,197	0.0196	400
Brazil	1961	37,754	8,515,770	0.0044	55
Bulgaria	74	3849	110,879	0.0347	414
Burundi	5	5	27,830	0.0002	37
Cambodia	12	960	181,035	0.0053	12
Cameroon	7	904	475,440	0.0019	18
Canada	2774	158,603	9,984,670	0.0159	223
Central African Republic	1	12	622,984	0.0000	40
Chile	557	19,014	756,102	0.0251	387
China	10	890	9,596,960	0.0001	258
Colombia	175	8917	1,138,910	0.0078	141
Croatia	51	4057	56,594	0.0717	388
Cyprus	37	1480	9251	0.1600	737
Czech Republic	2	489	78,867	0.0062	522
Democratic Republic of the Congo	1	12	342,000	0.0000	42
Denmark	11	18	42,930	0.0004	425
Ecuador	11	408	283,561	0.0014	52
Egypt	7	102	1,001,450	0.0001	285
Estonia	263	4659	45,227	0.1030	445
Finland	106	5400	338,145	0.0160	13
France	6642	370,873	643,801	0.5761	326
Germany	4048	281,889	357,022	0.7895	565
Ghana	4	15	238,533	0.0001	119
Greece	360	2008	131,957	0.0152	346
Greenland	14	14	2,166,086	0.0000	16
Guatemala	83	2566	108,889	0.0236	205
Hungary	16	2844	93,028	0.0306	386
India	961	79,421	3,287,263	0.0242	240
Indonesia	11	478	1,904,569	0.0003	158
Ireland	190	25,535	70,273	0.3634	240
Italy	2654	29,074	301,340	0.0965	488
Japan	17	5258	377,915	0.0139	118
Kenya	43	259	580,367	0.0004	160
Laos	14	1622	236,800	0.0068	118
Latvia	15	648	64,589	0.0100	493
Lesotho	1	95	30,355	0.0031	521
Lithuania	463	9150	65,300	0.1401	521
Luxembourg	2	58	2586	0.0224	797
Malaysia	7	476	329,847	0.0014	55
Mali	13	159	1,240,192	0.0001	44
Mexico	2125	71,386	1,964,375	0.0363	589
Moldova	6	597	33,846	0.0176	540
Morocco	8	560	446,550	0.0013	1445
Netherlands	5	261	41,543	0.0063	489
New Zealand	113	40,396	268,838	0.1503	90
Nigeria	1	5	1,267,000	0.0000	110
South Korea	32	2070	120,538	0.0172	151
Norway	182	15,584	323,802	0.0481	30
Pakistan	4	801	796,095	0.0010	300
Panama	57	4399	75,420	0.0583	112
Papua New Guinea	1	1	462,840	0.0000	150
Peru	14	174	1,285,216	0.0001	588
Philippines	1	1	300,000	0.0000	227
Poland	3293	43,144	312,685	0.1380	479
Portugal	87	3886	92,090	0.0422	479
Romania	27	4699	238,391	0.0197	391
Russia	3	180	17,098,242	0.0000	312
Rwanda	20	20	26,338	0.0008	211
Senegal	10	40	196,722	0.0002	151
Serbia	83	7288	77,474	0.0941	411
Slovakia	9	1287	49,037	0.0262	423
Slovenia	3	556	20,273	0.0274	314
South Africa	3404	470,581	1,219,090	0.3860	156
Spain	1440	120,272	505,370	0.2379	670
Sri Lanka	34	2086	65,610	0.0318	70
Sudan	2	188	1,861,484	0.0001	250
Sweden	948	26,583	450,295	0.0590	10
Switzerland	126	11,118	41,290	0.2693	425
Tanzania	6	120	947,300	0.0001	142
Thailand	22	2400	513,120	0.0047	182
Tunisia	1	12	163,610	0.0001	706
Uganda	2	5	241,038	0.0000	112
UK	898	23,020	243,610	0.0945	198
Ukraine	3	130	603,550	0.0002	289
Uruguay	8	365	176,215	0.0021	65
USA	4437	8,529,874	9,833,517	0.8674	553
Venezuela	4	65	912,050	0.0001	33
Viet Nam	54	3633	331,212	0.0110	192

**Fig. 5:**
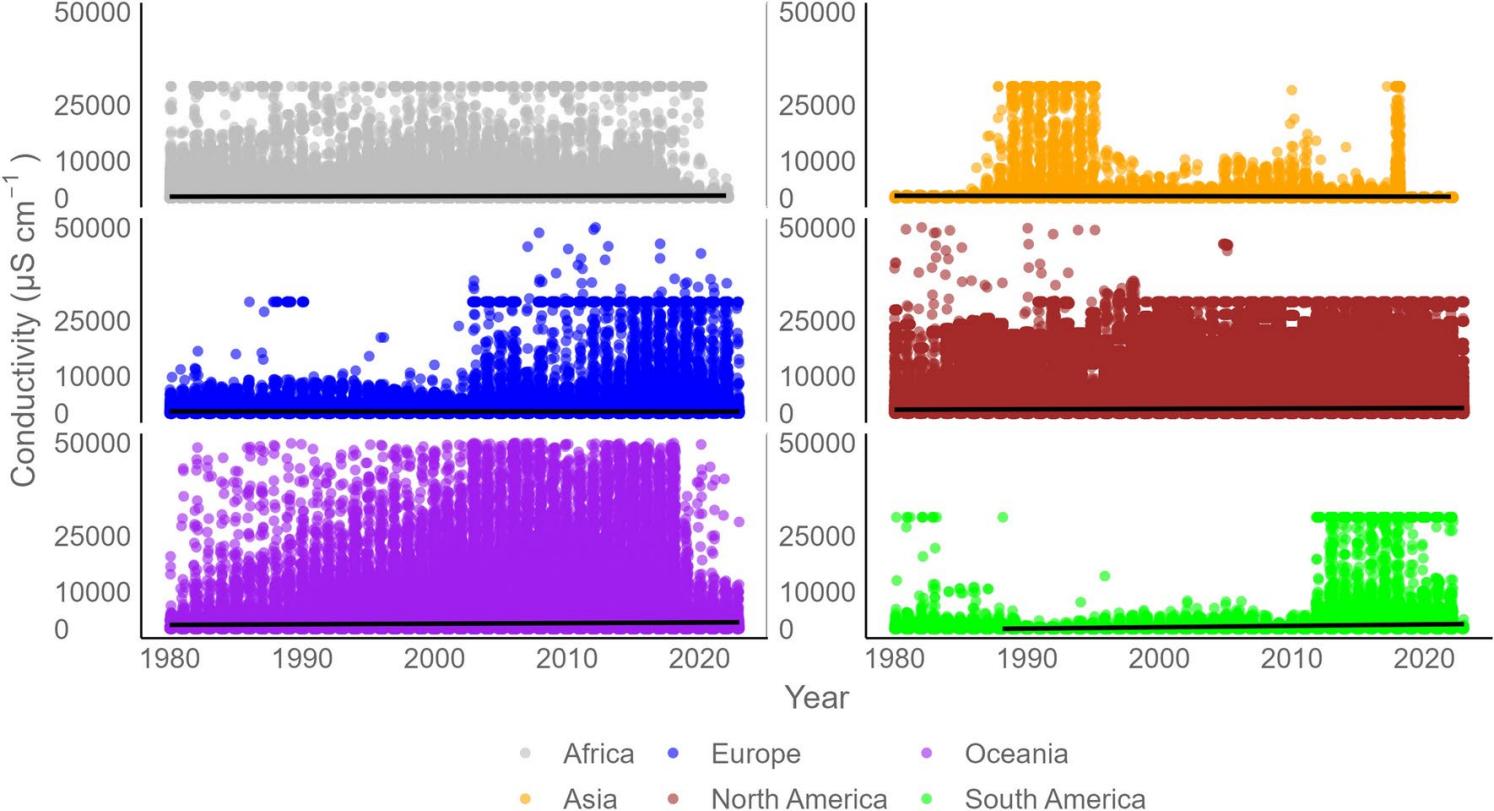
Global temporal dispersion plot of electrical conductivity (EC) by continent. EC observations are included in the GlobSalt database over the entire data period (1980–2023). Each color (gray, yellow, blue, brown, purple, and green) represents the number (n) of observations per continent (Africa n = 473,083, Asia n = 112,049, Europe n = 958,793, North America n = 8,728,636, Oceania n = 325,057, South America n = 110,550).

Despite the bias in available information on FS, a large spatial variability in mean EC was found when plotted by river station. Regions such as the Mediterranean^54^, the Midwest USA^55^, arid regions of Argentina and Chile, and southwestern Australia^52^ had high mean salinity values (Fig. 2) as well as greater relative variation (Fig. 3). Temporally, EC remained relatively stable, with no evidence of strong increasing or decreasing trends from 1980 to 2023 (Fig. 5). Across continents and over time, we found an upper EC limit ranging between 25,000 and 30,000 μS cm^−1^ (Fig. 5). We subset the data by filtering for “ceiling values” (i.e., EC data in the range of 25,000 to 30,000 μS cm^−1^) as well as extreme values (i.e., EC above 30,000 μS cm^−1^) to visually explore whether this could be due to measurement error or artifact (Figs. C37–C40). Since the location of these sites corresponded to saline or coastal regions and the correlation between EC and salt ions was strong in the high salinity subset data (Figs. C38–C39), we suggest that these values are correct and should not be removed from the dataset.

**Fig. 6:**
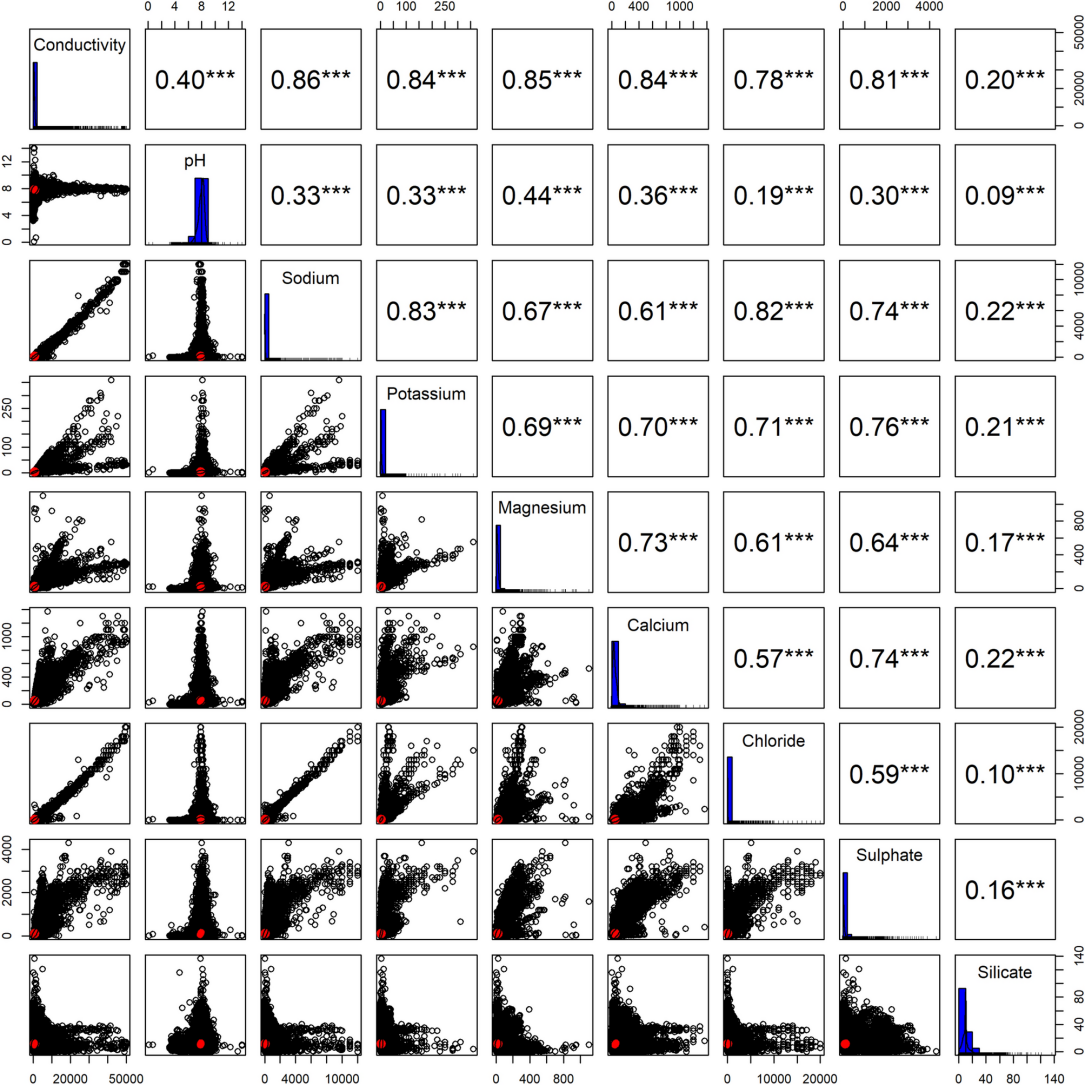
Correlation plot of electrical conductivity (EC) and ion concentrations in the GlobSalt dataset, including inter-ion correlations (ions unit, mg L^−1^). Red dots indicate the strength of the correlation between variables.

The correlations between EC and the major ions using the data from GlobSalt are shown in Fig. C41. This correlation shows that sodium and chloride were the elements most strongly correlated with EC (r^2^ > 0.5), but also reveals some discrepancies mainly related to the association of very low EC values with very high ion levels (e.g., sodium). To validate the EC measurements in relation to the sodium, potassium, and calcium concentrations (as a proxy for the relationship between EC and ions), the ratio (e.g., sodium/EC) between them was used. First, the theoretical EC was calculated based on the chemical composition of the measured ion concentrations (i.e., sodium, potassium, calcium) using the equation and methodology described in a previous study^56^. Then, the theoretical ratio (e.g., sodium/theoretical EC) was computed, yielding a mean and median of 0.43 and 0.44 for sodium, 0.35 and 0.36 for potassium, and 0.29 and 0.30 for calcium. These results are higher than with previously reported ion and EC ratio values (e.g., ratio of 0.3 for Sodium/EC)^57^. Also, it is important to note that this method has limitations related to the theoretical predictability of extreme values (both low and high), as highlighted in previous studies^58–60^. However, despite these limitations, the methodology remains useful for EC and ion data falling outside of these extreme intervals (e.g., where EC ranges from 100 to 1000 μS cm^−1^)^58,59^, albeit with a cautionary note on interpretation. After computing the theoretical ratio, we calculated the empirical ratio between each ion and EC measured. In our study, the mean of these theoretical ratios (e.g., sodium/theoretical EC) was used as a reference point to establish a baseline for the empirical ratio (e.g., sodium/measured EC). Thus, when the empirical ratio exceeded the mean of these theoretical ratios (sodium: 0.43, potassium: 0.35, and calcium: 0.29), the corresponding data points were flagged in GlobSalt as potentially erroneous (0 represents accurate data, and 1, potentially erroneous data). Figure 6 shows the pairwise correlations between EC and the major ions present in GlobSalt after filtering out potentially erroneous data. The final distribution of the filtered GlobSalt data resulted in approximately 65% of the data set falling within the used ratios (i.e., considered as accurate information), while the remaining 35% were flagged as ‘errors’. Also, with this approach, it was possible to obtain a stronger correlation of EC with sodium and chloride (r^2^ > 0.8), which remained the ions most correlated with EC.

## Supplementary Information

Supplementary Information.

## Data Availability

The datasets generated and analyzed during this study are available in the Zenodo repository at https://zenodo.org/records/15020440. The following files are included: (1) GlobSalt_data.CSV: Contains processed observation data for 13 water quality parameters, along with associated metadata (observation site information, etc.). (2) GloSalt_catchment_characteristics file: Includes processed observation data along with the catchment characteristics metadata found in GlobSalt_data.CSV. (3) GlobSalt_Script.R: An R script providing a structured workflow for data processing, cleaning, exploratory analysis, and visualization of the GlobSalt dataset.. These datasets are publicly available for non-commercial use and are intended to support interpretation, replication, and further development of the results reported in this article.

## Code availability

The data for this study was mainly processed in R (version 3.6.0), but with cross-checking and corrections of spatial coordinates conducted using ArcGIS.
