# Supplementary material for: GLOBal river SALiniTy and associated ions (GlobSalt)
Source: Sci Rep. 2025 May 28;15:18701. doi: 10.1038/s41598-025-96222-0 (PMC12120065; doi:10.1038/s41598-025-96222-0)
Supplement: Supplementary file 1 — Supplementary Information. [file 41598_2025_96222_MOESM1_ESM.pdf]

GLOBal river SALiniTy and associated ions (GlobSalt)

Alvaro Javier Moyano Salcedo*[1,2,9], Theresa Piana[4], Julie Crabot[9], Ben J. Kefford[3], Elisabeth Berger[4], Shelley E. Arnott[5], Josefin Thorslund[6], Michel Meybeck[7], Sujay S. Kaushal[8], Ralf B. Schäfer[4], Miguel Cañedo-Argüelles Iglesias*[9]

1 FEHM-Lab (Freshwater Ecology, Hydrology and Management), Departament de Biologia Evolutiva, Ecologia i Ciències Ambientals, Facultat de Biologia, Universitat de Barcelona, Barcelona, Spain.

2 Geohazards and Civil Engineering Research Group, Department of Civil Engineering, Saint Thomas Villavicencio University, C/22 No 1a, 500003, Villavicencio, Colombia.

3 Centre for Applied Water Science, Institute for Applied Ecology, University of Canberra, ACT, Australia

4 RPTU Kaiserslautern-Landau, iES - Institute for Environmental Sciences, Landau, Germany

5 Department of Biology, Queen's University, Kingston, Ontario, Canada K7L 3N6

6 Department of Physical Geography and the Bolin Centre for Climate Research, Stockholm University, Stockholm, Sweden. Department of Physical Geography, Utrecht University, Utrecht, The Netherlands.

7 UMR 7619 METIS, Sorbonne Université-CNRS-EPHE, Paris, France

8 Department of Geology & Earth System Science Interdisciplinary Center, University of Maryland, College Park, MD, USA

9 FEHM-Lab (Freshwater Ecology, Hydrology and Management), SHE-2, Institute of Environmental Assessment and Water Research (IDAEA), CSIC, Carrer de Jordi Girona, 18-26, 08034, Barcelona, Spain.

*Corresponding authors: Alvaro Javier Moyano Salcedo, alvaromoyano@ub.edu

Miguel Cañedo-Argüelles Iglesias, mcahse@cid.csic.es

Table S1. Description of the sources used to compile the GlobSalt dataset.

Country/Continent /Global	Source	Description	URL	Number of river stations by source	Start Year	End Year
África	Ministère de l'Environnement, de l'Eau et de l'Assainissement - Direction Nationale de l'Hydraulique du Mali	Water quality in African rivers, government institute	https://dnhmali.org/?situation-hebdomadaire-de-la-qualite-de-l-eau	14	1987	2015
	Ministry of Agriculture - General Directorate for Water Resources	Water quality in African rivers, government institute	http://wsdb.mwe.go.ug/#	2	1980	1982
	Ministry of Environment, Water and Natural Resources - Central Water Testing Laboratory	Water quality in African rivers, government institute	https://wra.go.ke/water-quality-pollution-control/	33	1980	2015
	Ministry of Health - National Chemical Laboratories	Water quality in African rivers, government institute	https://www.sanas.co.za/pages/index.aspx?page=Annual-Reports	2	1980	1992
	Ministry of Water & Environment - Directorate of Water Resources Management	Water quality in African rivers, government institute	https://www.mwe.go.ug/library/directorate-water-resource-management	4	1980	1980
	Ministry of Water and Livestock - Water Laboratories Unit	Water quality in African rivers, government institute	https://bit.ly/4imWfka	4	1980	2012

Country/Continent /Global	Source	Description	URL	Number of river stations by source	Start Year	End Year
	Ministry of Water Resources and Irrigation - National Water Research Center	Water quality in African rivers, government institute	https://www.amatolawater.co.za/water-data/water-quality-reports	7	1980	1980
	Water Research Institute	Water quality in African rivers, government institute	https://www.waterresearchobservatory.org/data-and-resources/water-quality	11	1991	2012
Argentina	Instituto Nacional de Agua - Centro de Tecnología del Uso del Agua	Water quality in Argentine rivers, government institute	https://www.argentina.gob.ar/laboratorio-experimental-de-calidad-de-aguas-0	12	1980	2013
	Ministerio de Obras Públicas - Subsecretario de Planificación y Gestión Operativa de Proyectos Hídricos	Water quality in Argentine rivers, government institute	https://snih.hidricosargentina.gob.ar/Filtros.aspx	132	1980	2023
Asia	Ministry of Environment & Natural Resources - Central Environment Authority	Water quality in Asian rivers, government institute	https://bit.ly/4hwuc0j	12	1980	2015
	Ministry of Environmental Protection of the People's Republic of China - China National Environmental Monitoring Center	Water quality in Asian rivers, government institute	https://english.mee.gov.cn/Resources/Reports/	10	1980	1997

Country/Continent /Global	Source	Description	URL	Number of river stations by source	Start Year	End Year
	Ministry of Public Works - Research Institute for Water Resources Development	Water quality in Asian rivers, government institute	https://www.resdc.cn/	10	1980	1994
	National Institute for Environmental Studies - Center for Global Environment Research	Water quality in Asian rivers, government institute	https://www.resdc.cn/Default.aspx	17	1980	2022
	National Institute of Environmental Research - Environmental Diagnostics Research Department	Water quality in Asian rivers, government institute	https://www.mee.gov.cn/404/index.shtml	3	1982	2022
	University of Engineering and Technology - Institute of Environmental Engineering and Research	Water quality in Asian rivers, government institute	https://oec.njtech.edu.cn/overseasen/info/1065/1505.htm	4	1979	2003
	Mekong River Commission - Mekong Secretariat	Water quality in Asian rivers, government institute	https://www.mrcmekong.org/water-quality-monitoring/	96	1985	2009
Australia	QLD AU Gov	Water quality in Australian rivers, government institute	https://water-monitoring.information.qld.gov.au/	12	1993	2013
	Waterconnect	Water Connect data portal - Australia	https://water.data.sa.gov.au/Data/Map/Parameter/NoParameter/Lo	6	1980	2018

Country/Continent /Global	Source	Description	URL	Number of river stations by source	Start Year	End Year
			cation/Type/Interval/Latest			
	Waternws	Water Connect data portal - New South Wales government, Australia	https://realtimedata.waternsw.com.au/	413	1900	2023
	Watervic	Water Connect data portal - Victoria government, Australia	https://data.water.vic.gov.au/	259	1980	2023
	AU Gov	Water quality in Australian rivers, government institute	https://riverdata.mdba.gov.au/list-view	1198	1980	2018
	Department of the Environment and Heritage - Water Policy Section	Water quality in Australian rivers, government institute	https://www2.environment.nsw.gov.au/topics/water/water-quality/monitoring-and-reporting	8	1980	1987
Bangladesh	Department of Environment	Water quality in Bangladesh rivers, government institute	https://bit.ly/4hwFMZq	5	1980	1995
Brazil	Brazilian Institute of Environment, Renewable and Natural Resources	Water quality in Brazilian rivers, government institute	https://www.gov.br/ana/en/monitoring	81	1980	2013
Canada	CA Gov	Water quality in Canadian rivers	https://bit.ly/3XMIyTm	111	2000	2023

Country/Continent /Global	Source	Description	URL	Number of river stations by source	Start Year	End Year
	Ontario Gov	Water quality in Canadian rivers	https://bit.ly/3DukNbZ	423	2000	2016
Chile	General Directorate of Water	Water quality in Chilean rivers, government institute	https://dga.mop.gob.cl/servicios-de-informacion/	737	1980	2018
Colombia	Instituto de Hidrología, Meteorología y Estudios Ambientales	Water quality in Colombian rivers, government institute	https://ideam.gov.co/agua	140	2005	2013
	Instituto Nacional de Salud - Laboratorio Salud Ambiental	Water quality in Colombian rivers, government institute	https://www.minsalud.gov.co/sites/rid/lists/bibliotecadigital/ride/vs/pp/sa/inca-2015_reducido.pdf	2	1981	1988
	Red de Calidad Hídrica de Bogotá Tradicional (RCHB-T)	Water quality in Colombian rivers, Bogotá, government institute	https://oab.ambientebogota.gov.co/red-de-calidad-hidrica-de-bogota/	30	2006	2023
Ecuador	Ministerio de Desarrollo Urbano y Vivienda - Subsecretaría de Agua Potable, Saneamiento y Residuos Sólidos	Water quality in Ecuadorian rivers, government institute	https://www.ambiente.gob.ec/	3	1979	1986

Country/Continent /Global	Source	Description	URL	Number of river stations by source	Start Year	End Year
Europe	Waterbase	European Water Quality. European Environment Agency	https://www.eea.europa.eu/en/datahub/datahubitem-view/fbf3717c-cd7b-4785-933a-d0cf510542e1	13765	1969	2021
French	Office National de l¿Eau Potable - Laboratoire de la Qualité des Eaux	Water quality in French rivers, government institute	http://www.onep.ma/controle-qualite.htm	4	1987	2012
	SIEAdour-Garonne	Water quality in French rivers, government institute	https://adour-garonne.eaufrance.fr/	2968	1980	2023
Germany	Daten- und Kartendienst der LUBW	Water quality data, state of Baden-Württemberg Germany	https://udo.lubw.baden-wuerttemberg.de/public/	179	2000	2022
	Bavaria (BY): Bayrisches Landesamt für Umwel (LfU)	Water quality data, state of Bavaria Germany	https://www.gkd.bayern.de/de/downloadcenter/wizard	1180	1980	2023
	Berlin (BE): Senatsverwaltung für Mobilität, Verkehr, Klimaschutz und Umwelt (Berlin)	Water quality data, state of Berlin Germany	https://www.berlin.de/sen/uvk/en/	64	1954	2023

Country/Continent /Global	Source	Description	URL	Number of river stations by source	Start Year	End Year
	Bremen (HB): Freie Hansestadt Bremen	Water quality data, state of Bremen Germany	https://www.elwasweb.nrw.de/elwas-web/data/ow/guete/t38Messstelle.xhtml;jsessionid=26114FF70F4502E69C03D388469F12C3?marea=2&mtheme=3&lindex=0&nested=true	13	2001	2020
	Hamburg (HH): Behörde für Umwelt, Klima, Energie und Agrarwirtschaft	Water quality data, state of Hamburg Germany	https://www.hamburg.de/politik-und-verwaltung/behoerden/bukea	9	2000	2024
	Lower-Saxony (NI): Niedersächsischer Landesbetrieb für Wasserwirtschaft, Küsten- und Naturschutz (NLWKN)	Water quality data, state of Lower-Saxony Germany	https://datenbank.fgg-weser.de/weserdatenbank/#/download	2951	2000	2020
Global	GEMStat	Global Freshwater Quality for Water Resources Database and Global Change	https://gemstat.org/	4420	1980	2022

Country/Continent /Global	Source	Description	URL	Number of river stations by source	Start Year	End Year
	Glorich	GLObal RIver Chemistry of the University database of Hamburg	https://doi.pangaea.de/10.1594/PANGAEA.902360	27873	1942	2011
Guatemala	Ministerio de Comunicaciones Infraestructura Y Vivienda - Instituto Nacional de Sismología, Volcánología, Meteorología e Hidrologia	Water quality in Guatemala rivers, government institute	https://insivumeh.gob.gt/	92	1981	2012
India	India-WRIS	Water quality in Indian rivers, government institute	https://indiawris.gov.in/wris/#/	2407	1990	2018
Mexico	Comisión Nacional del Agua	Water quality data for surface locations in Mexico, government institute	https://www.gob.mx/conagua	2111	2012	2021
	National Commission of Water - General Subdivision of Administration of Water	Water quality in Mexican rivers, government institute	https://www.proyectosmexico.gob.mx/en/how-mexican-infrastructure/investment-cycle/hydraulic/	10	1980	1996
New Zealand	National Institute of Water and Atmospheric Research	Water quality in New Zealand rivers, government institute	https://www.gbif.org/publisher/c58ac019-b413-	80	1980	2021

Country/Continent /Global	Source	Description	URL	Number of river stations by source	Start Year	End Year
			4613-8772-39e57acbeb8e			
	NIWA	Water quality in New Zealand rivers, government institute	https://niwa.co.nz/	33	1989	2019
Panama	Ministerio de Ambiente Autoridad Nacional del Ambiente	Water quality in Panamanian rivers, government institute	https://miambiente.gob.pa/	1	1980	1985
	Unidad de Calidad de Agua - Autoridad del Canal de Panamá	Water quality in Panamanian rivers, government institute	https://pancanal.com/recurso-hidrico/	53	2003	2010
Peru	General Directorate of Environmental Health	Water quality in Peruvian rivers, government institute	https://www.minam.gob.pe/calidadambiental/gestion-de-la-calidad-del-agua/	8	1980	1983
Philippines	Department of Environment and Natural Resources - Environmental Management Bureau	Water quality in Philippines rivers, government institute	https://denr.gov.ph/	7	1980	1999
Romania	ICPDR	International Commission for the Protection of the Danube River (ICPDR), Romania Europe	https://www.icpdr.org/about-icpdr/organisation/keeper-danube	117	1989	2018
Senegal	Société Nationale des Eaux du Sénégal (SONES)	Water quality in Senegal rivers, government institute	http://www.sones.sn/	5	1986	2000

Country/Continent /Global	Source	Description	URL	Number of river stations by source	Start Year	End Year
South Africa	Department of Water Affairs and Forestry - Institute for Water Quality Studies	Water quality in South Africa rivers, government institute	https://wisa.org.za/	273	1990	2010
	Department of Water and Sanitation - DWS	Water quality in South Africa rivers, government institute	https://nationalgovernment.co.za/units/view/49/department-of-water-and-sanitation-dws	1710	1960	2022
	United Nations	Water quality in South Africa rivers	https://sdg6data.org/es/country-or-area/South%20Africa	2	1980	1982
Spain	SAICA network	Water Quality Information System of Spanish rivers, government institute	https://www.miteco.gob.es/es/agua/temas/estado-y-calidad-de-las-aguas/aguas-superficiales/programas-seguimiento/saica.html	1231	2001	2023
	Agencia Catalana del Agua	ACA Water Quality Information System of Spanish rivers, government institute	https://aplicacions.aca.gencat.cat/sdim21/seleccioXarxes.do	200	2007	2024

Country/Continent /Global	Source	Description	URL	Number of river stations by source	Start Year	End Year
	Ecobill	Information System of Spanish rivers, government institute. CARIMED project	https://parcs.diba.cat/es/web/conservacio-de-la-biodiversitat/rius	90	1979	2024
Uruguay	Department of Environmental Evaluation	Water quality in Uruguayan rivers, government institute	https://www.gub.uy/ministerio-ambiente/politicas-y-gestion/informes-monitoreo-documentos-calidad-agua	5	1980	1994
USA	WQP	USGS Water Quality Portal for surface and groundwaters across the US	https://www.waterqualitydata.us/	37388	1980	2023

Table S2. Examples of unit conversion from parameters form in source data to the GlobSalt version; x1 and x2 are observation values before and after conversion, respectively.

Parameter	Source	Source form Unit	Unit GlobSalt	x1	n	Mx1	Mx2	x2
Nitrate	GLORICH	NO3N µmol L^{-1}	mg L^{-1}	210.268	1000	62.004	14.007	0.0475
Ammonium	GLORICH	NH4N µmol L^{-1}	mg L^{-1}	1.5	1000	18.039	14.007	0.0011
Calcium	GLORICH	Ca µmol L^{-1}	mg L^{-1}	12,154	1000	40.078	40.078	12.154
Magnesium	GLORICH	Mg µmol L^{-1}	mg L^{-1}	12,081	1000	24.305	24.305	12.081
Sodium	GLORICH	Na µmol L^{-1}	mg L^{-1}	11,802	1000	22.99	22.99	11.802
Potassium	GLORICH	K µmol L^{-1}	mg L^{-1}	11,642	1000	39.098	39.098	11.642
Silica	GLORICH	SiO2 µmol L^{-1}	mg L^{-1}	9,880	1000	60.086	28.086	4.618
Chloride	GLORICH	Cl µmol L^{-1}	mg L^{-1}	12,600	1000	35.453	35.453	12.6
Sulphate	GLORICH	SO4 µmol L^{-1}	mg L^{-1}	12	1000	96.062	32.06	0.0040

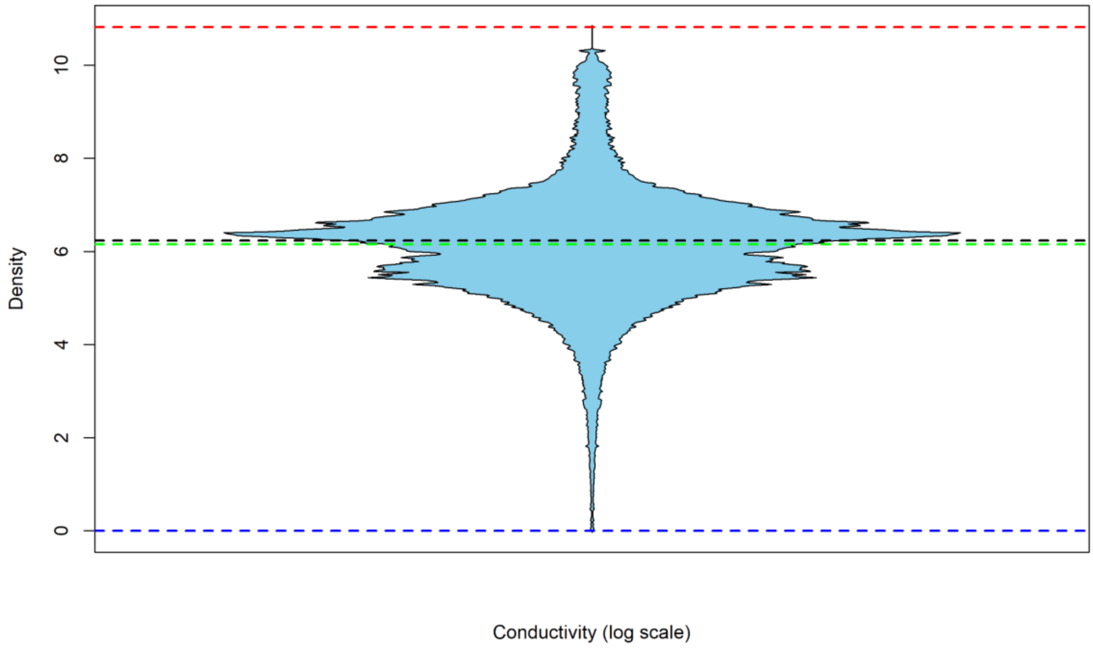

Figure S1. Beanplot of the Conductivity Distribution in GlobSalt. The distribution was adjusted for a log-normal fit, including quartiles and outliers as per this method. The black, green, blue, and red lines represent the median, mean, minimum, and maximum of the data.

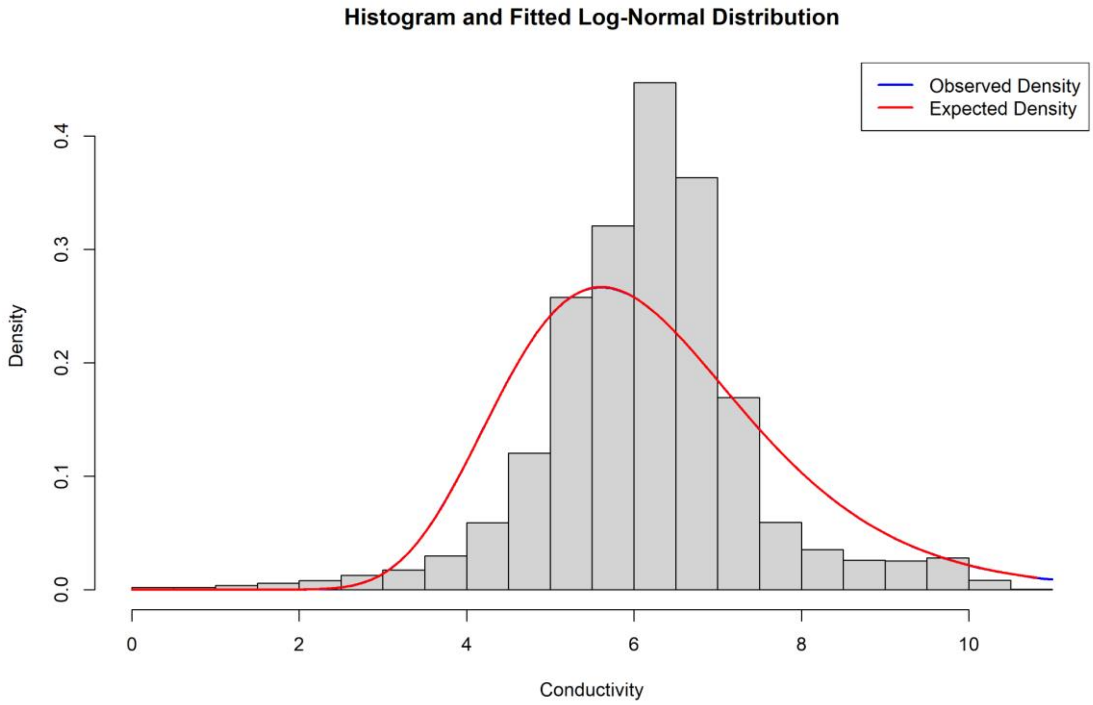

Figure S2. Histogram plot showing both the observed density (blue line) and the expected density (red line) of the conductivity distribution in GlobSalt, along with the fitted log-normal distribution.

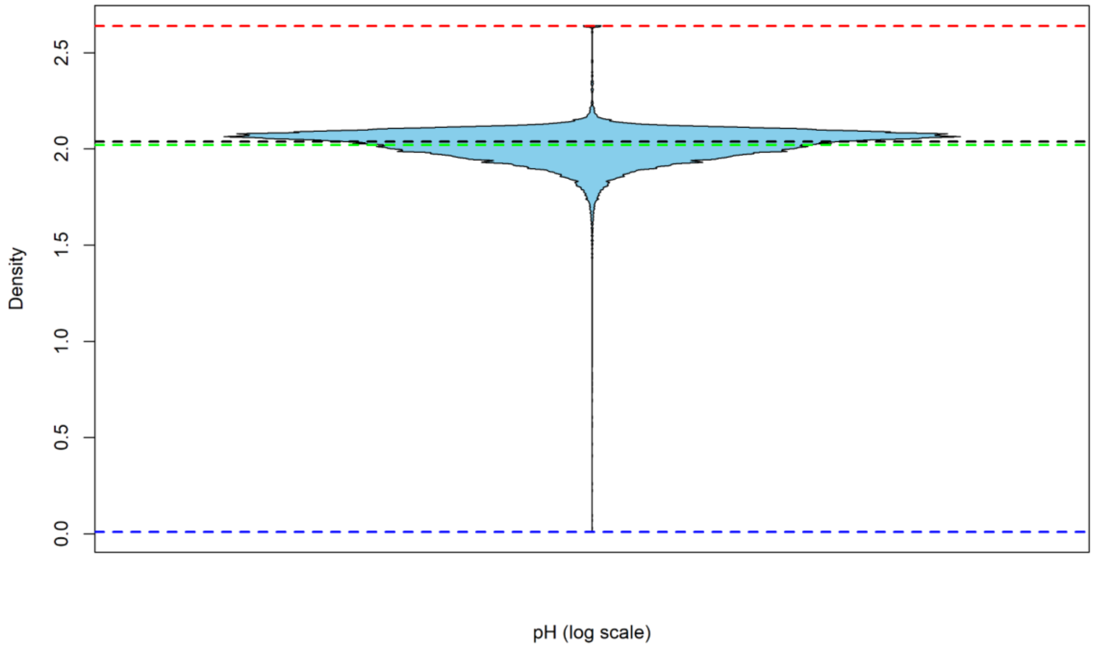

Figure S3. Beanplot of the pH Distribution in GlobSalt. The distribution was adjusted for a log-normal fit, including quartiles and outliers as per this method. The black, green, blue, and red lines represent the median, mean, minimum, and maximum of the data.

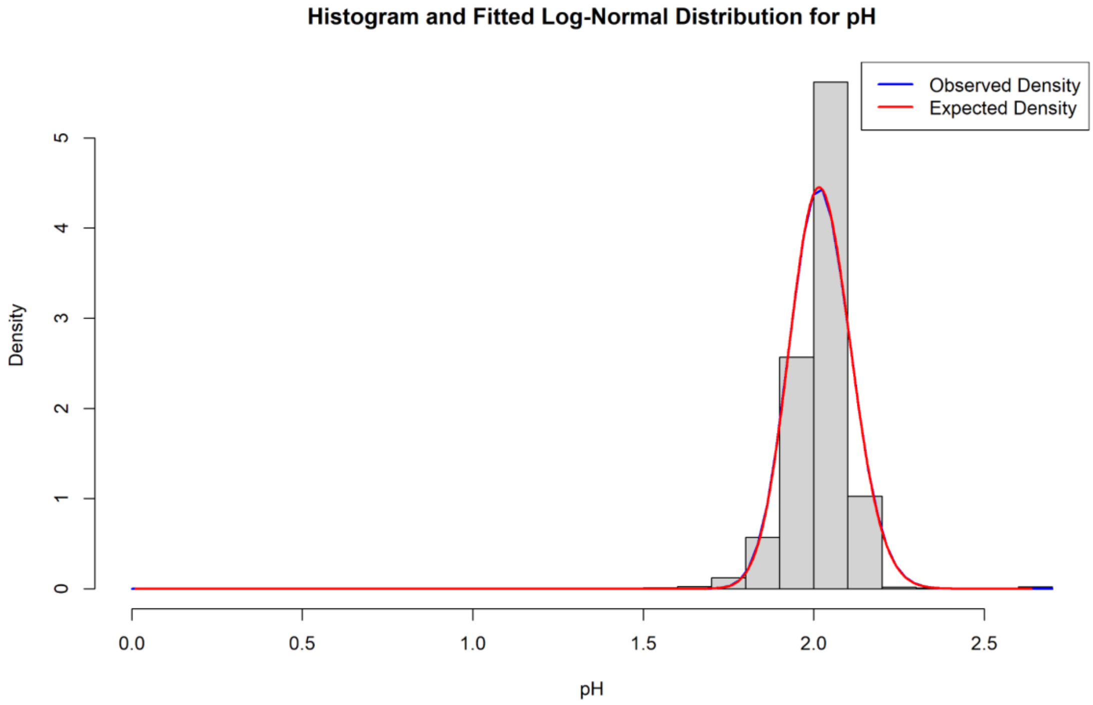

Figure S4. Histogram plot showing both the observed density (blue line) and the expected density (red line) of the pH distribution in GlobSalt, along with the fitted log-normal distribution.

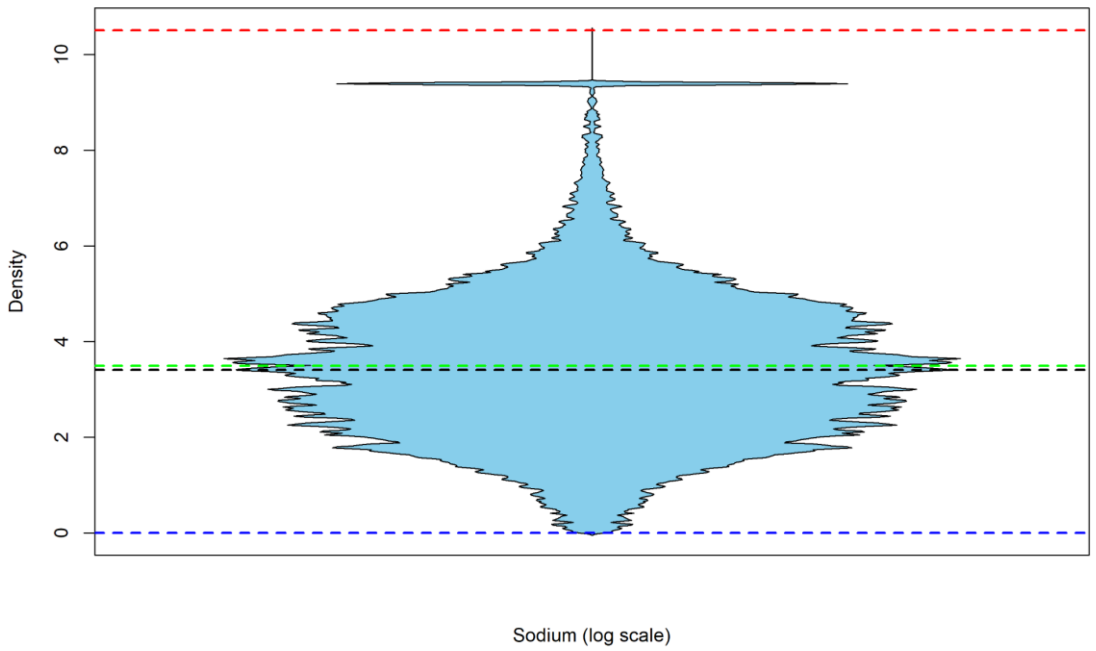

Figure S5. Beanplot of the sodium Distribution in GlobSalt. The distribution was adjusted for a log-normal fit, including quartiles and outliers as per this method. The black, green, blue, and red lines represent the median, mean, minimum, and maximum of the data.

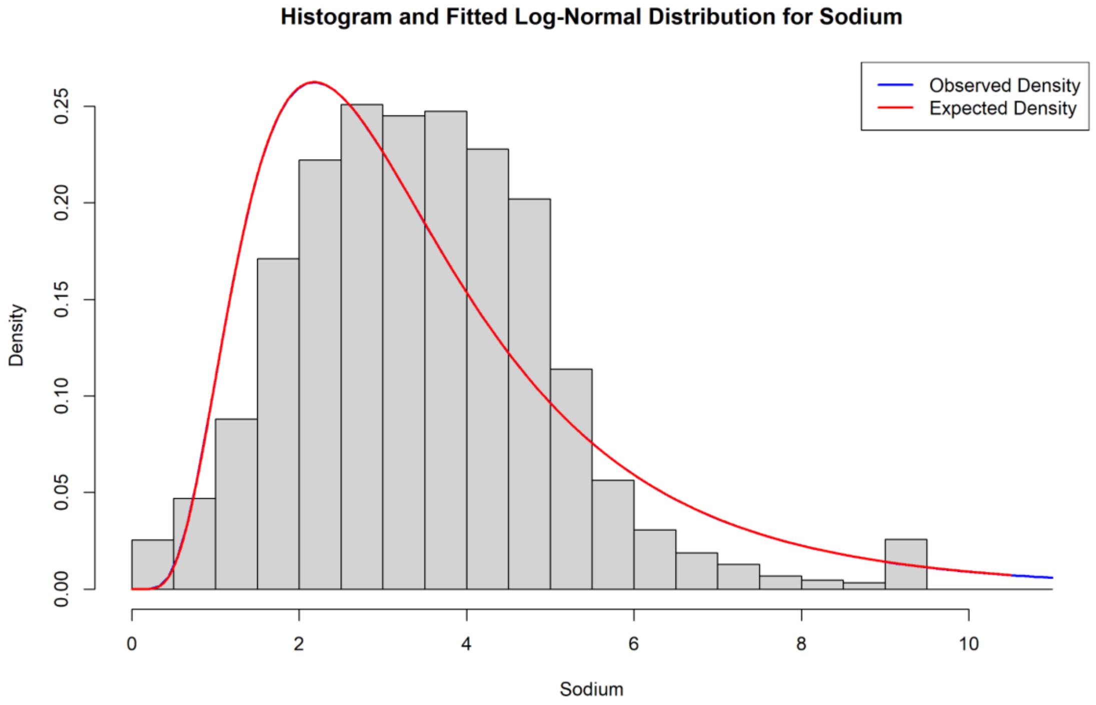

Figure S6. Histogram plot showing both the observed density (blue line) and the expected density (red line) of the sodium distribution in GlobSalt, along with the fitted log-normal distribution.

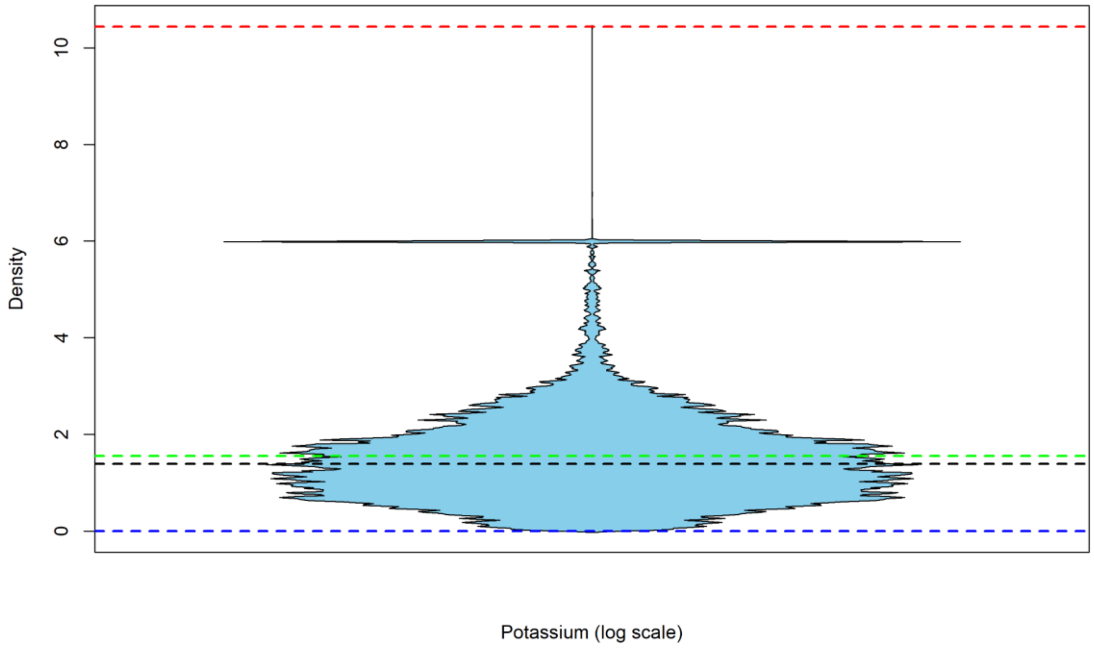

Figure S7. Beanplot of the potassium Distribution in GlobSalt. The distribution was adjusted for a log-normal fit, including quartiles and outliers as per this method. The black, green, blue, and red lines represent the median, mean, minimum, and maximum of the data.

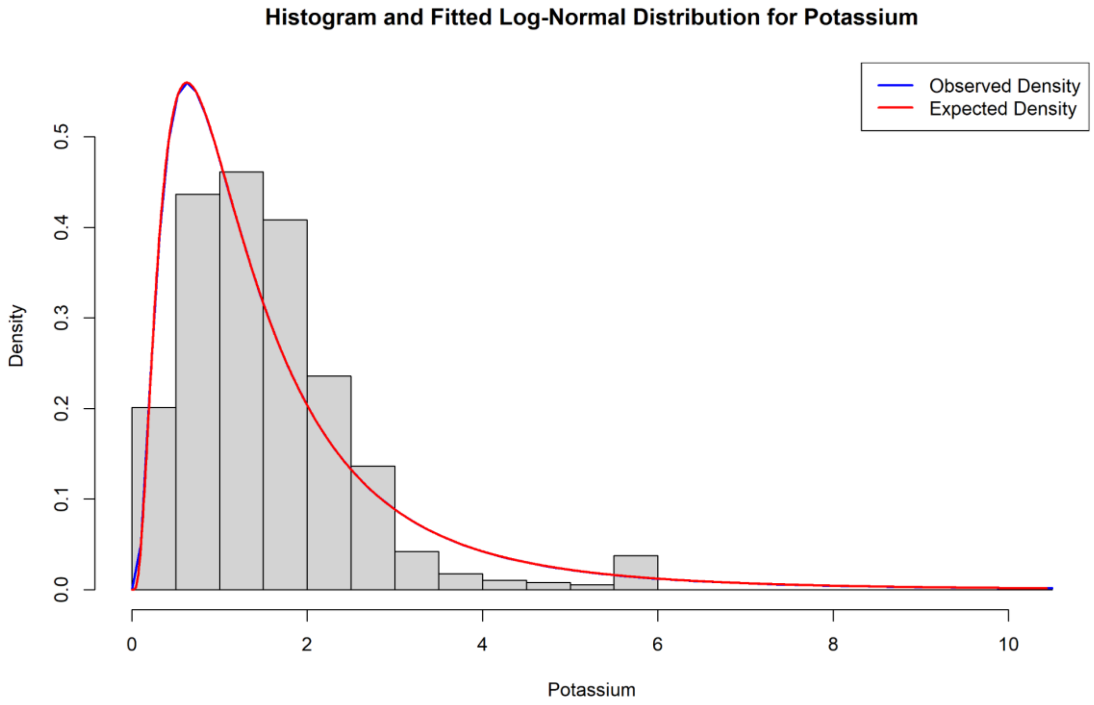

Figure S8. Histogram plot showing both the observed density (blue line) and the expected density (red line) of the potassium distribution in GlobSalt, along with the fitted log-normal distribution.

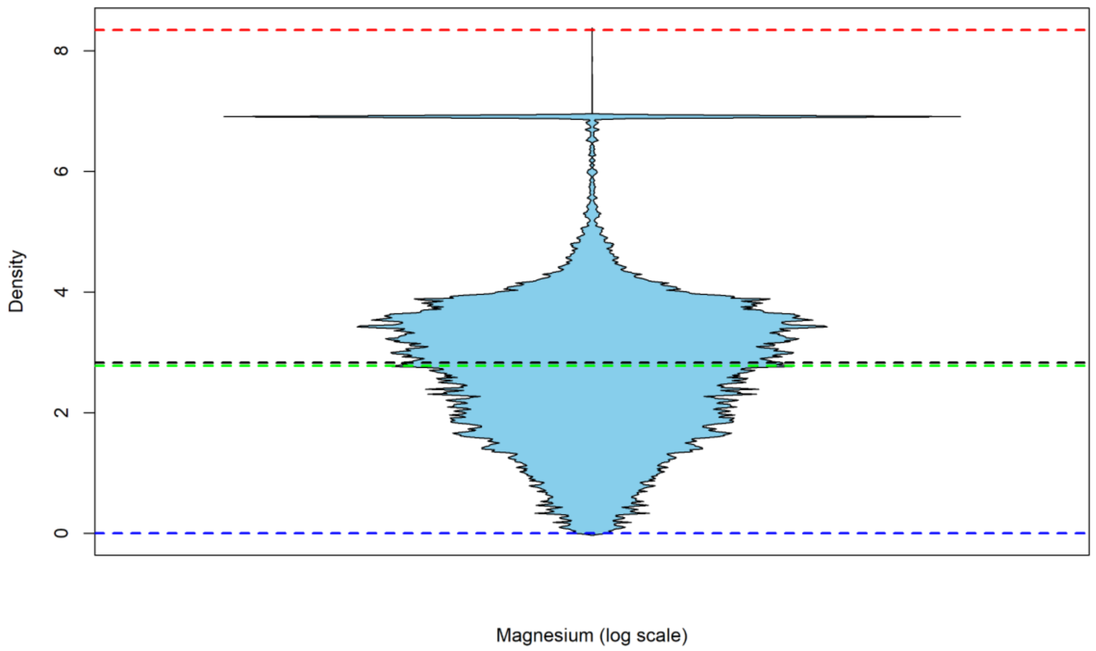

Figure S9. Beanplot of the magnesium Distribution in GlobSalt. The distribution was adjusted for a log-normal fit, including quartiles and outliers as per this method. The black, green, blue, and red lines represent the median, mean, minimum, and maximum of the data.

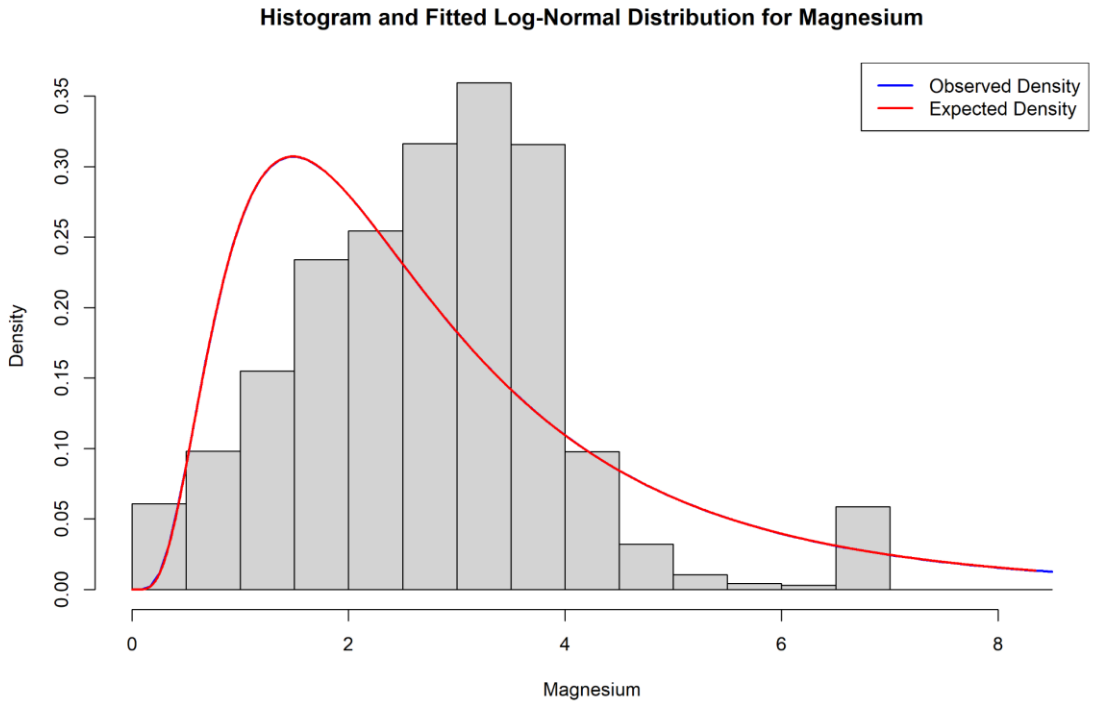

Figure S10. Histogram plot showing both the observed density (blue line) and the expected density (red line) of the magnesium distribution in GlobSalt, along with the fitted log-normal distribution.

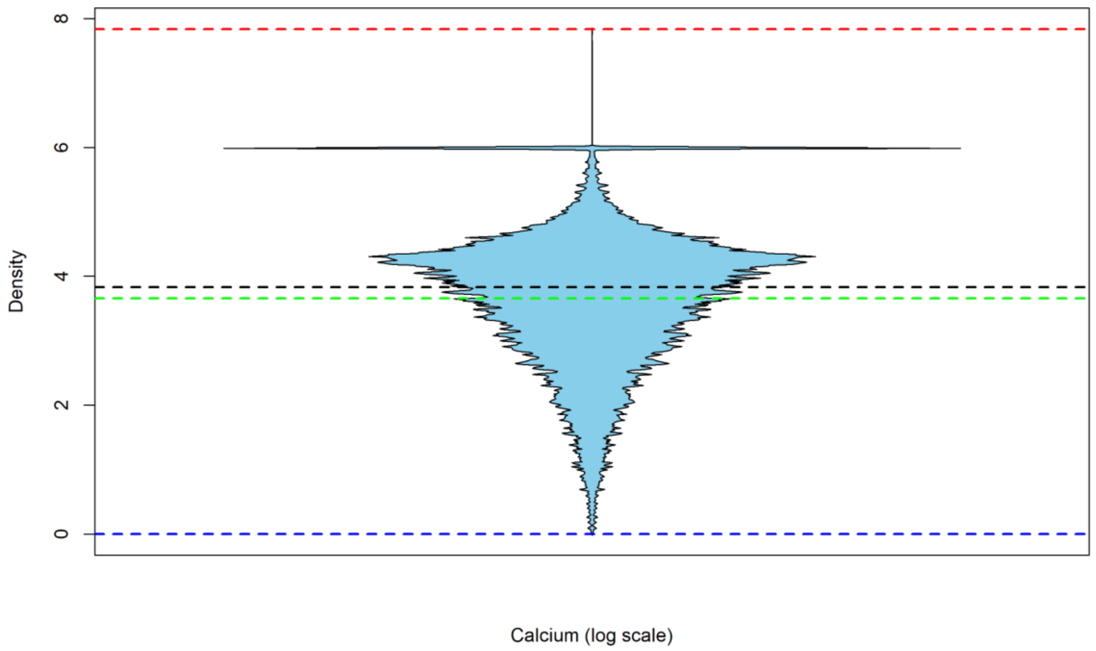

Figure S11. Beanplot of the Calcium Distribution in GlobSalt. The distribution was adjusted for a log-normal fit, including quartiles and outliers as per this method. The black, green, blue, and red lines represent the median, mean, minimum, and maximum of the data.

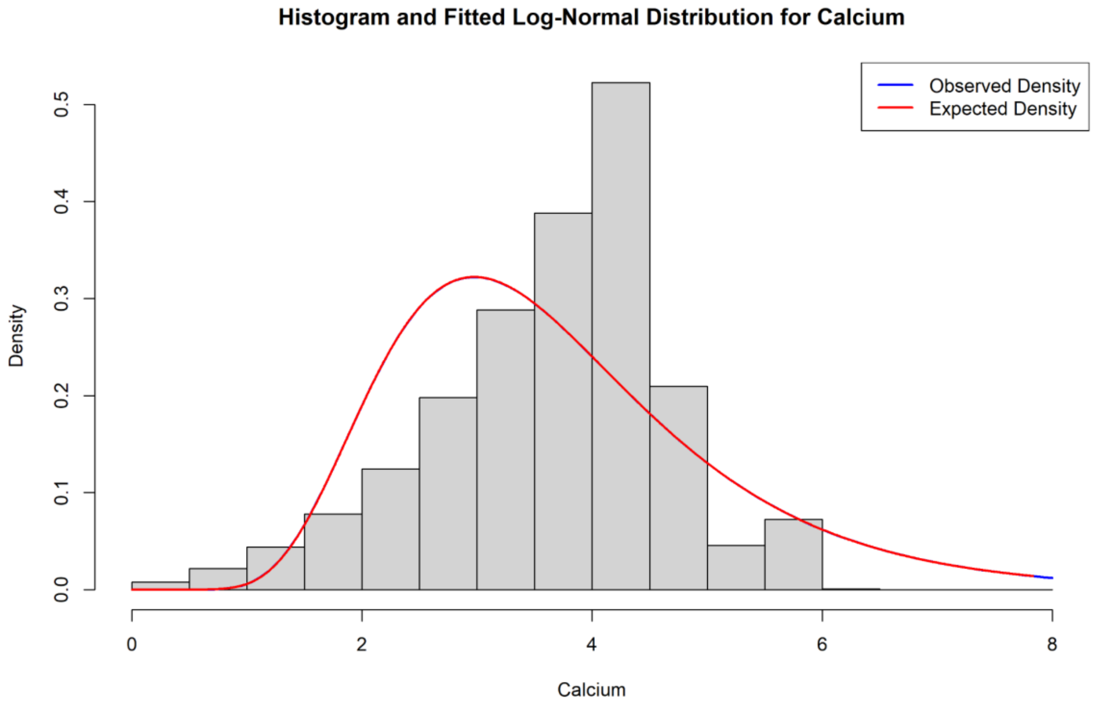

Figure S12. Histogram plot showing both the observed density (blue line) and the expected density (red line) of the calcium distribution in GlobSalt, along with the fitted log-normal distribution.

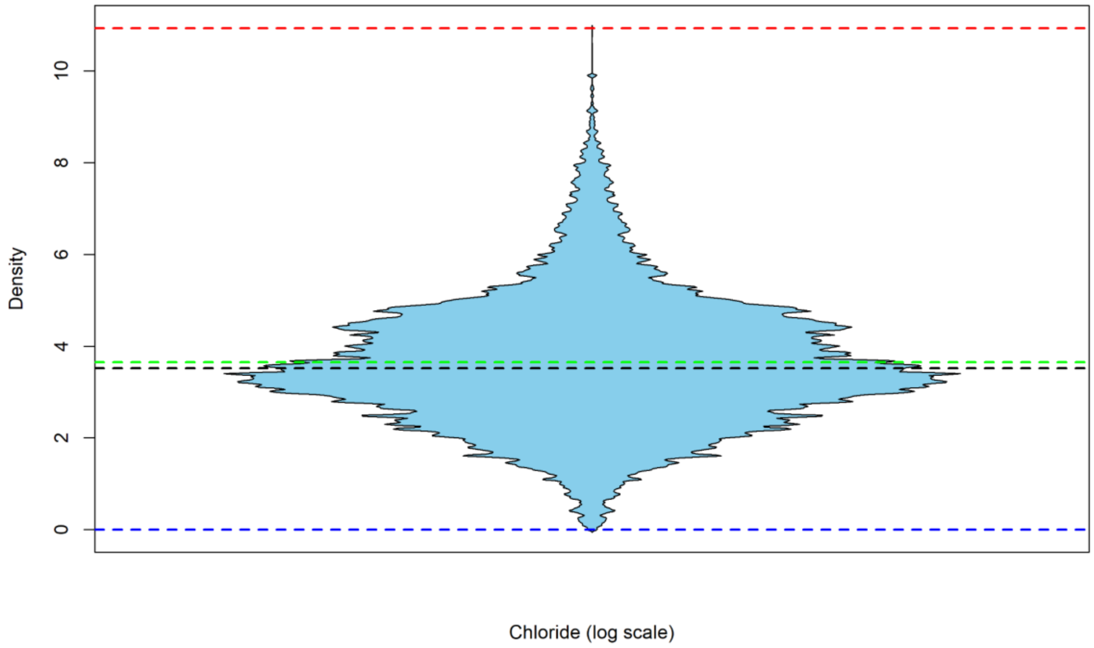

Figure S13. Beanplot of the Chloride Distribution in GlobSalt. The distribution was adjusted for a log-normal fit, including quartiles and outliers as per this method. The black, green, blue, and red lines represent the median, mean, minimum, and maximum of the data.

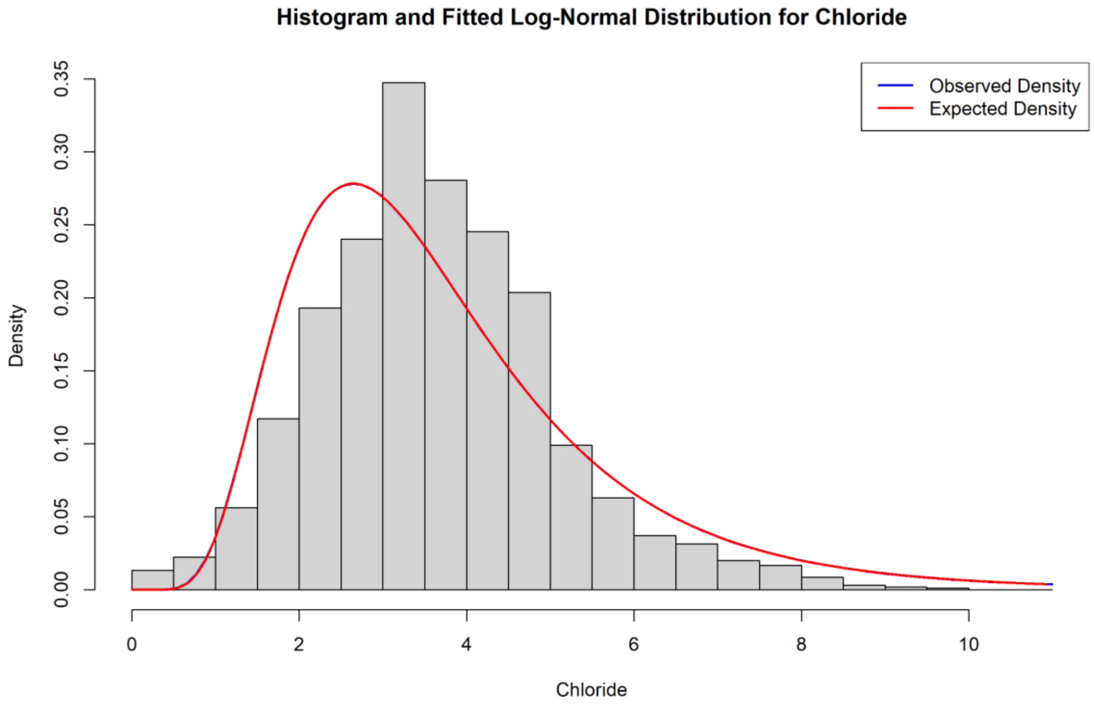

Figure S14. Histogram plot showing both the observed density (blue line) and the expected density (red line) of the chloride distribution in GlobSalt, along with the fitted log-normal distribution.

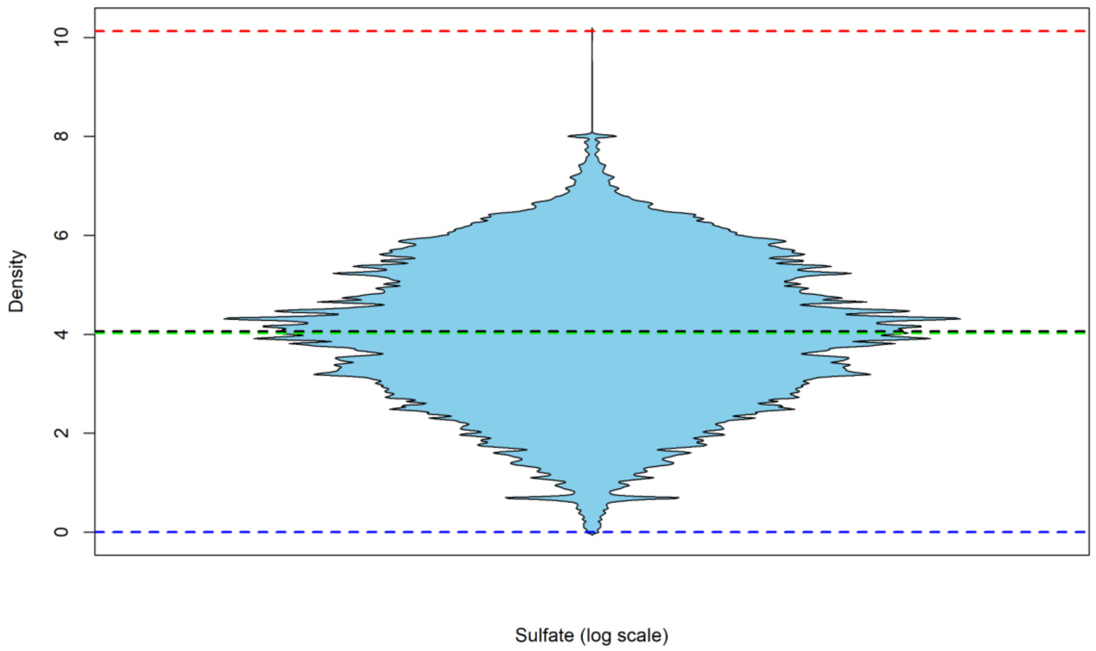

Figure S15. Beanplot of the Sulphate Distribution in GlobSalt. The distribution was adjusted for a log-normal fit, including quartiles and outliers as per this method. The black, green, blue, and red lines represent the median, mean, minimum, and maximum of the data.

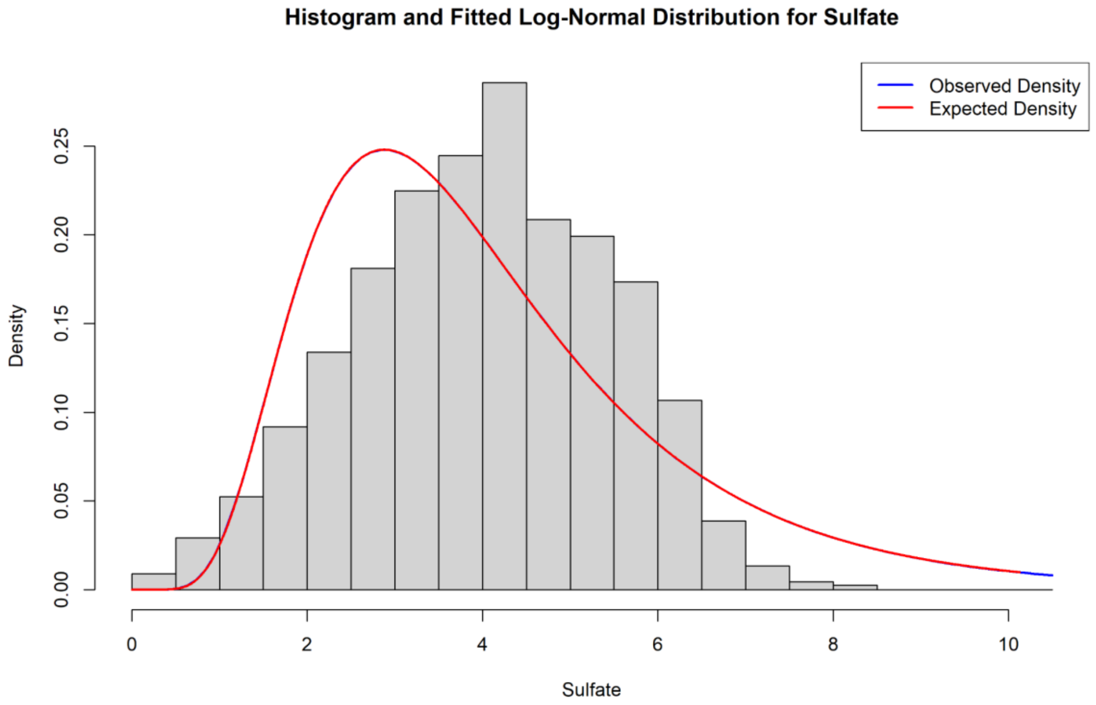

Figure S16. Histogram plot showing both the observed density (blue line) and the expected density (red line) of the sulphate distribution in GlobSalt, along with the fitted log-normal distribution.

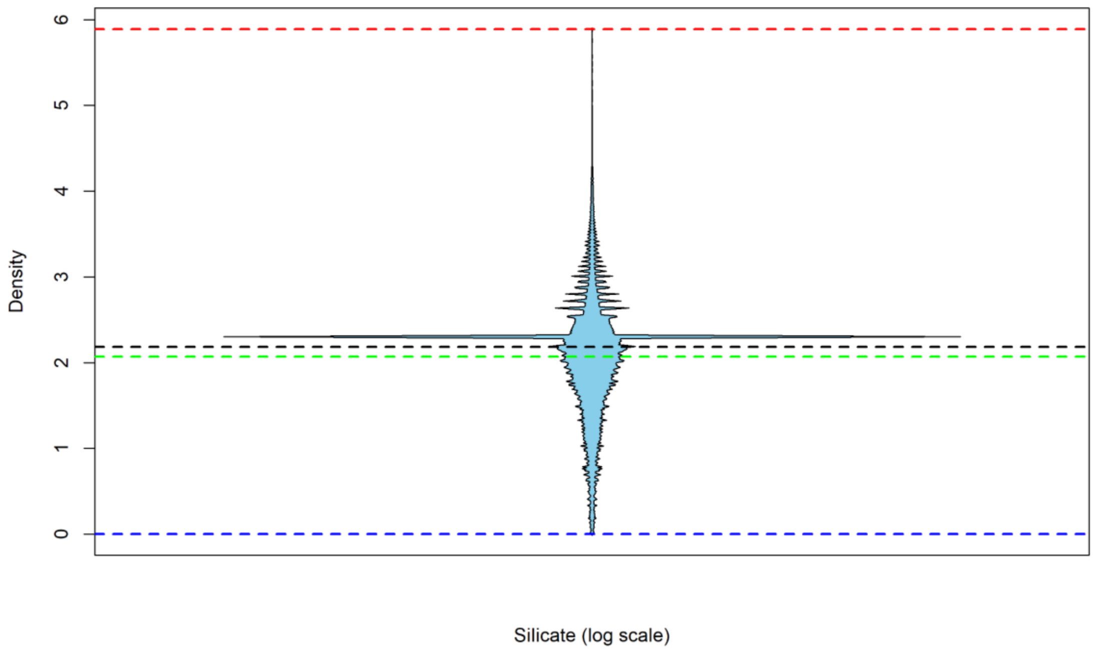

Figure S17. Beanplot of the Silicate Distribution in GlobSalt. The distribution was adjusted for a log-normal fit, including quartiles and outliers as per this method. The black, green, blue, and red lines represent the median, mean, minimum, and maximum of the data.

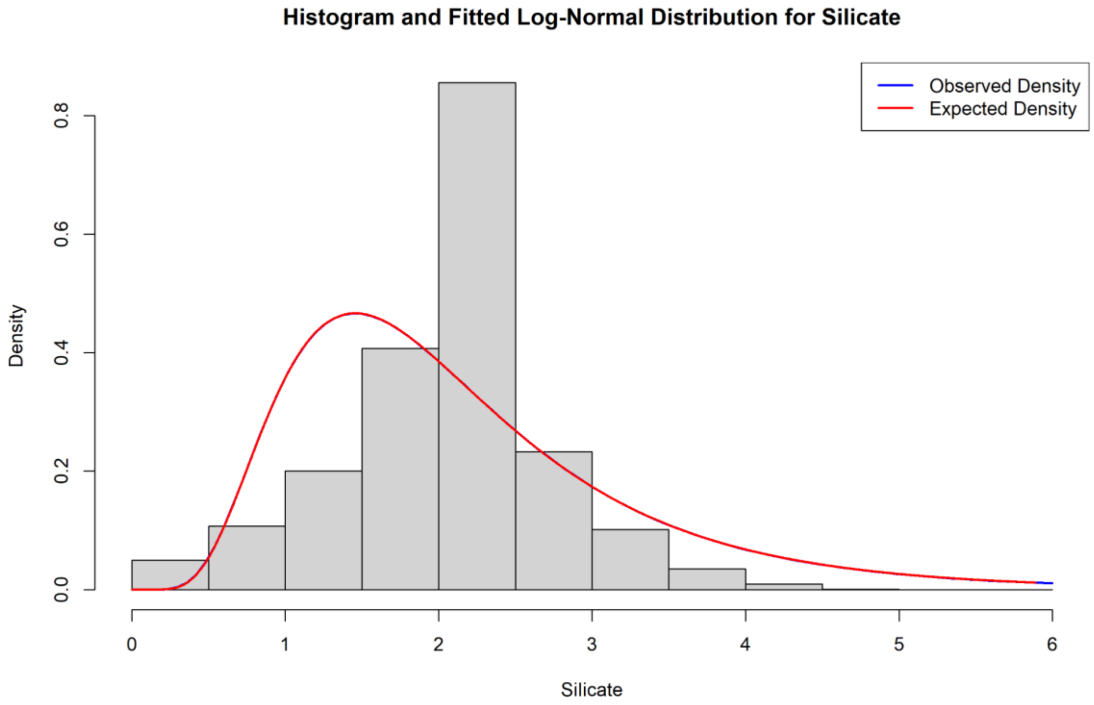

Figure S18. Histogram plot showing both the observed density (blue line) and the expected density (red line) of the silicate distribution in GlobSalt, along with the fitted log-normal distribution.

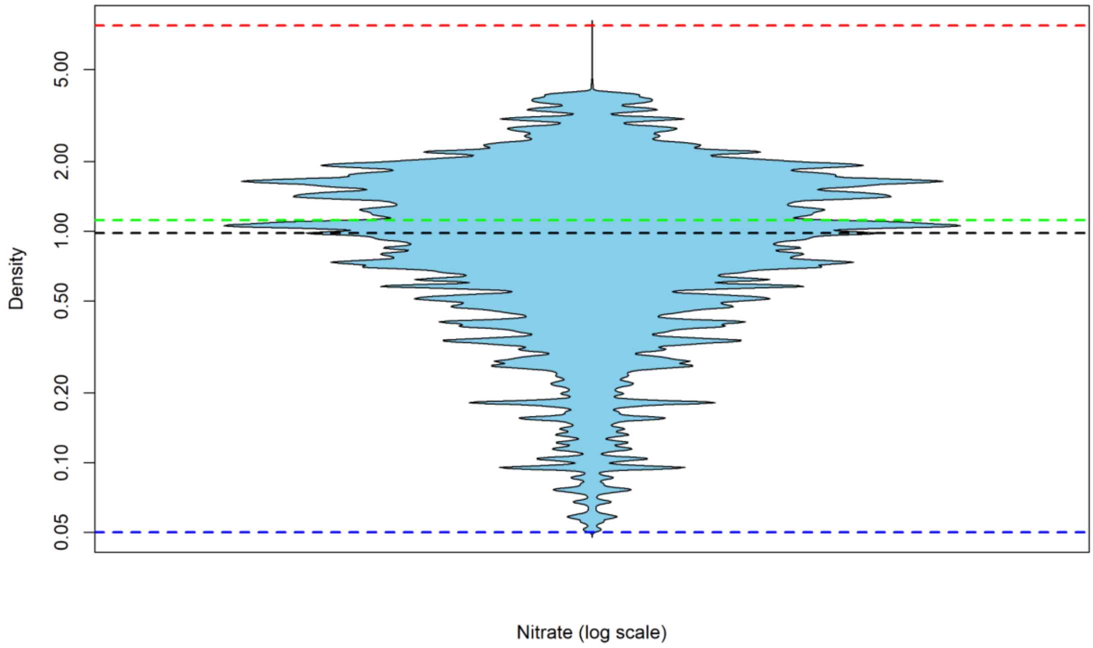

Figure S19. Beanplot of the Nitrate Distribution in GlobSalt. The distribution was adjusted for a log-normal fit, including quartiles and outliers as per this method. The black, green, blue, and red lines represent the median, mean, minimum, and maximum of the data.

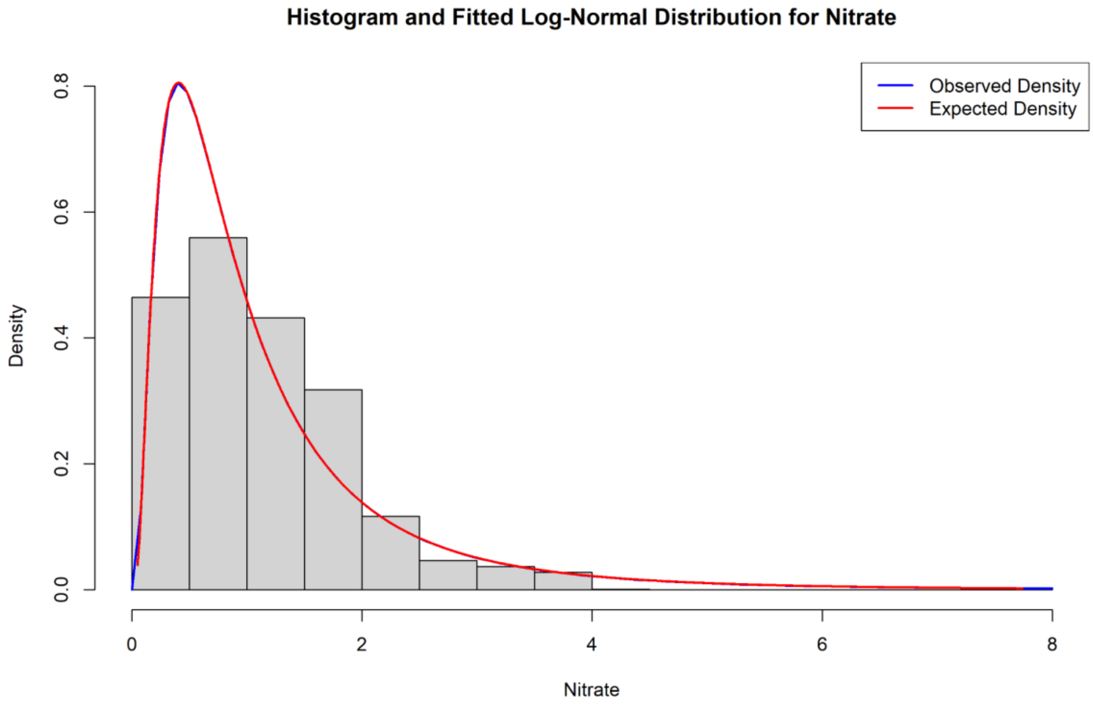

Figure S20. Histogram plot showing both the observed density (blue line) and the expected density (red line) of the nitrate distribution in GlobSalt, along with the fitted log-normal distribution.

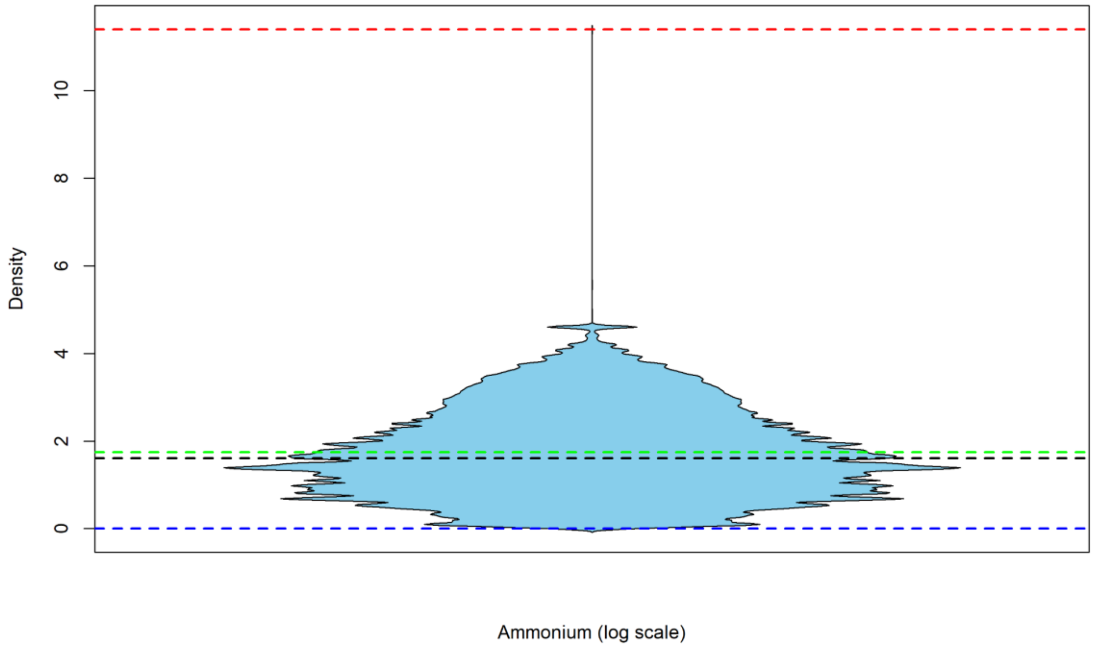

Figure S21. Beanplot of the Ammonium Distribution in GlobSalt. The distribution was adjusted for a log-normal fit, including quartiles and outliers as per this method. The black, green, blue, and red lines represent the median, mean, minimum, and maximum of the data.

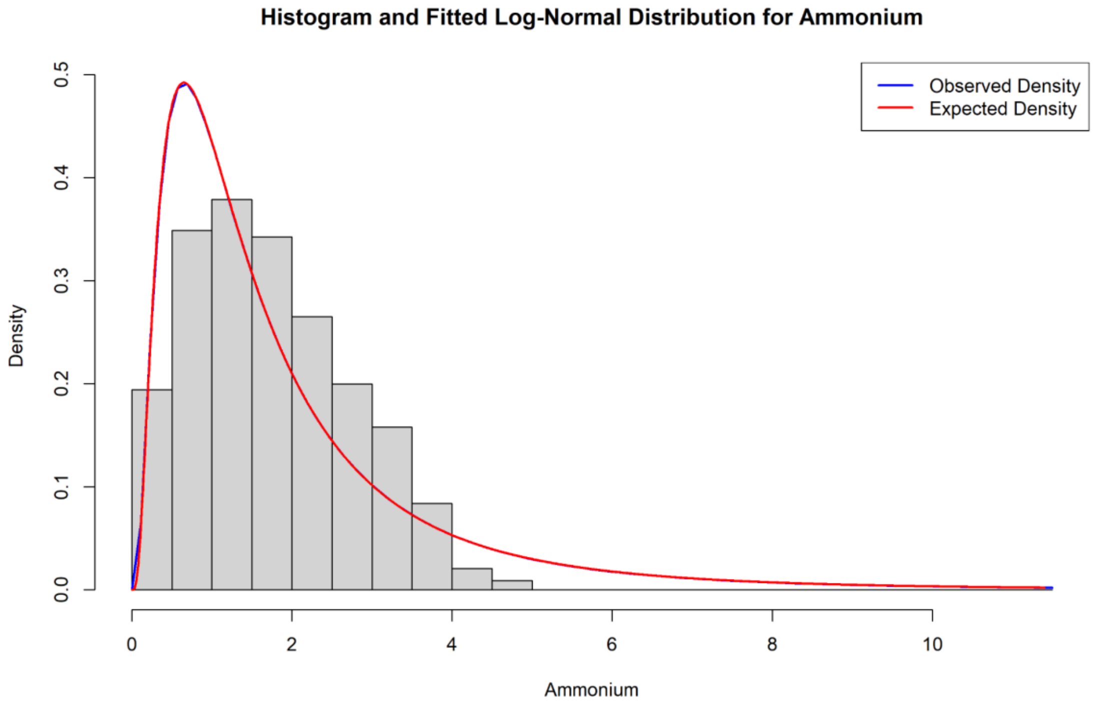

Figure S22. Histogram plot showing both the observed density (blue line) and the expected density (red line) of the ammonium distribution in GlobSalt, along with the fitted log-normal distribution.

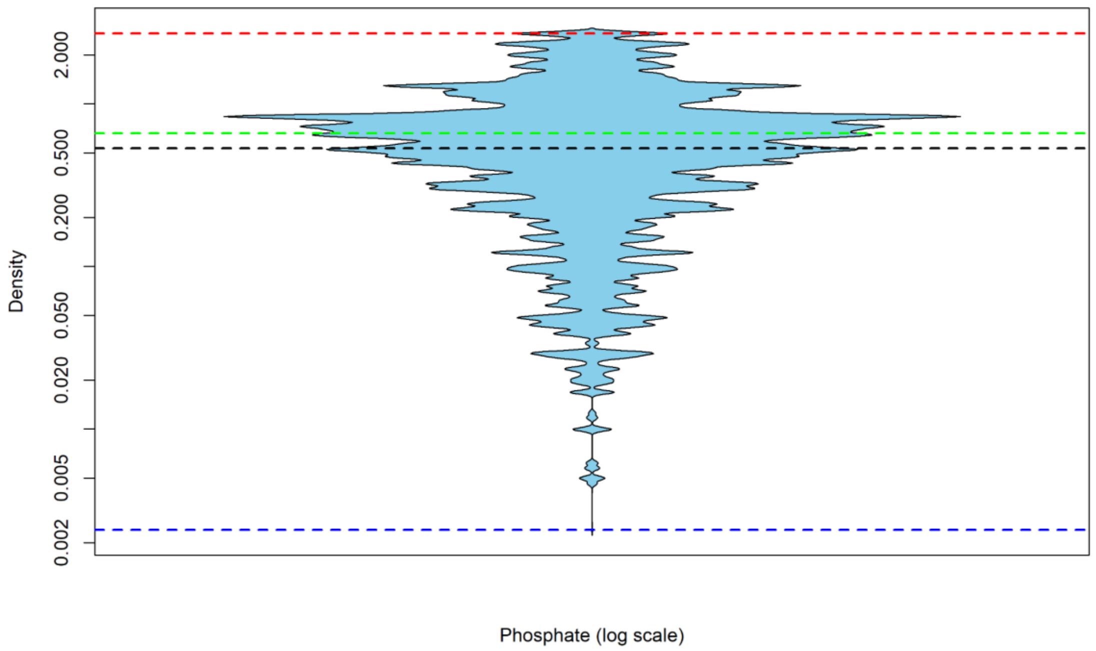

Figure S23. Beanplot of the Phosphate Distribution in GlobSalt. The distribution was adjusted for a log-normal fit, including quartiles and outliers as per this method. The black, green, blue, and red lines represent the median, mean, minimum, and maximum of the data.

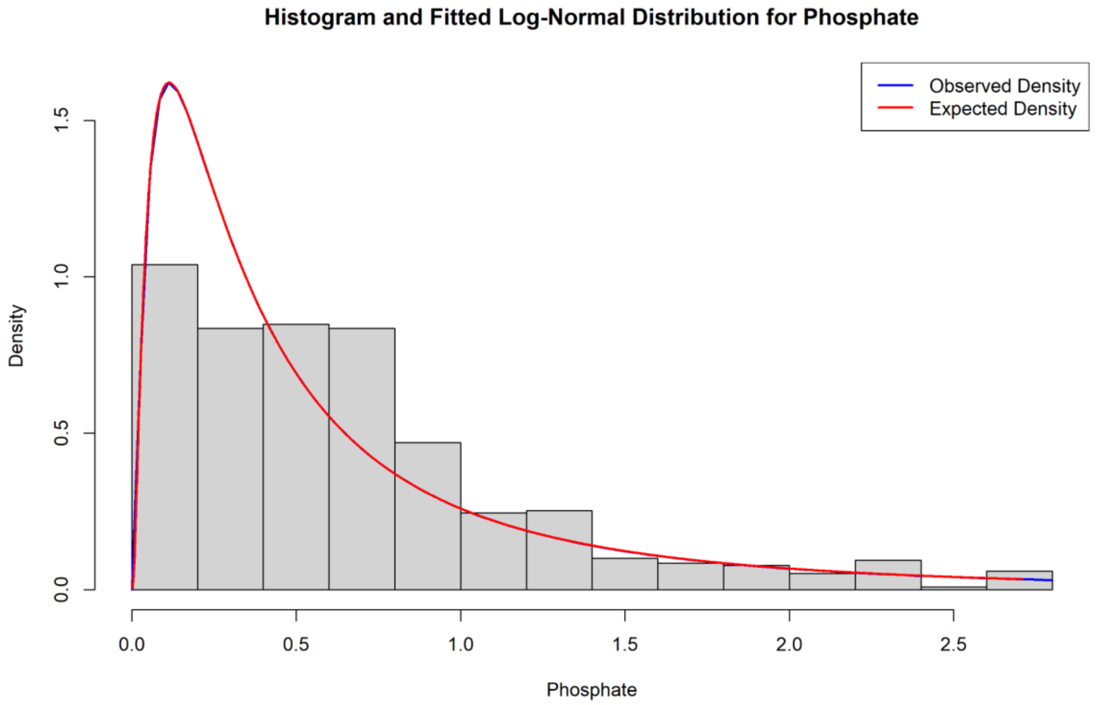

Figure S24. Histogram plot showing both the observed density (blue line) and the expected density (red line) of the phosphate distribution in GlobSalt, along with the fitted log-normal distribution.

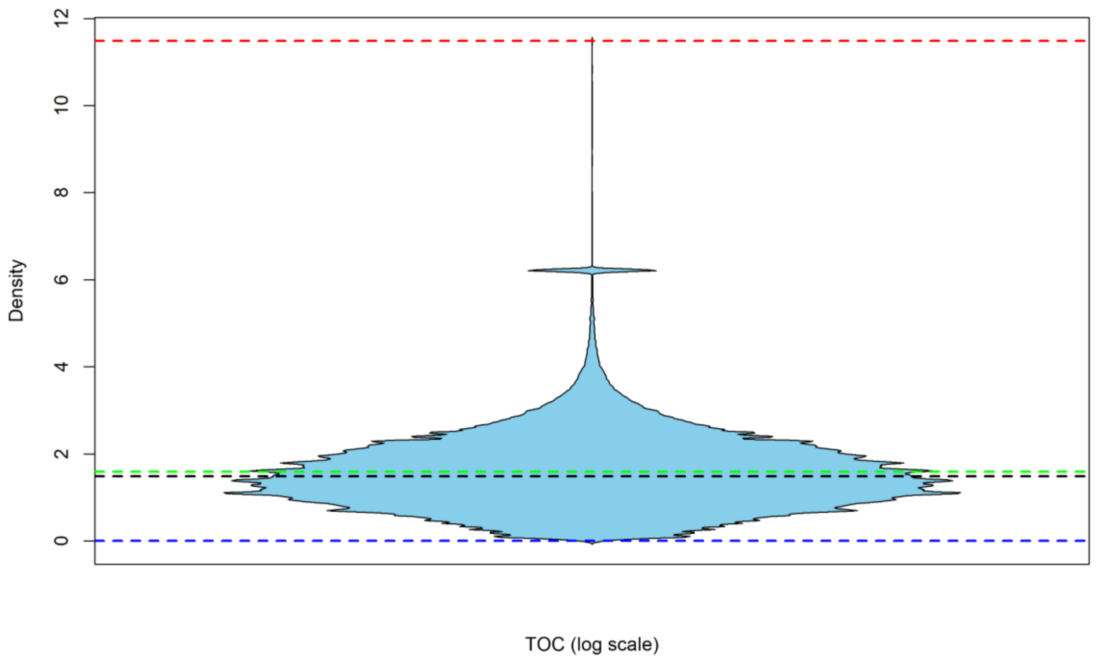

Figure S25. Beanplot of the Total Organic Carbon (TOC) Distribution in GlobSalt. The distribution was adjusted for a log-normal fit, including quartiles and outliers as per this method. The black, green, blue, and red lines represent the median, mean, minimum, and maximum of the data.

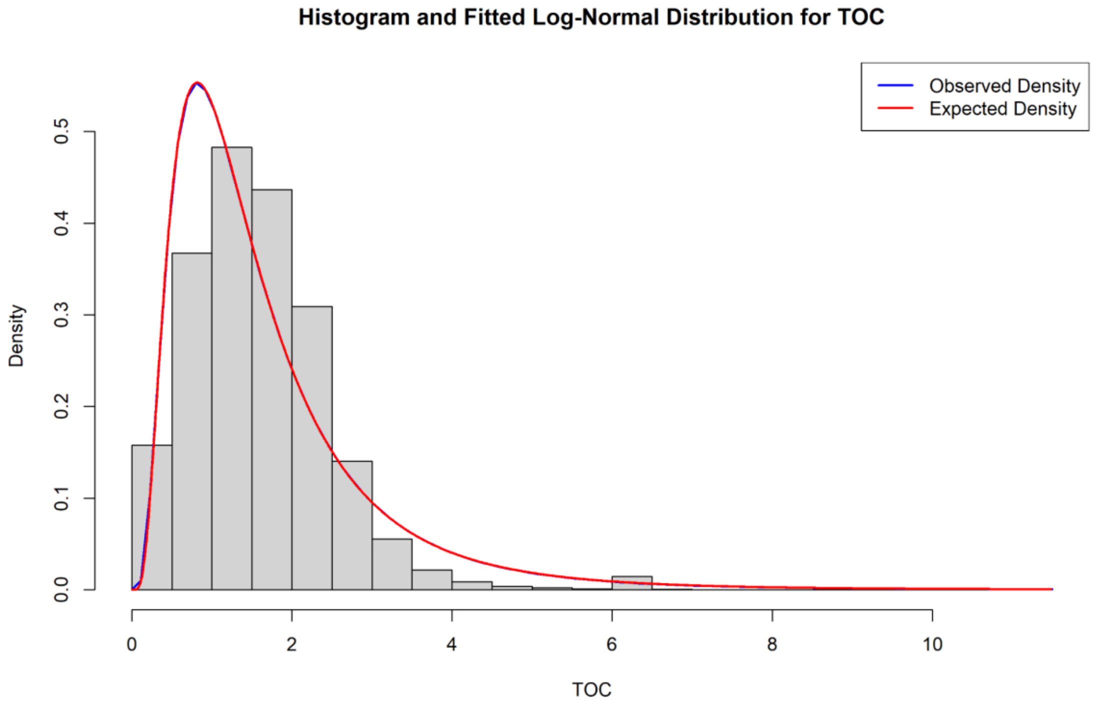

Figure S26. Histogram plot showing both the observed density (blue line) and the expected density (red line) of the Total Organic Carbon (TOC) distribution in GlobSalt, along with the fitted log-normal distribution.

GlobSalt data catalog

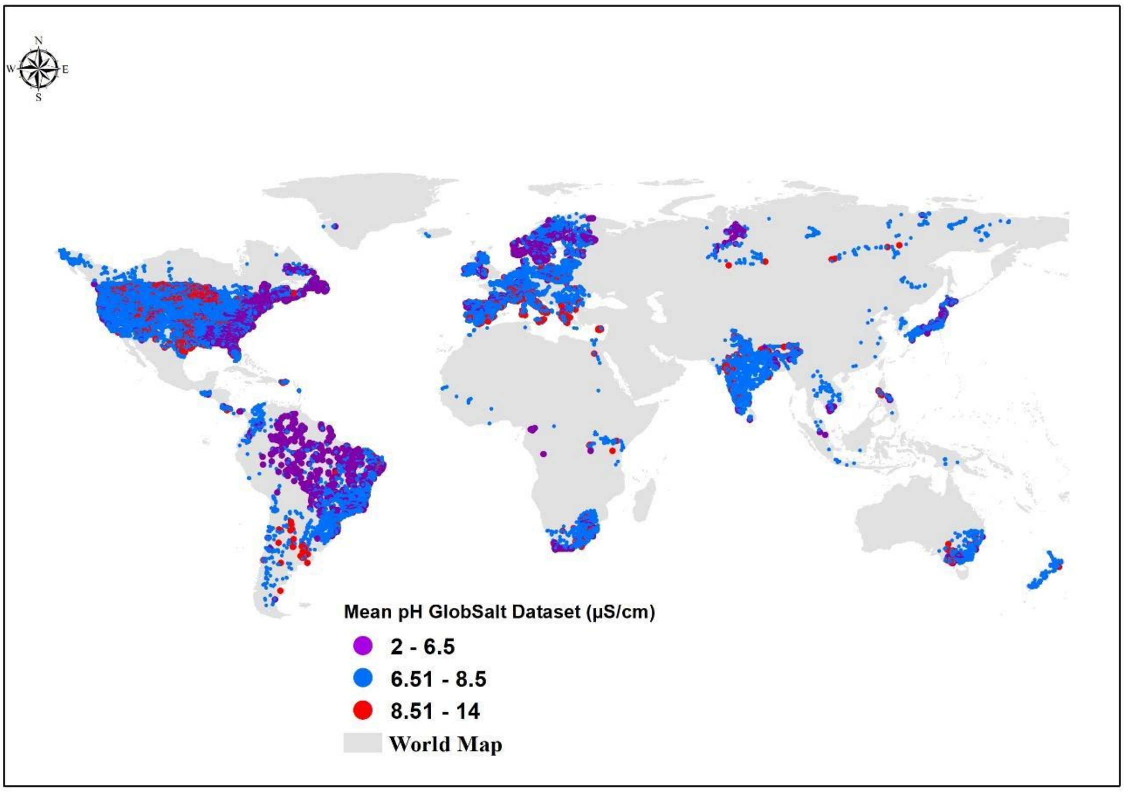

Figure C1. Global distribution of measured mean pH and station density. The global map in the panel shows the measured mean pH values per river in each country, based on observations included in the GlobSalt database, over the entire data period (1980-2023). Purple, blue, and red dots represent pH levels as follows: purple for acidic (low) pH (2-6.5), blue for neutral to slightly alkaline pH (6.5-8.5), and red for basic (high) pH (8.5-14), respectively.

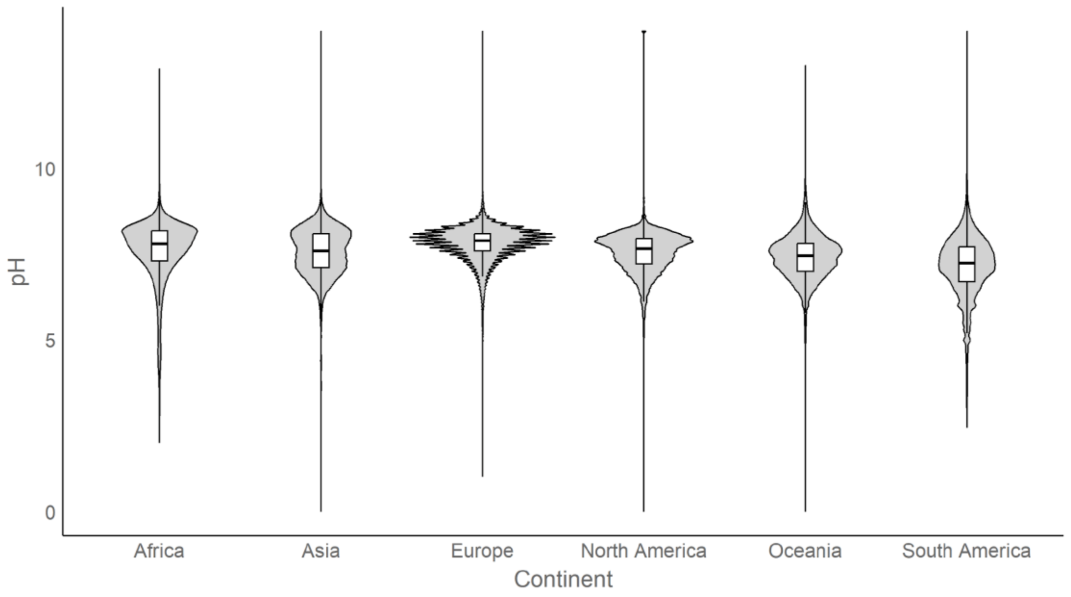

Figure C2. Global violin plot of pH by continent. Global Violin Plot of pH by Continent. EC observations are included in the GlobSalt database, over the entire data period (1980-2023). Each gray violin plot represents the available data set according to the number (n) of observations per continent (Africa n = 222081, Asia n= 146318, Europe n = 862589, North America n = 9657892, Oceania n = 94118, South America n = 67352).

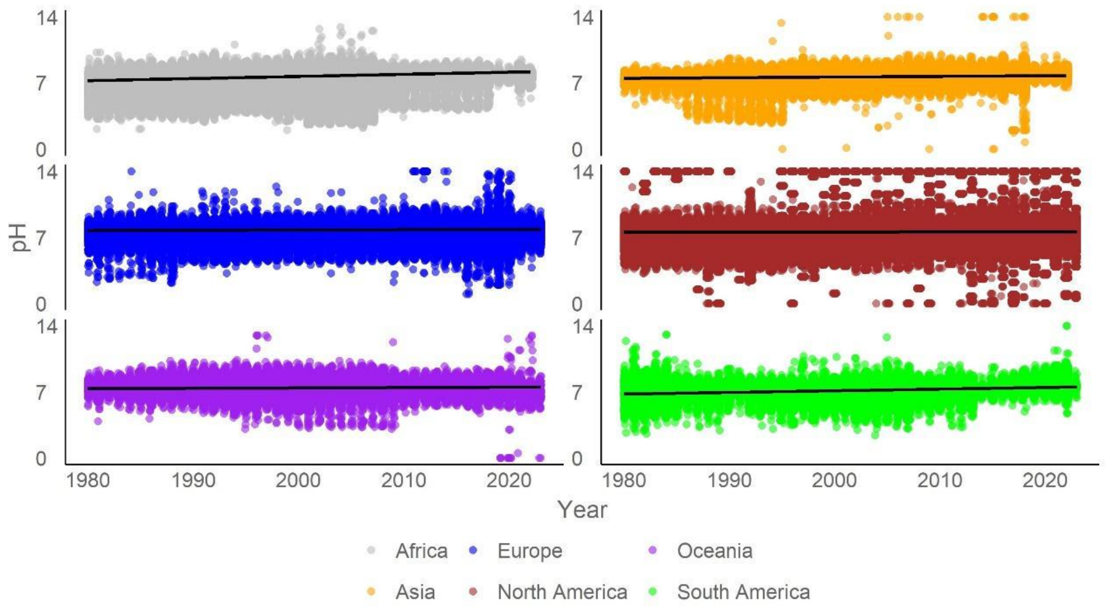

Figure C3. Global temporal dispersion plot pH by continent. pH observations are included in the GlobSalt database over the entire data period (1980-2023). Each color point (gray, yellow, blue, brown, purple, and green) represents the number (n) of observations per continent (Africa n = 222081, Asia n= 146318, Europe n = 862589, North America n = 9657892, Oceania n = 94118, South America n = 67352).

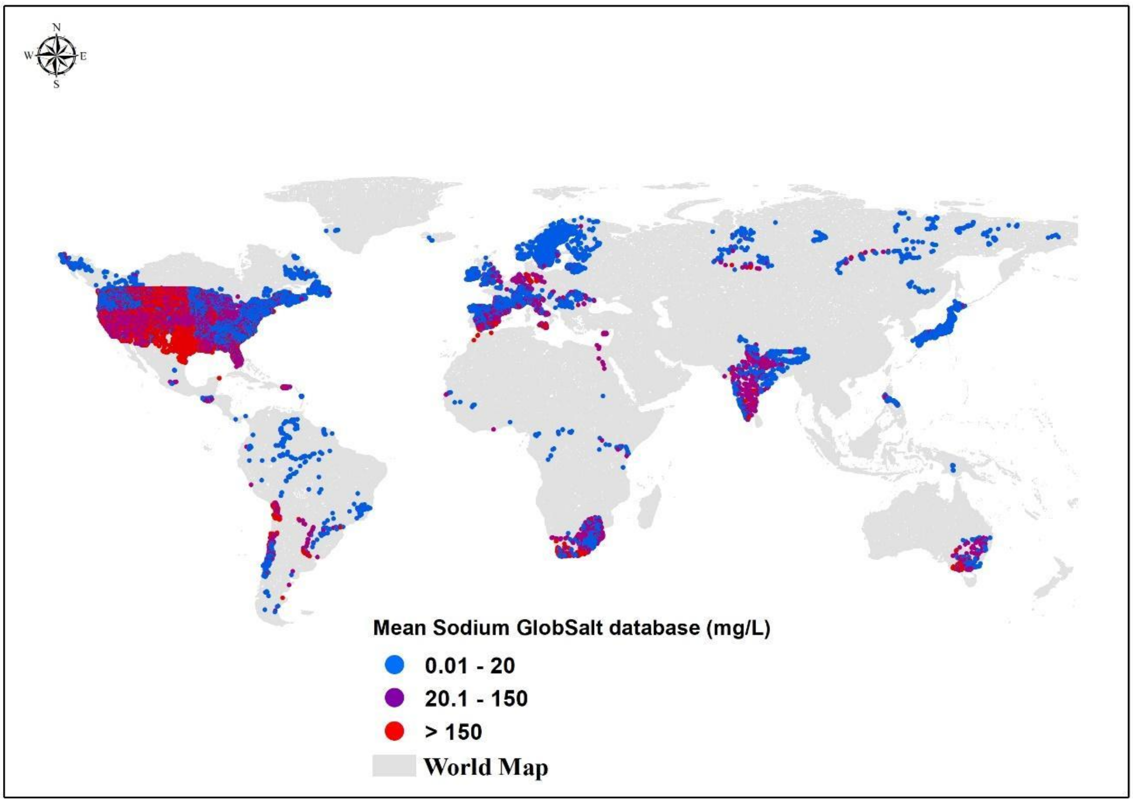

Figure C4. Global distribution of measured mean dissolved sodium and station density. The global map in the panel shows the measured mean sodium values per river in each country, based on observations included in the GlobSalt database, over the entire data period (1980-2023). Ble, purple, and red dots represent low, moderate, and high sodium, respectively.

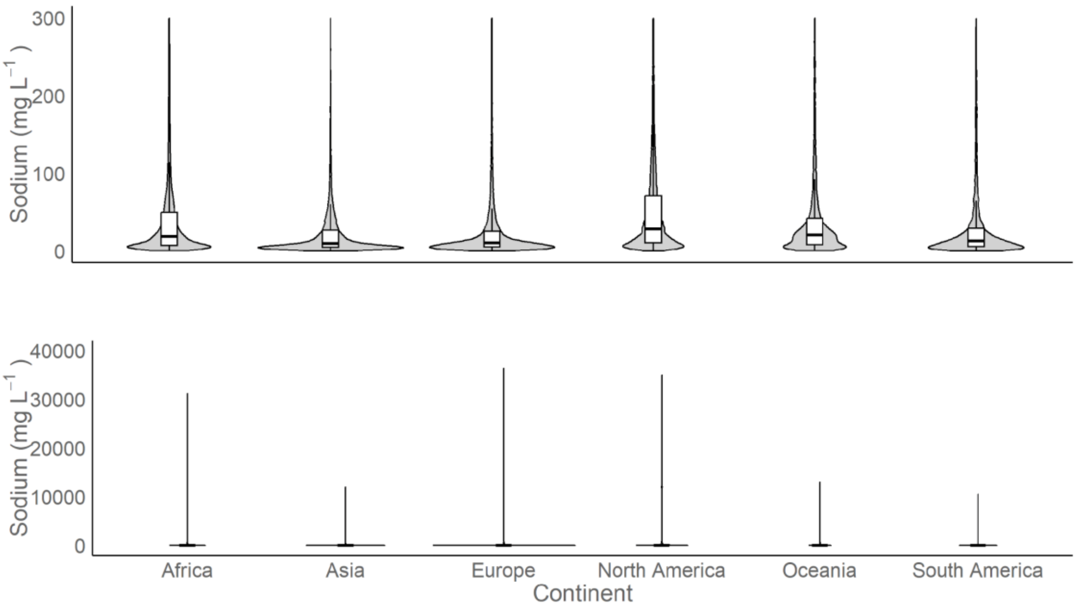

Figure C5. Global violin plot of dissolved sodium by continent. Top: Global Violin Plot of sodium by Continent with Zoom to 300 mg L^{-1}. Bottom: Global Violin Plot sodium across entire scale by Continent. Sodium observations are included in the GlobSalt database, over the entire data period (1980-2023). Each gray violin plot represents the available data set according to the number (n) of observations per continent (Africa n = 133687, Asia n= 100698, Europe n = 204582, North America n = 5029090, Oceania n = 9797, South America n = 15582).

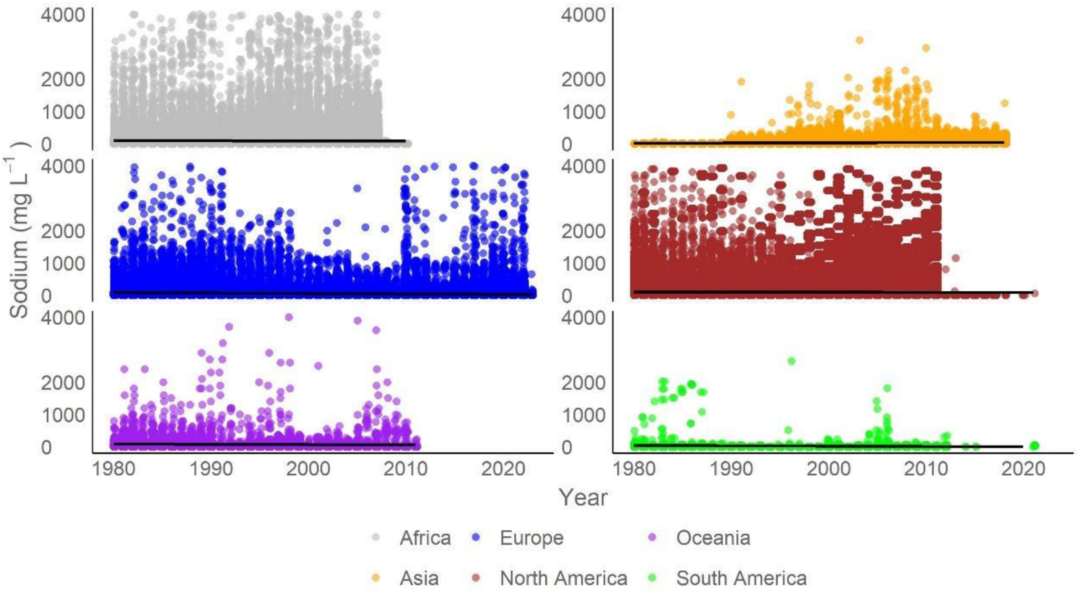

Figure C6. Global temporal dispersion plot of dissolved sodium by continent. Sodium observations are included in the GlobSalt database over the entire data period (1980-2023). Each color point (gray, yellow, blue, brown, purple, and green) represents the number (n) of observations per continent (Africa n = 133687, Asia n= 100698, Europe n = 204582, North America n = 5029090, Oceania n = 9797, South America n = 15582).

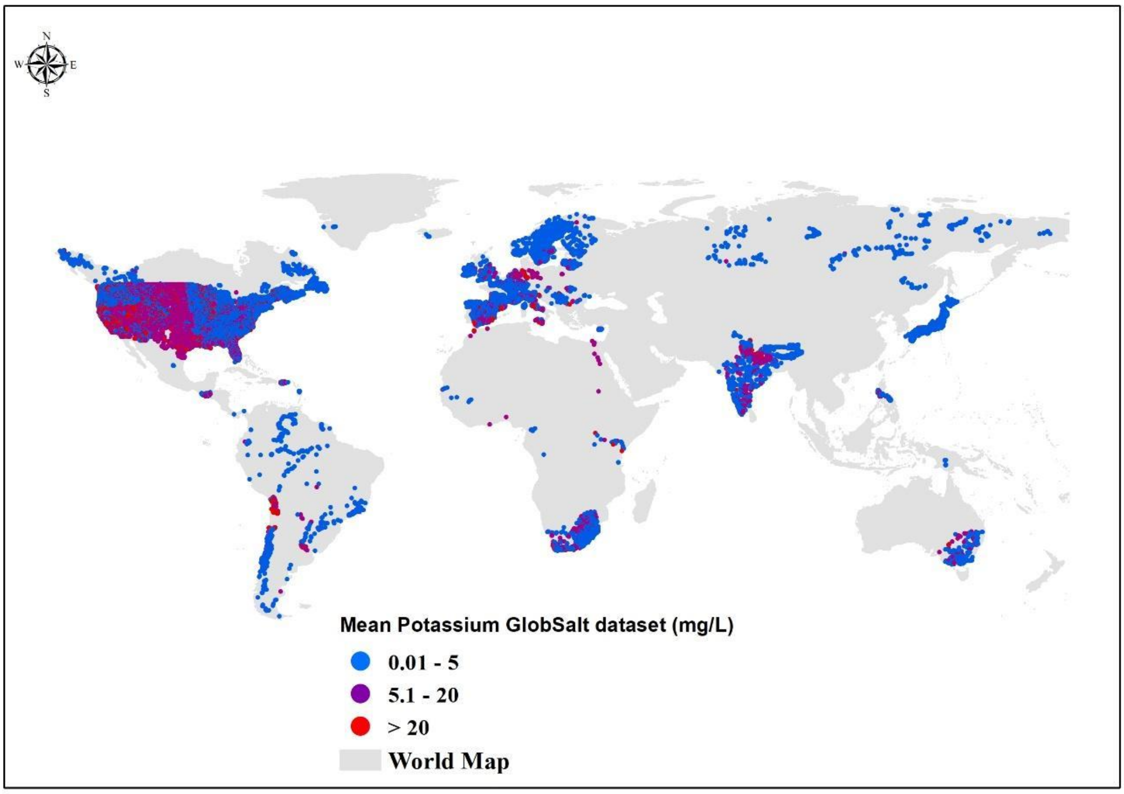

Figure C7. Global distribution of measured mean dissolved potassium and station density. The global map in the panel shows the measured mean potassium values per river in each country, based on observations included in the GlobSalt database, over the entire data period (1980-2023). Blue, purple, and red dots represent low, moderate, and high potassium levels, respectively.

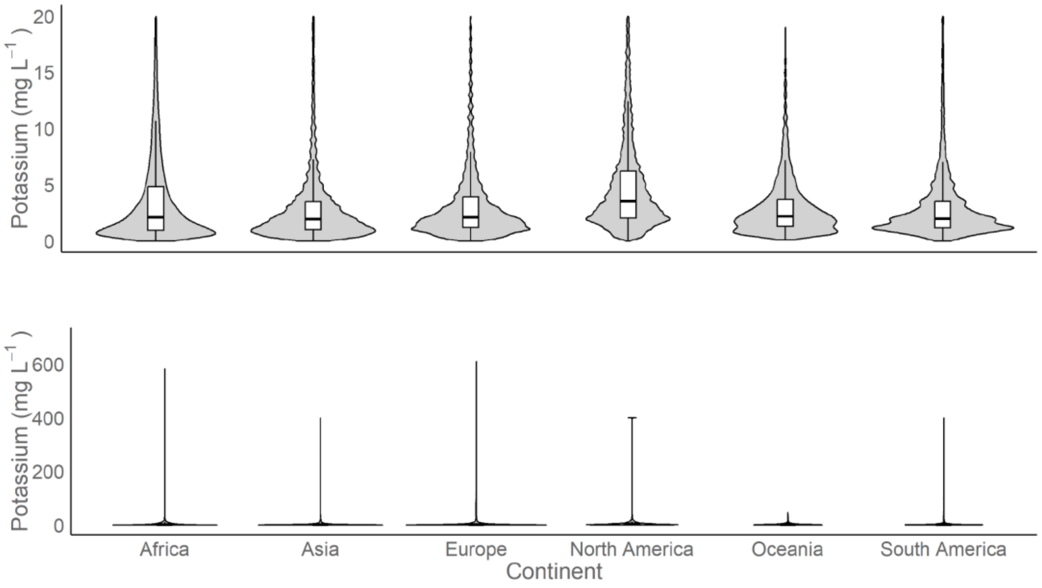

Figure C8. Global violin plot of dissolved potassium by continent. Top: Global Violin Plot of potassium by Continent with Zoom to 20 mg L^{-1}. Bottom: Global Violin Plot of potassium across the entire scale by Continent. Potassium observations are included in the GlobSalt database, over the entire data period (1980-2023). Each gray violin plot represents the available data set according to the number (n) of observations per continent (Africa n = 130020, Asia n = 100855, Europe n = 154423, North America n = 4794390, Oceania n = 9706, South America n = 16667).

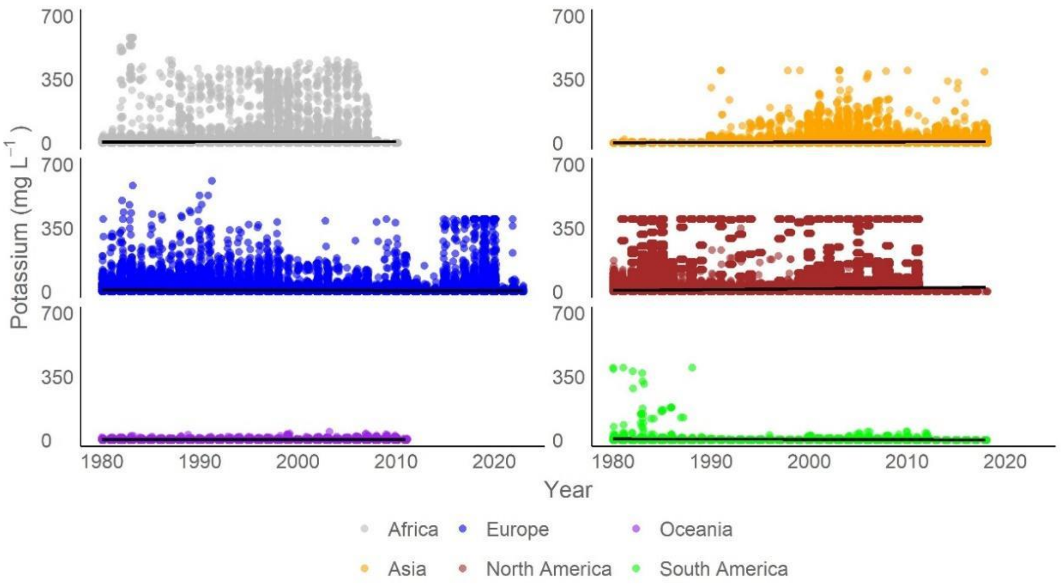

Figure C9. Global temporal dispersion plot of dissolved potassium by continent. Potassium observations are included in the GlobSalt database over the entire data period (1980-2023). Each color point (gray, yellow, blue, brown, purple, and green) represents the number (n) of observations per continent (Africa n = 130020, Asia n = 100855, Europe n = 154423, North America n = 4794390, Oceania n = 9706, South America n = 16667).

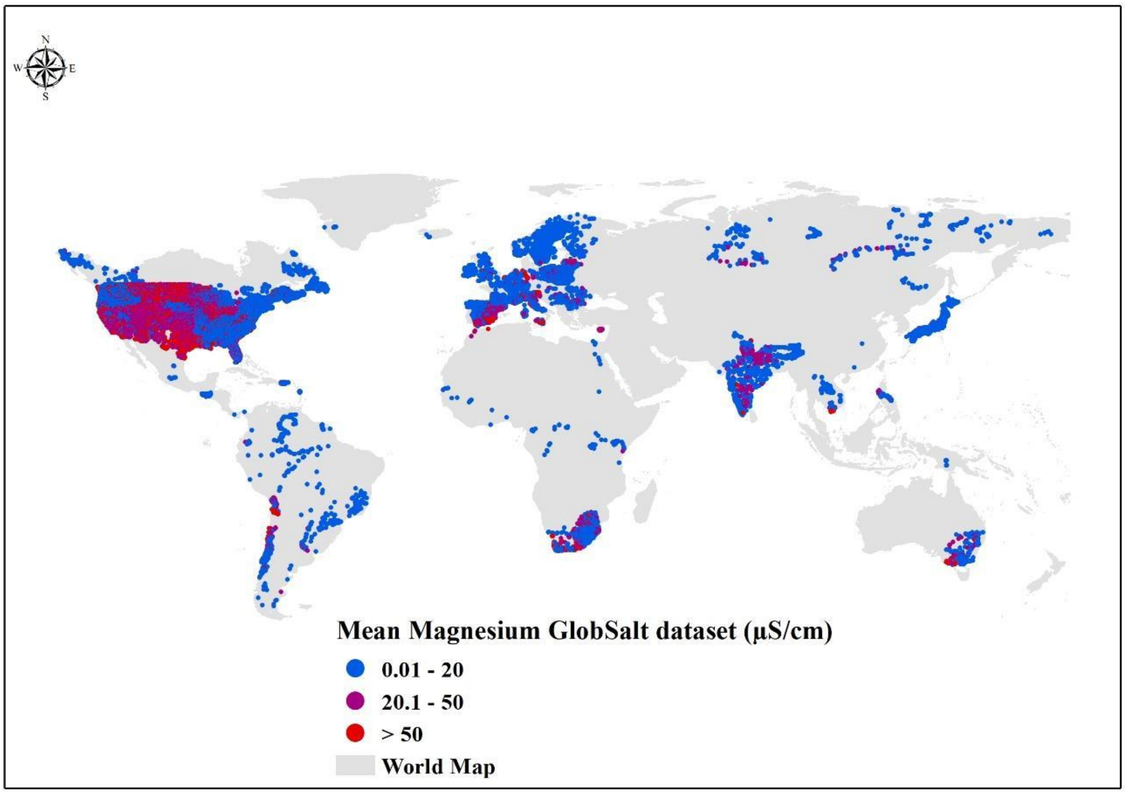

Figure C10. Global distribution of measured mean dissolved magnesium and station density. The global map in the panel shows the measured mean magnesium values per river in each country, based on observations included in the GlobSalt database, over the entire data period (1980-2023). Blue, purple, and red dots represent low, moderate, and high magnesium levels, respectively.

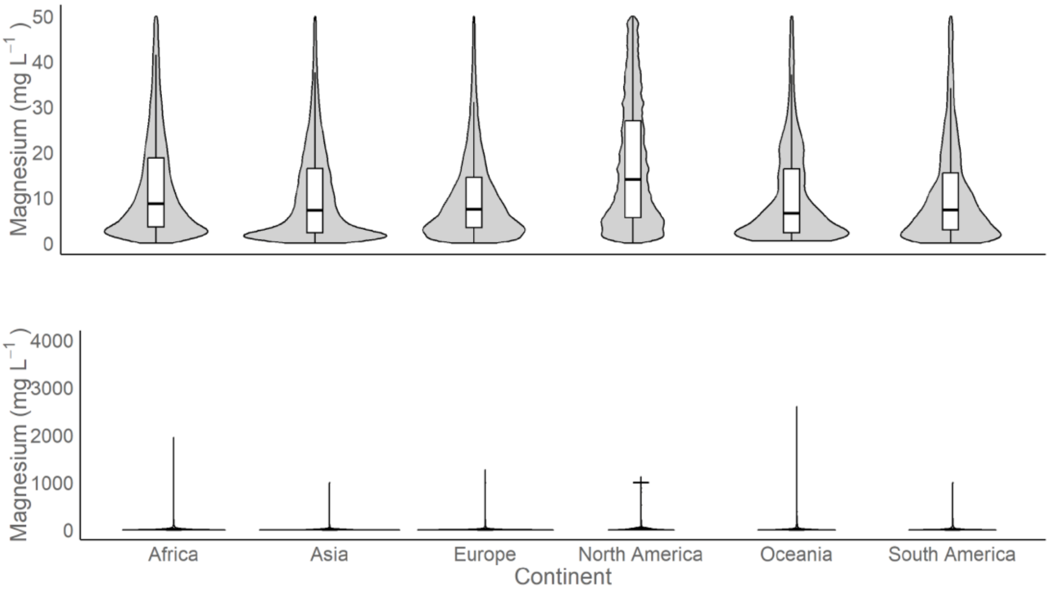

Figure C11. Global violin plot of dissolved magnesium by continent. Top: Global Violin Plot of magnesium by Continent with Zoom to 50 mg L^{-1}. Bottom: Global Violin Plot of magnesium across the entire scale by Continent. Magnesium observations are included in the GlobSalt database, over the entire data period (1980-2023). Each gray violin plot represents the available data set according to the number (n) of observations per continent (Africa n = 133816, Asia n = 111565, Europe n = 193759, North America n = 5489267, Oceania n = 9807, South America n = 15678).

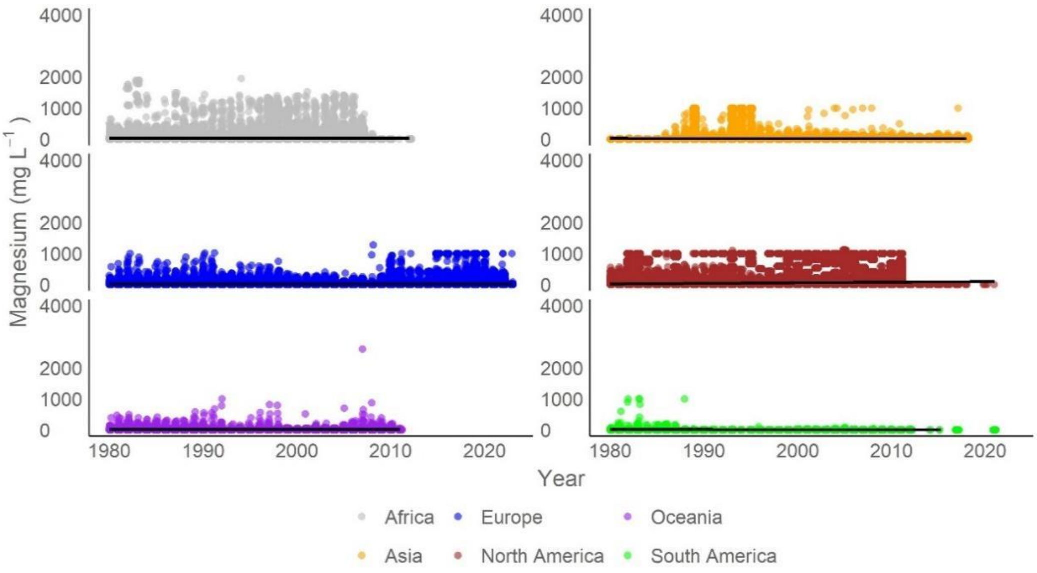

Figure C12. Global temporal dispersion plot of dissolved magnesium by continent. Magnesium observations are included in the GlobSalt database over the entire data period (1980-2023). Each color point (gray, yellow, blue, brown, purple, and green) represents the number (n) of observations per continent (Africa n = 133816, Asia n = 111565, Europe n = 193759, North America n = 5489267, Oceania n = 9807, South America n = 15678).

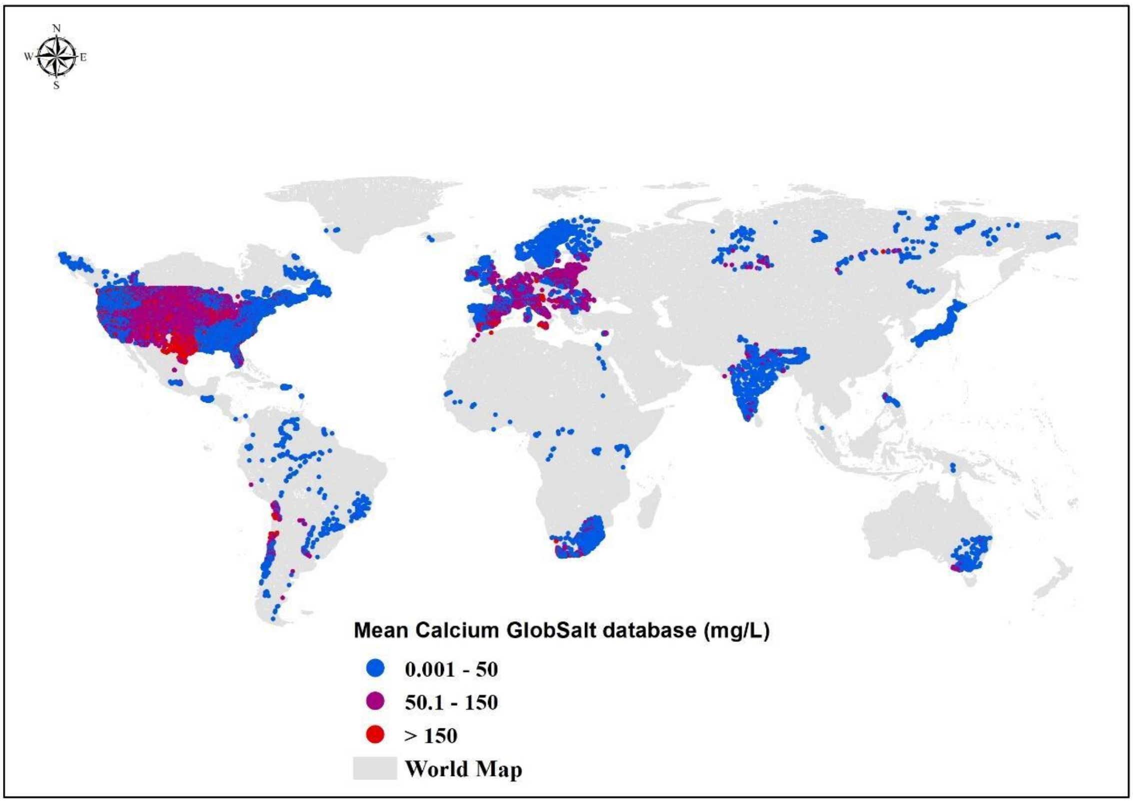

Figure C13. Global distribution of measured mean dissolved calcium and station density. The global map in the panel shows the measured mean calcium values per river in each country, based on observations included in the GlobSalt database, over the entire data period (1980-2023). Blue, purple, and red dots represent low, moderate, and high calcium levels, respectively.

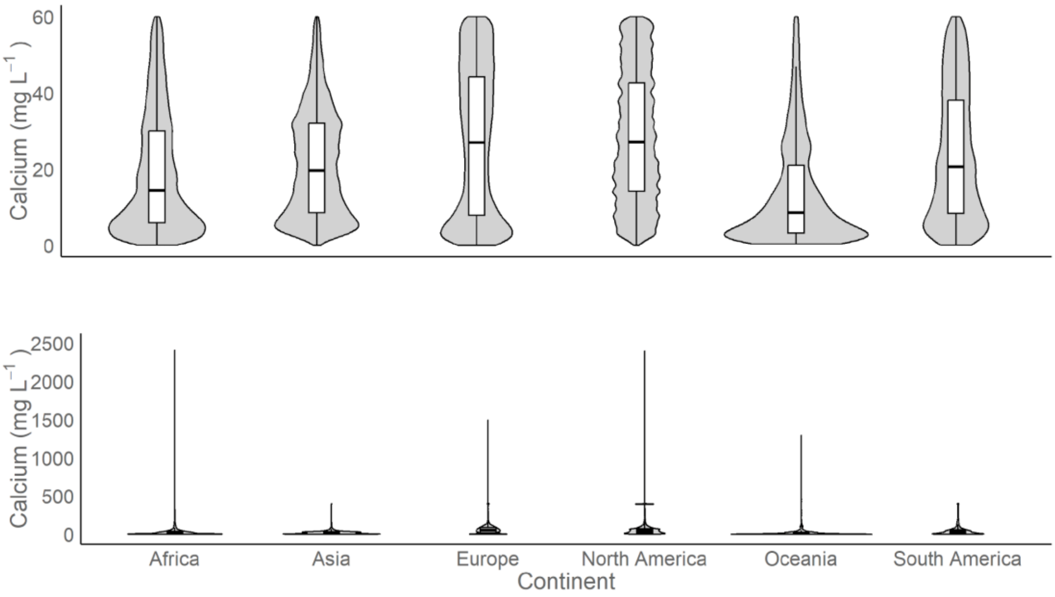

Figure C14. Global violin plot of dissolved calcium by continent. Top: Global Violin Plot of calcium by Continent with Zoom to 60 mg L^{-1}. Bottom: Global Violin Plot of calcium across the entire scale by Continent. Calcium observations are included in the GlobSalt database, over the entire data period (1980-2023). Each gray violin plot represents the available data set according to the number (n) of observations per continent (Africa n = 133361, Asia n = 105402, Europe n = 262310, North America n = 5559902, Oceania n = 9463, South America n = 15644).

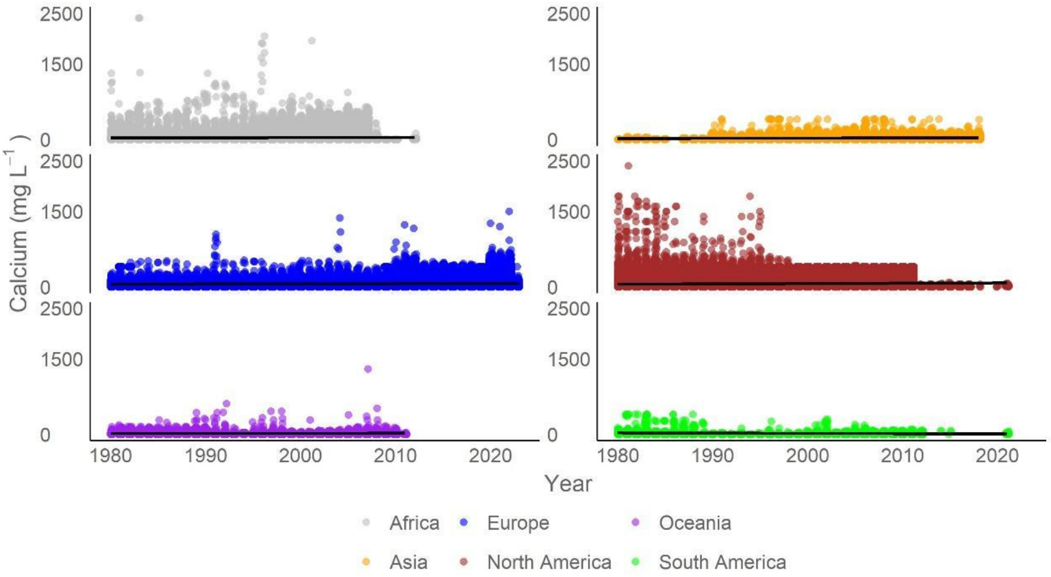

Figure C15. Global temporal dispersion plot of dissolved calcium by continent. Calcium observations are included in the GlobSalt database over the entire data period (1980-2023). Each color point (gray, yellow, blue, brown, purple, and green) represents the number (n) of observations per continent (Africa n = 133361, Asia n = 105402, Europe n = 262310, North America n = 5559902, Oceania n = 9463, South America n = 15644).

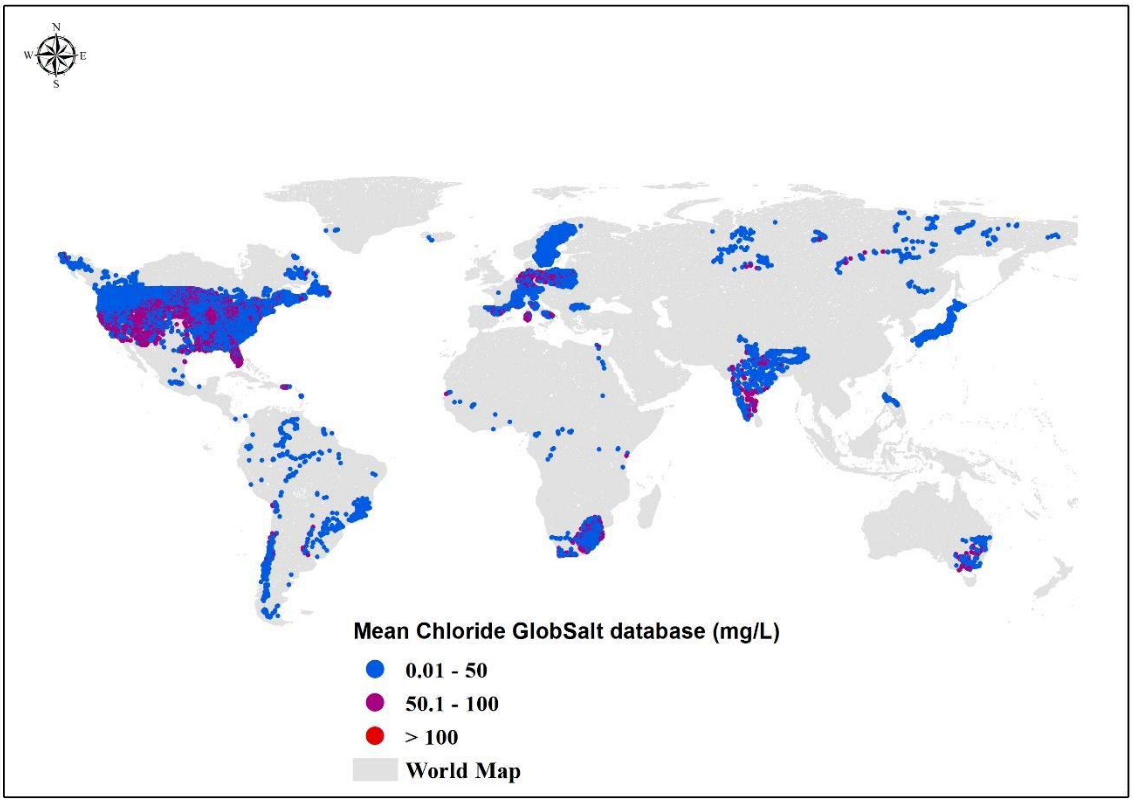

Figure C16. Global distribution of measured mean dissolved chloride and station density. The global map in the panel shows the measured mean chloride values per river in each country, based on observations included in the GlobSalt database, over the entire data period (1980-2023). Blue, purple, and red dots represent low, moderate, and high chloride levels, respectively.

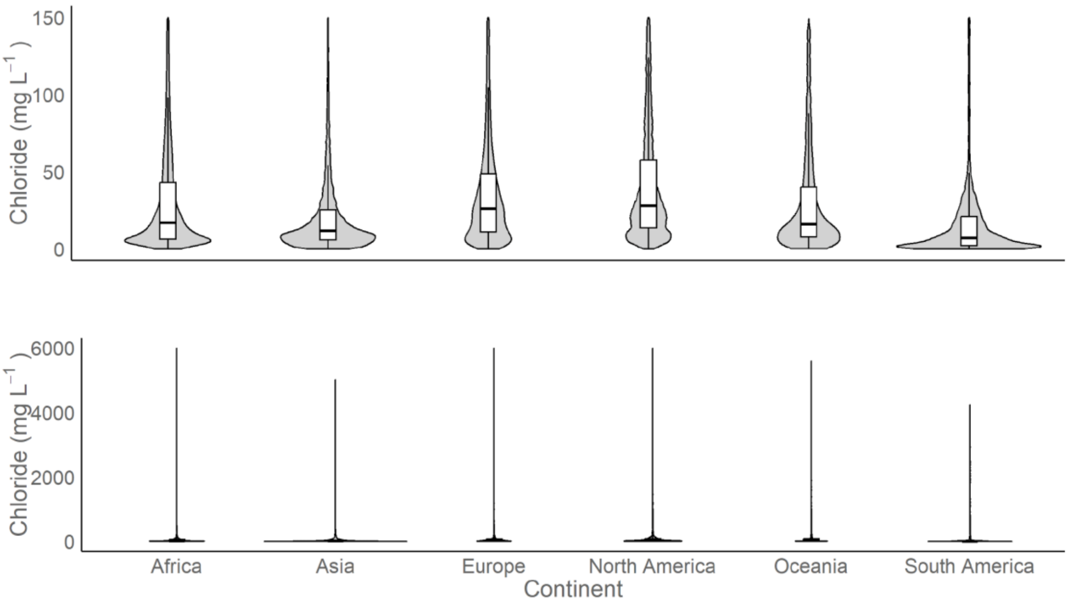

Figure C17. Global violin plot of dissolved chloride by continent. Top: Global Violin Plot of chloride by Continent with Zoom to 150 mg L^{-1}. Bottom: Global Violin Plot of chloride across the entire scale by Continent. Chloride observations are included in the GlobSalt database, over the entire data period (1980-2023). Each gray violin plot represents the available data set according to the number (n) of observations per continent (Africa n = 135515, Asia n = 106452, Europe n = 175926, North America n = 5373355, Oceania n = 9488, South America n = 17769).

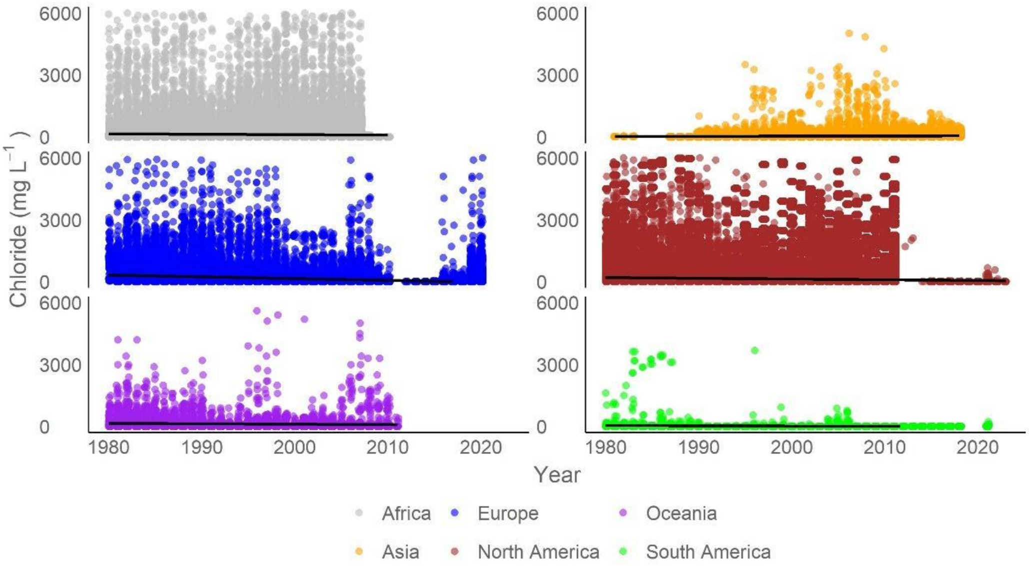

Figure C18. Global temporal dispersion plot of dissolved chloride by continent. Chloride observations are included in the GlobSalt database over the entire data period (1980-2023). Each color point (gray, yellow, blue, brown, purple, and green) represents the number (n) of observations per continent (Africa n = 135515, Asia n = 106452, Europe n = 175926, North America n = 5373355, Oceania n = 9488, South America n = 17769).

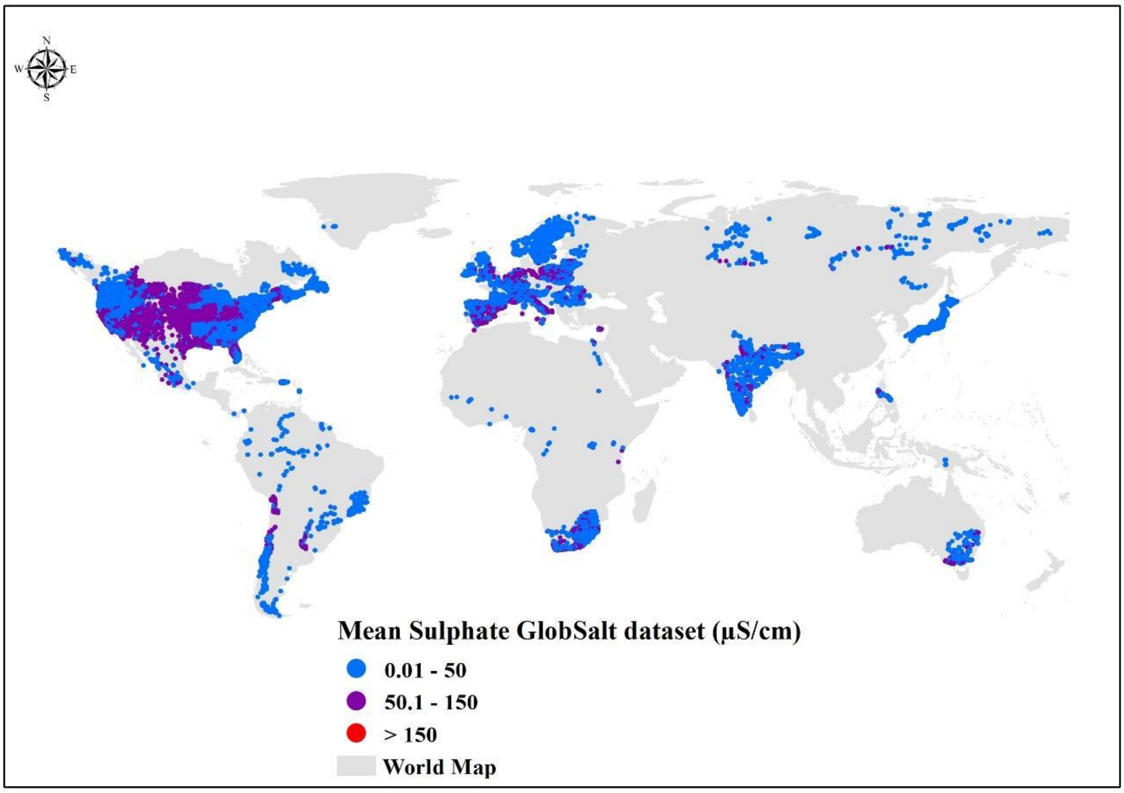

Figure C19. Global distribution of measured mean sulphate and station density. The global map in the panel shows the measured mean sulphate values per river in each country, based on observations included in the GlobSalt database, over the entire data period (1980-2023). Blue, purple, and red dots represent low, moderate, and high sulphate levels, respectively.

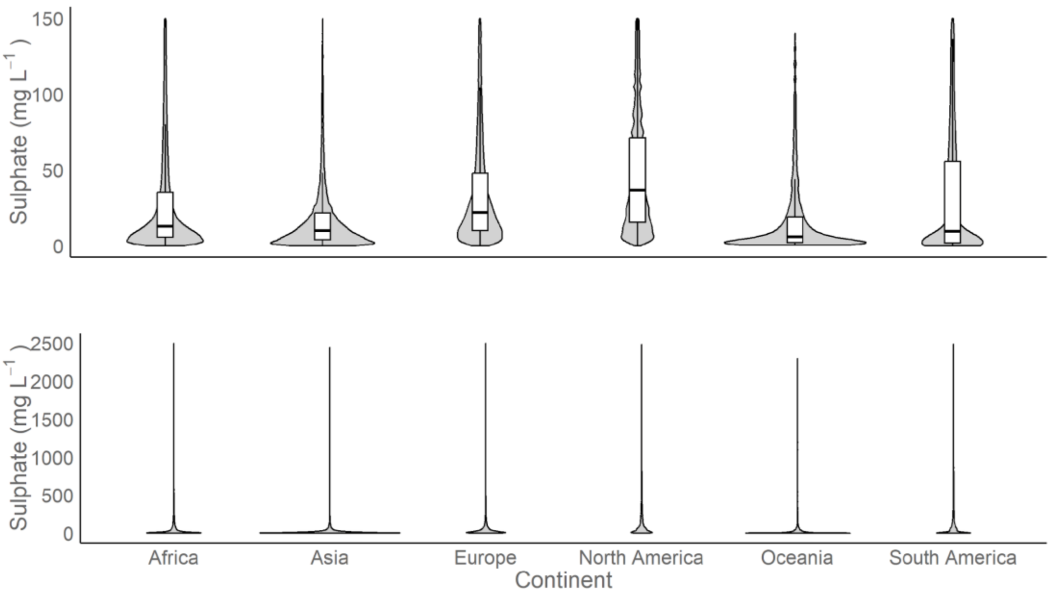

Figure C20. Global violin plot of dissolved sulphate by continent. Top: Global Violin Plot of sulphate by Continent with Zoom to 150 mg L^{-1}. Bottom: Global Violin Plot of sulphate across entire scale by Continent. Sulphate observations are included in the GlobSalt database, over the entire data period (1980-2023). Each gray violin plot represents the available data set according to the number (n) of observations per continent (Africa n = 136929, Asia n = 97109, Europe n = 239298, North America n = 4803213, Oceania n = 8153, South America n = 17114).

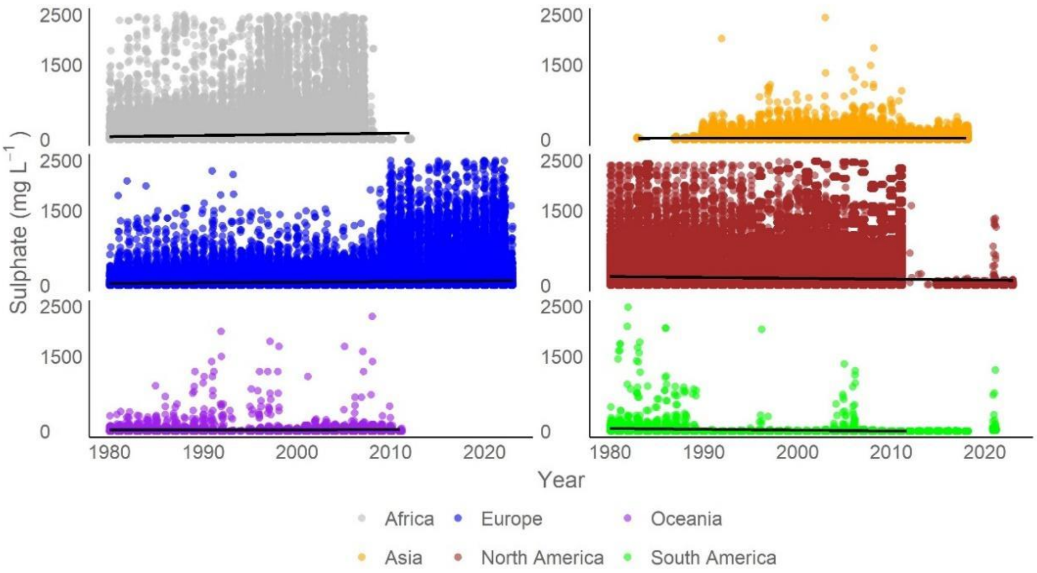

Figure C21. Global temporal dispersion plot of dissolved sulphate by continent. Sulphate observations are included in the GlobSalt database over the entire data period (1980-2023). Each color point (gray, yellow, blue, brown, purple, and green) represents the number (n) of observations per continent (Africa n = 136929, Asia n = 97109, Europe n = 394531, North America n = 4803213, Oceania n = 8153, South America n = 17114).

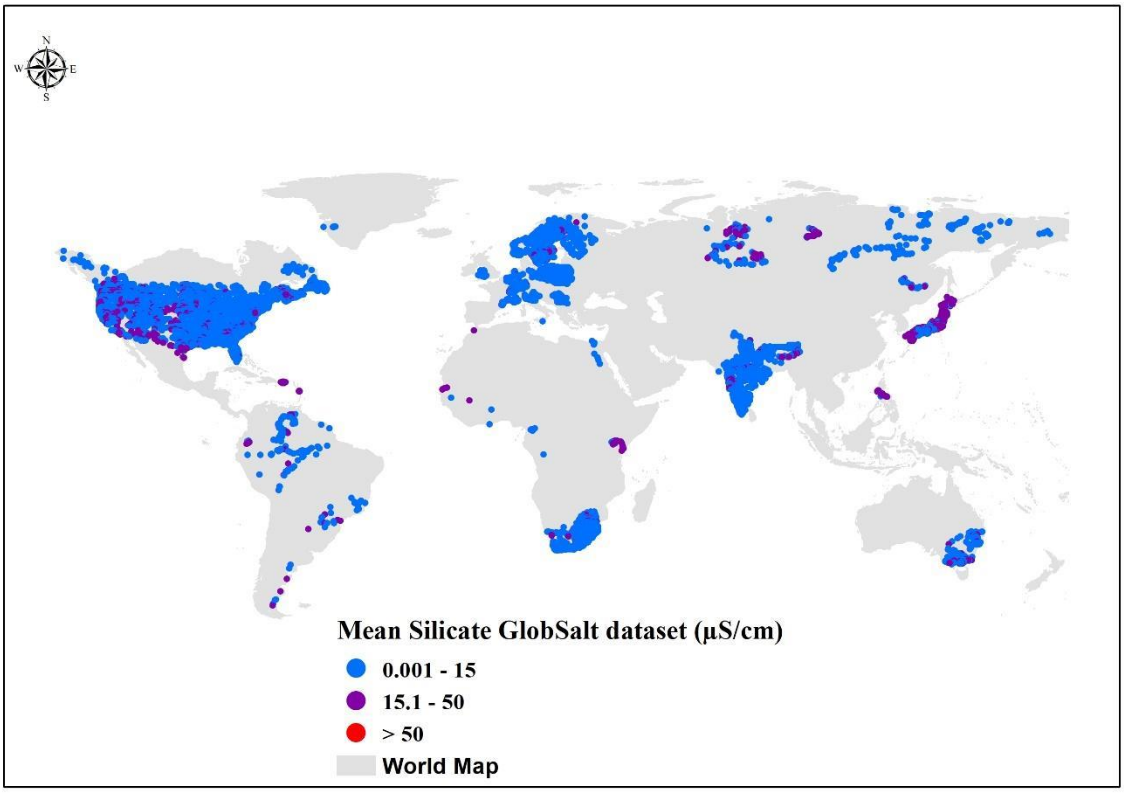

Figure C22. Global distribution of measured mean dissolved silicate and station density. The global map in the panel shows the measured mean silicate values per river in each country, based on observations included in the GlobSalt database, over the entire data period (1980-2023). Blue, purple, and red dots represent low, moderate, and high silicate levels, respectively.

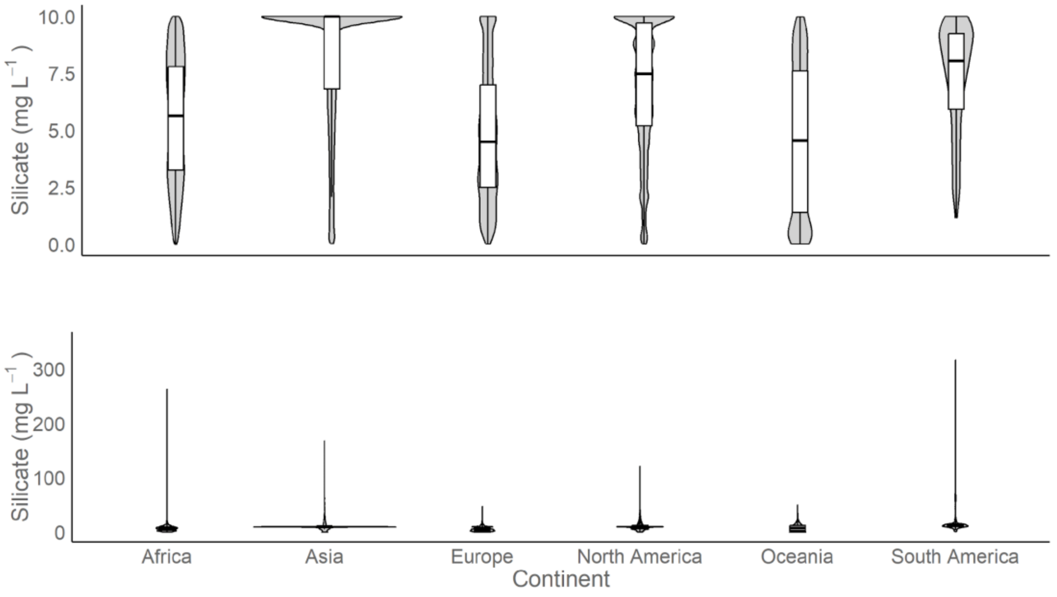

Figure C23. Global violin plot of dissolved silicate by continent. Top: Global Violin Plot of silicate by Continent with Zoom to 10 mg L^{-1}. Bottom: Global Violin Plot of silicate across the entire scale by Continent. Silicate observations are included in the GlobSalt database, over the entire data period (1980-2023). Each gray violin plot represents the available data set according to the number (n) of observations per continent (Africa n = 125842, Asia n = 79112, Europe n = 34883, North America n = 175049, Oceania n = 11055, South America n = 1992).

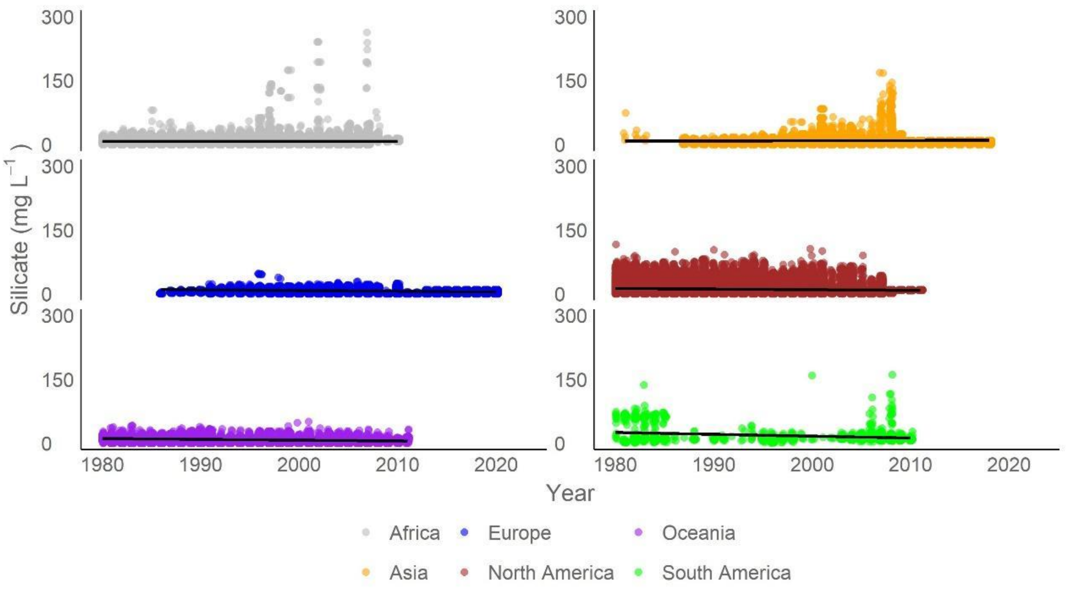

Figure C24. Global temporal dispersion plot of dissolved silicate by continent. Silicate observations are included in the GlobSalt database over the entire data period (1980-2023). Each color point (gray, yellow, blue, brown, purple, and green) represents the number (n) of observations per continent (Africa n = 125842, Asia n = 79112, Europe n = 34883, North America n = 175049, Oceania n = 11055, South America n = 1992).

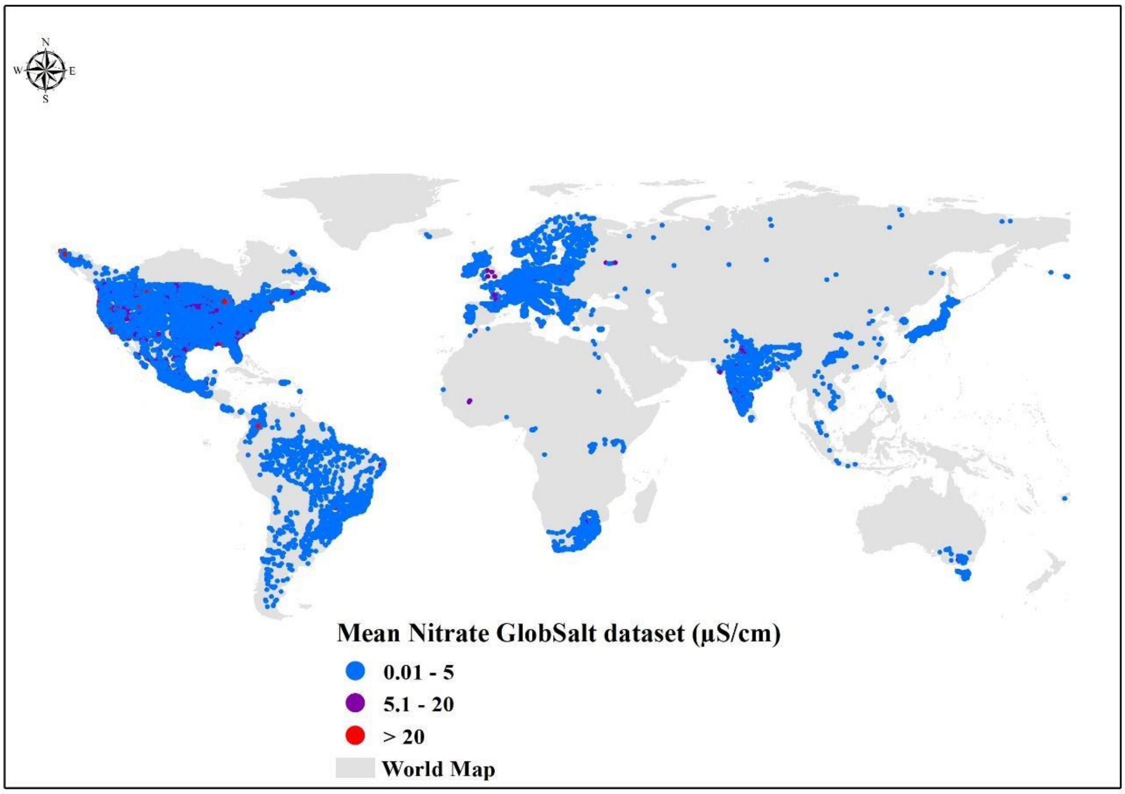

Figure C25. Global distribution of measured mean nitrate and station density. The global map in the panel shows the measured mean nitrate values per river in each country, based on observations included in the GlobSalt database, over the entire data period (1980-2023). Blue, purple, and red dots represent low, moderate, and high nitrate levels, respectively.

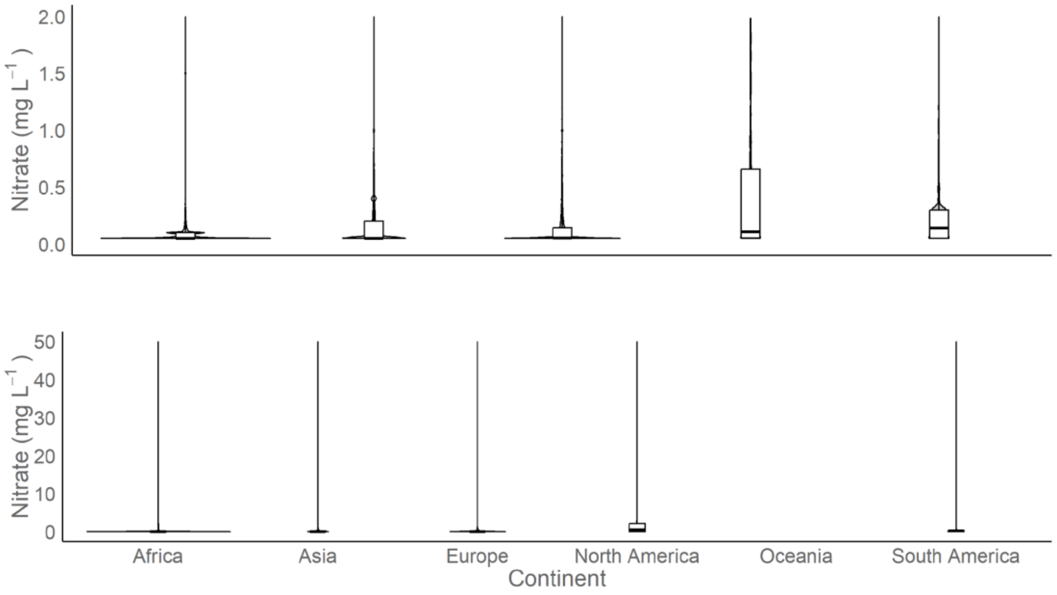

Figure C26. Global violin plot of nitrate by continent. Top: Global Violin Plot of nitrate by Continent with Zoom to 20 mg L^{-1}. Bottom: Global Violin Plot of nitrate across the entire scale by Continent. Nitrate observations are included in the GlobSalt database, over the entire data period (1980-2023). Each gray violin plot represents the available data set according to the number (n) of observations per continent (Africa n = 69429, Asia n = 68447, Europe n = 478303, North America n = 1147316, Oceania n = 6180, South America n = 25707).

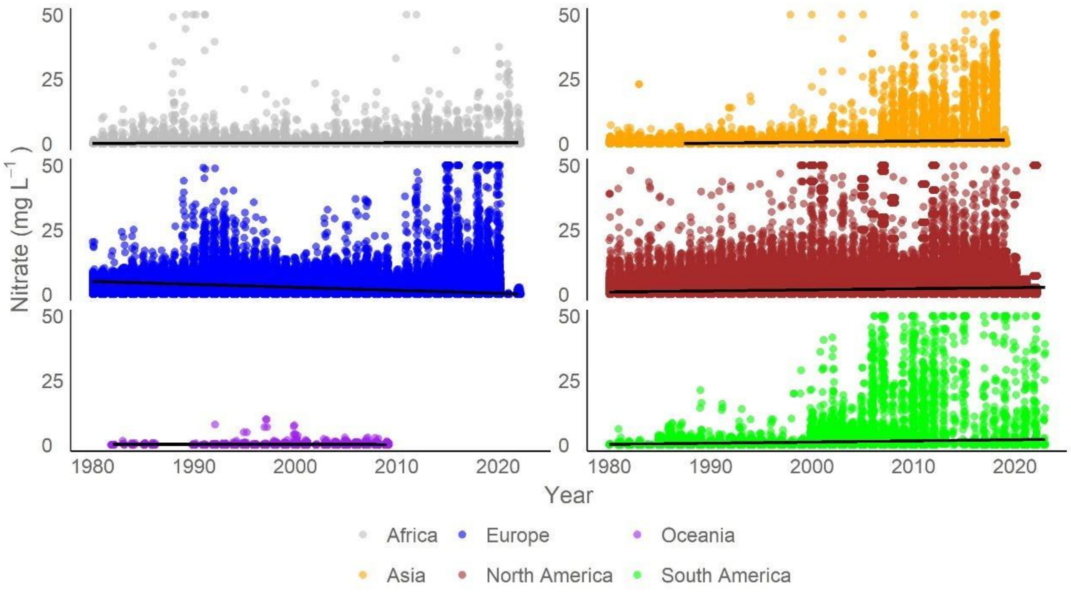

Figure C27. Global temporal dispersion plot of nitrate by continent. Nitrate observations are included in the GlobSalt database over the entire data period (1980-2023). Each color point (gray, yellow, blue, brown, purple, and green) represents the number (n) of observations per continent (Africa n = 69429, Asia n = 68447, Europe n = 478303, North America n = 1147316, Oceania n = 6180, South America n = 25707).

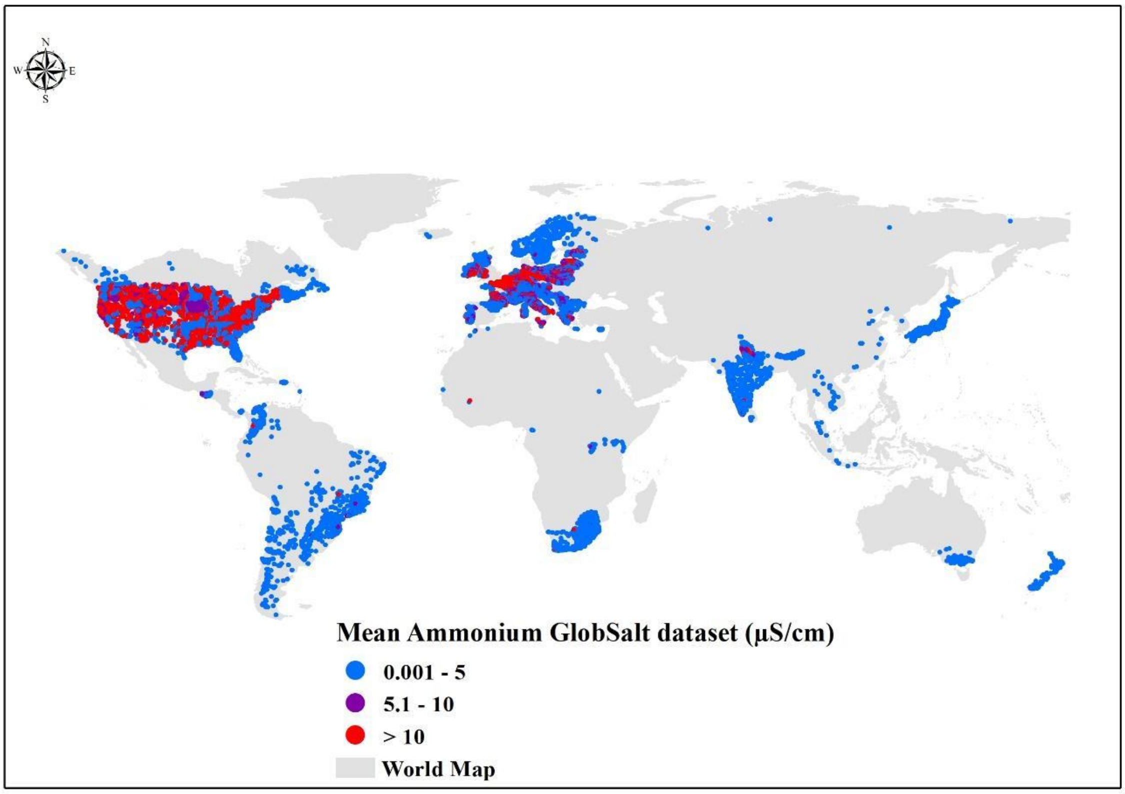

Figure C28. Global distribution of measured mean ammonium and station density. The global map in the panel shows the measured mean ammonium values per river in each country, based on observations included in the GlobSalt database, over the entire data period (1980-2023). Blue, purple, and red dots represent low, moderate, and high ammonium levels, respectively.

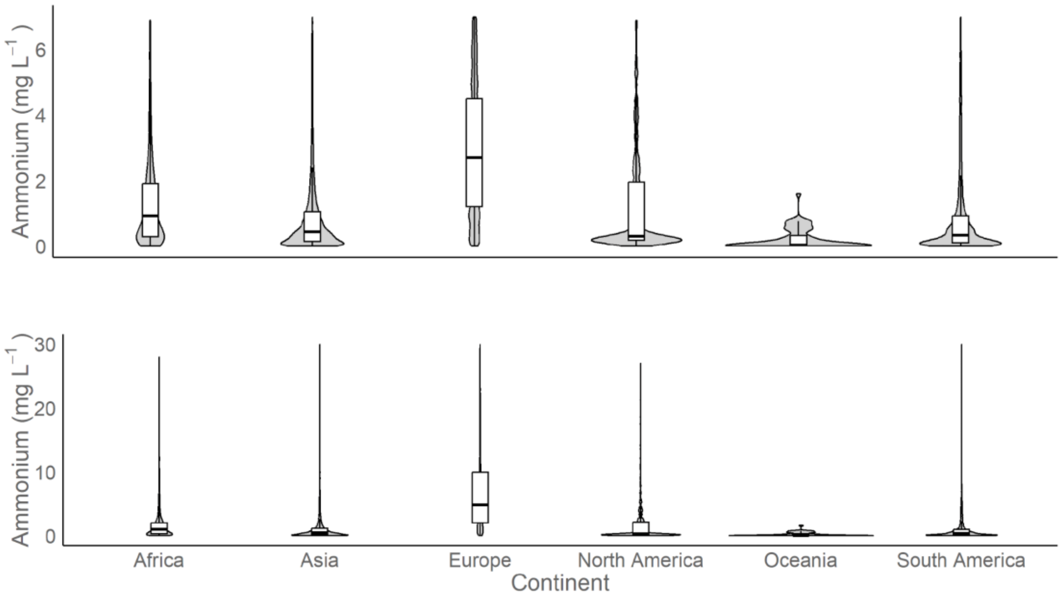

Figure C29. Global violin plot of ammonium by continent. Top: Global Violin Plot of ammonium by Continent with Zoom to 6 mg L^{-1}. Bottom: Global Violin Plot of ammonium across the entire scale by Continent. Ammonium observations are included in the GlobSalt database, over the entire data period (1980-2023). Each gray violin plot represents the available data set according to the number (n) of observations per continent (Africa n = 140913, Asia n = 66527, Europe n = 747967, North America n = 371981, Oceania n = 3541, South America n = 27876).

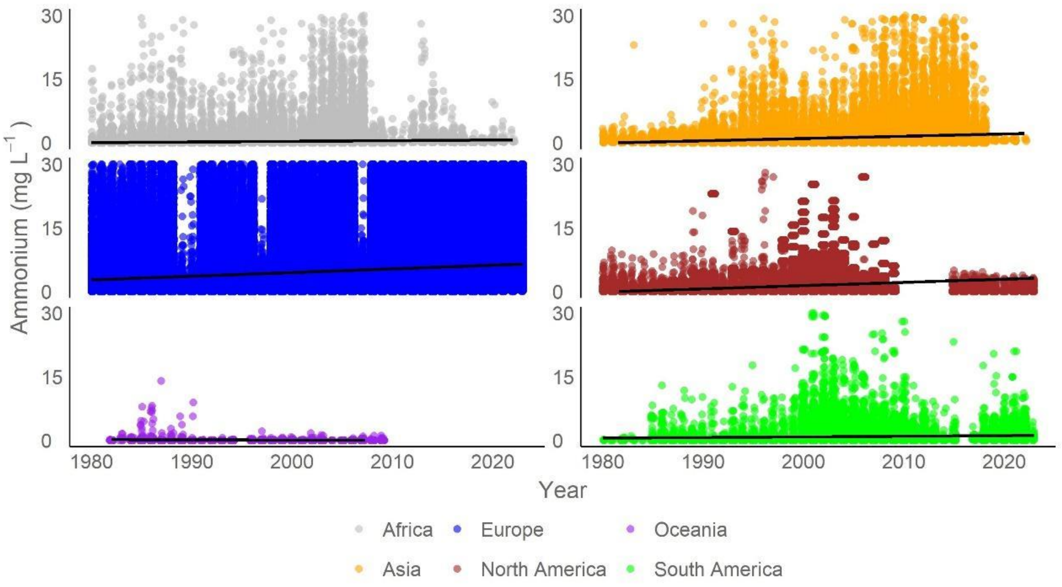

Figure C30. Global temporal dispersion plot of ammonium by continent. Ammonium observations are included in the GlobSalt database over the entire data period (1980-2023). Each color point (gray, yellow, blue, brown, purple, and green) represents the number (n) of observations per continent (Africa n = 140913, Asia n = 66527, Europe n = 747967, North America n = 371981, Oceania n = 3541, South America n = 27876).

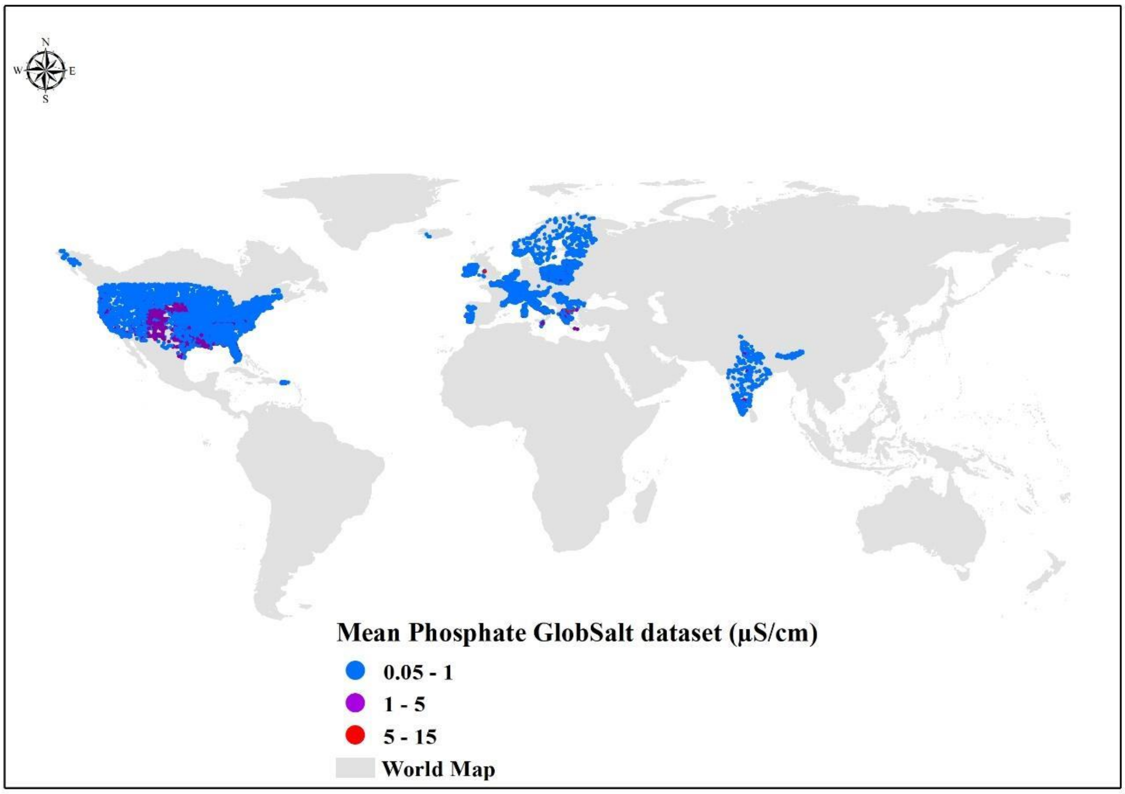

Figure C31. Global distribution of measured mean phosphate and station density. The global map in the panel shows the measured mean phosphate values per river in each country, based on observations included in the GlobSalt database, over the entire data period (1980-2023). Blue, purple, and red dots represent low, moderate, and high phosphate levels, respectively.

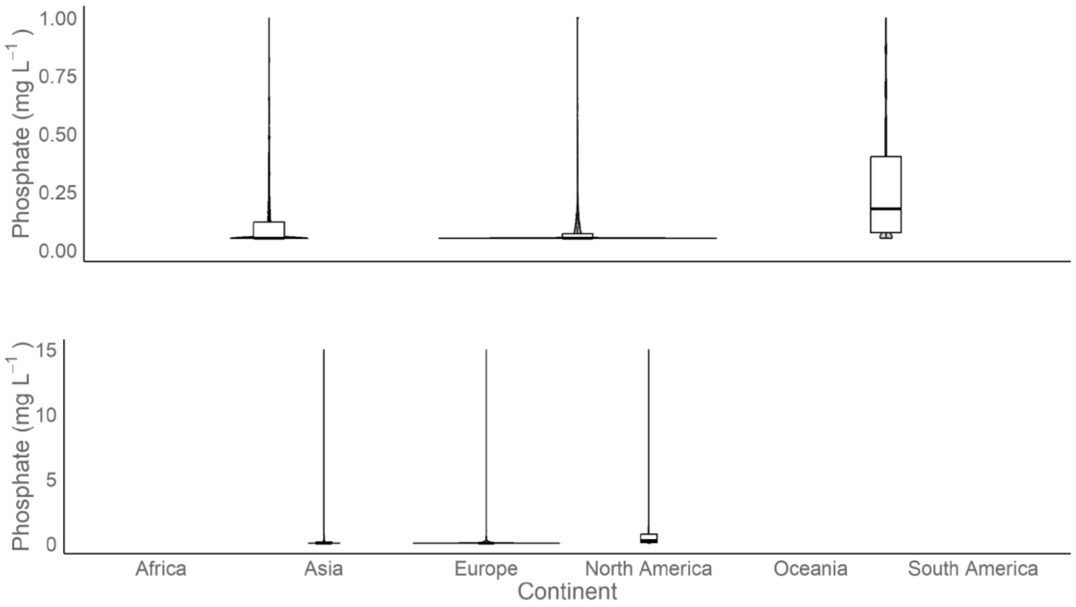

Figure C32. Global violin plot of phosphate by continent. Top: Global Violin Plot of phosphate by Continent with Zoom to 20 mg L-1. Bottom: Global Violin Plot of phosphate across entire scale by Continent. Phosphate observations are included in the GlobSalt database, over the entire data period (1980-2023). Each gray violin plot represents the available data set according to the number (n) of observations per continent (Asia n = 33641, Europe n = 174630, North America n = 748642).

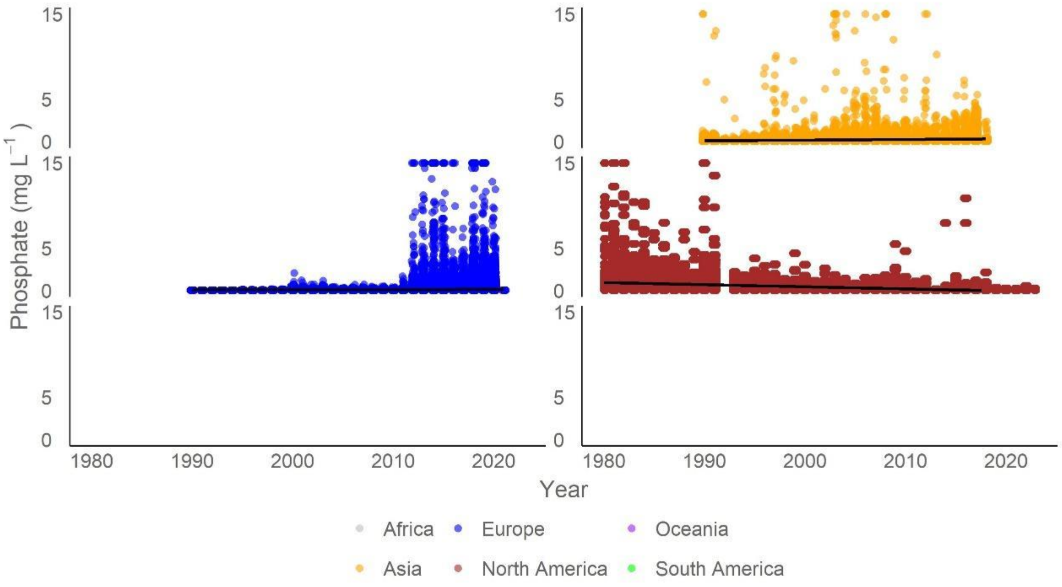

Figure C33. Global temporal dispersion plot of phosphate by continent. Phosphate observations are included in the GlobSalt database over the entire data period (1980-2023). Each color point (gray, yellow, blue, brown, purple, and green) represents the number (n) of observations per continent (Asia n = 33641, Europe n = 174630, North America n = 748642).

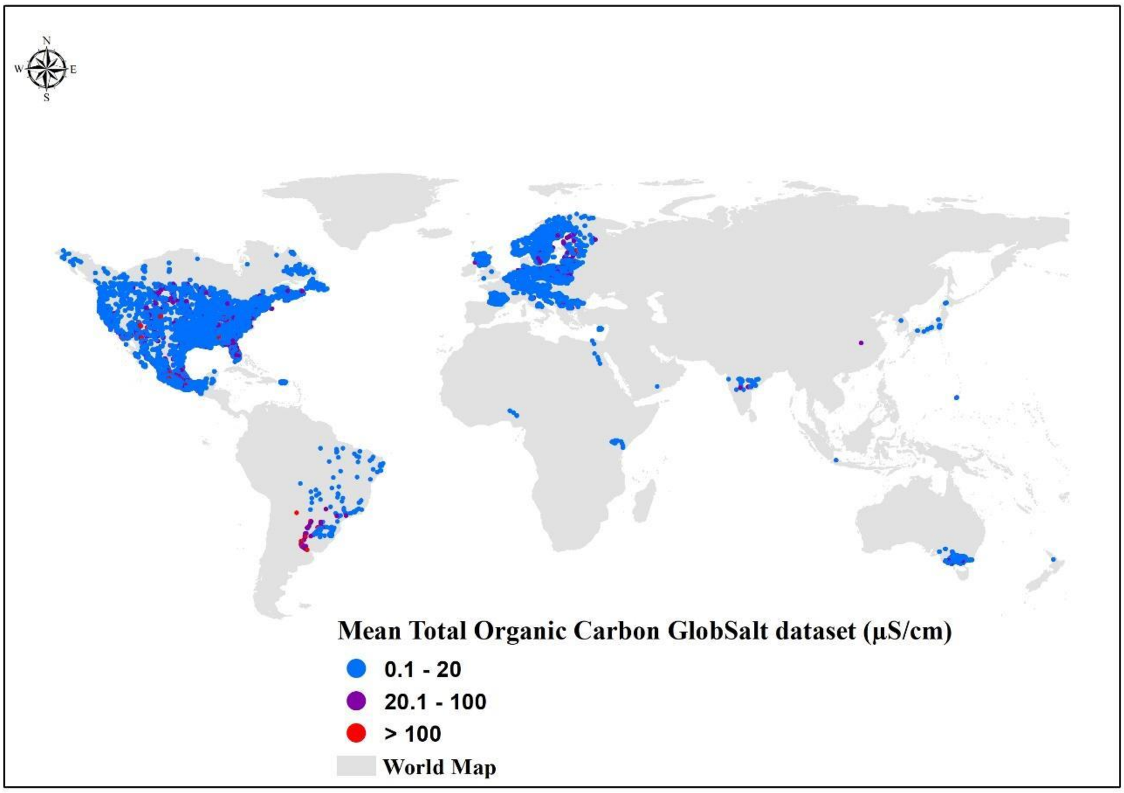

Figure C34. Global distribution of measured mean total organic carbon (TOC) and station density. The global map in the panel shows the measured mean TOC values per river in each country, based on observations included in the GlobSalt database, over the entire data period (1980-2023). Blue, purple, and red dots represent low, moderate, and high TOC levels, respectively.

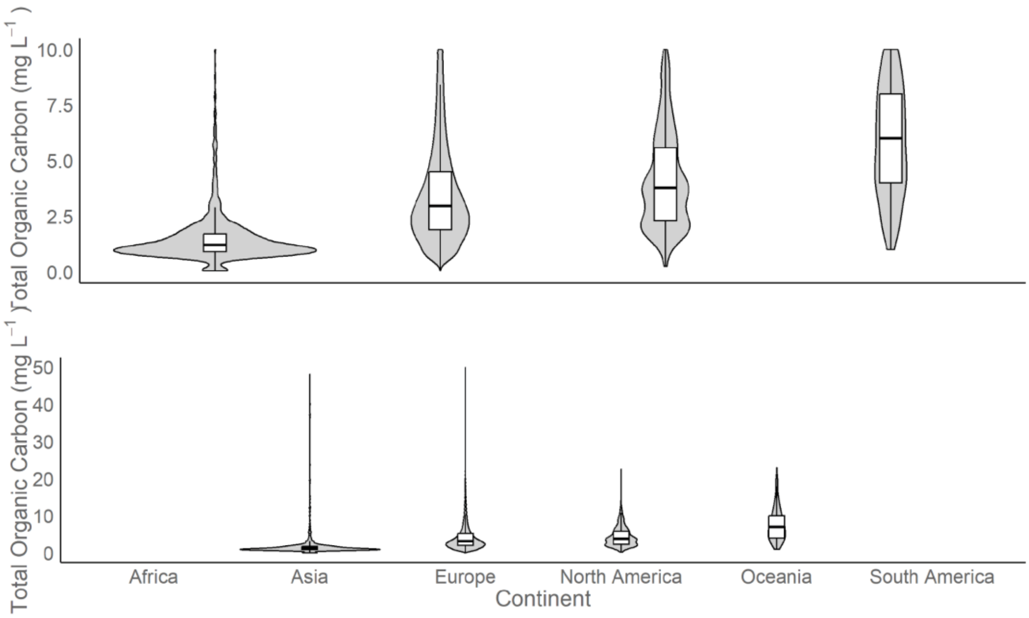

Figure C35. Global violin plot of total organic carbon (TOC) by continent. Top: Global Violin Plot of total organic carbon (TOC) by Continent with Zoom to 20 mg L-1. Bottom: Global Violin Plot of total organic carbon (TOC) across the entire scale by Continent. Total organic carbon (TOC) observations are included in the GlobSalt database, over the entire data period (1980-2023). Each gray violin plot represents the available data set according to the number (n) of observations per continent (Africa n = 63, Asia n = 3804, Europe n = 499640, North America n = 313367, Oceania n = 4010, South America n = 310).

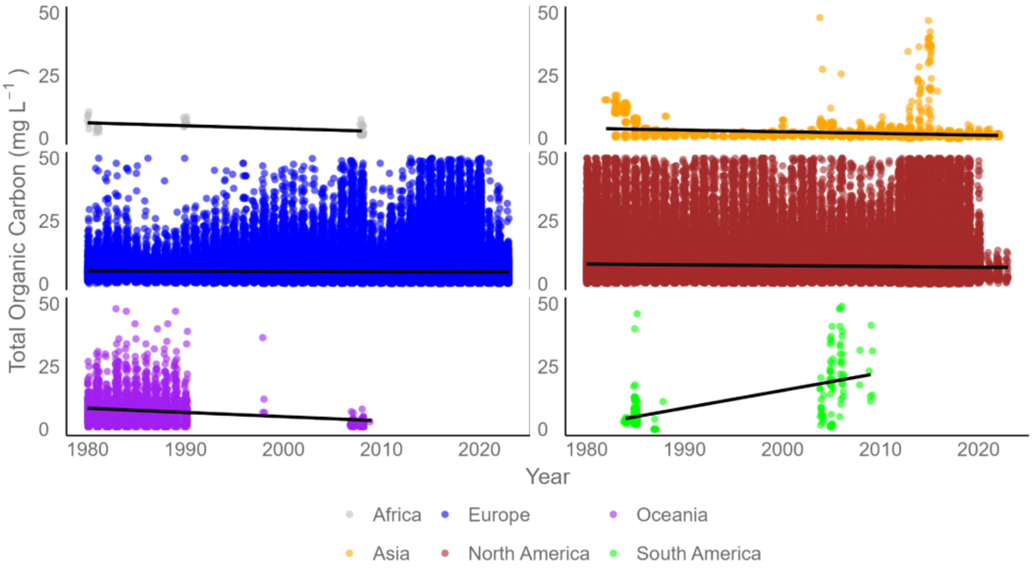

Figure C36. Global temporal dispersion plot of total organic carbon (TOC) by continent. Total organic carbon (TOC) observations are included in the GlobSalt database over the entire data period (1980-2023). Each color point (gray, yellow, blue, brown, purple, and green) represents the number (n) of observations per continent (Africa n = 63, Asia n = 3804, Europe n = 499640, North America n = 313367, Oceania n = 4010, South America n = 310).

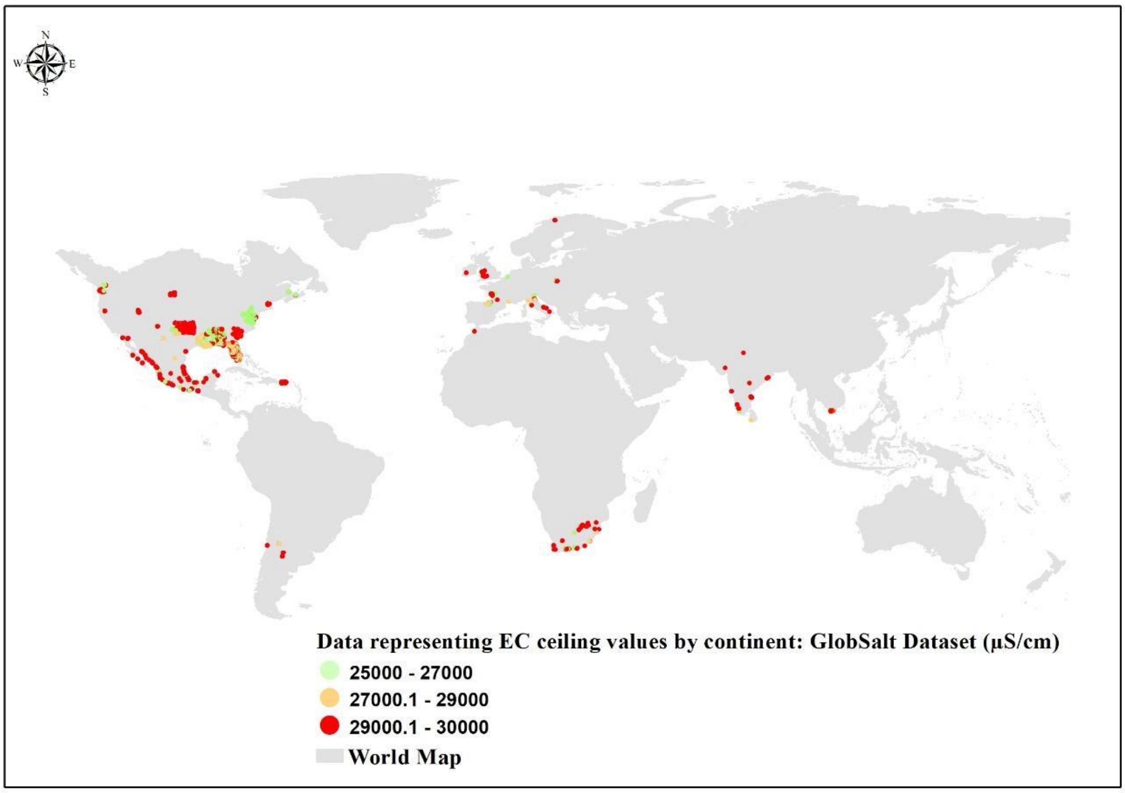

Figure C37. Ceiling values of electrical conductivity by continent. The map illustrates the ceiling EC values that have been consistently observed across multiple stations within each continent, based on data from the GlobSalt database spanning the entire period (1980-2023). Green, orange, and red dots indicate low, moderate, and high ceiling values, respectively, providing insights into the presence of consistently ceiling EC levels across continents (Africa n = 426, Asia n = 328, Europe n = 934, North America n = 19081, South America n = 574).

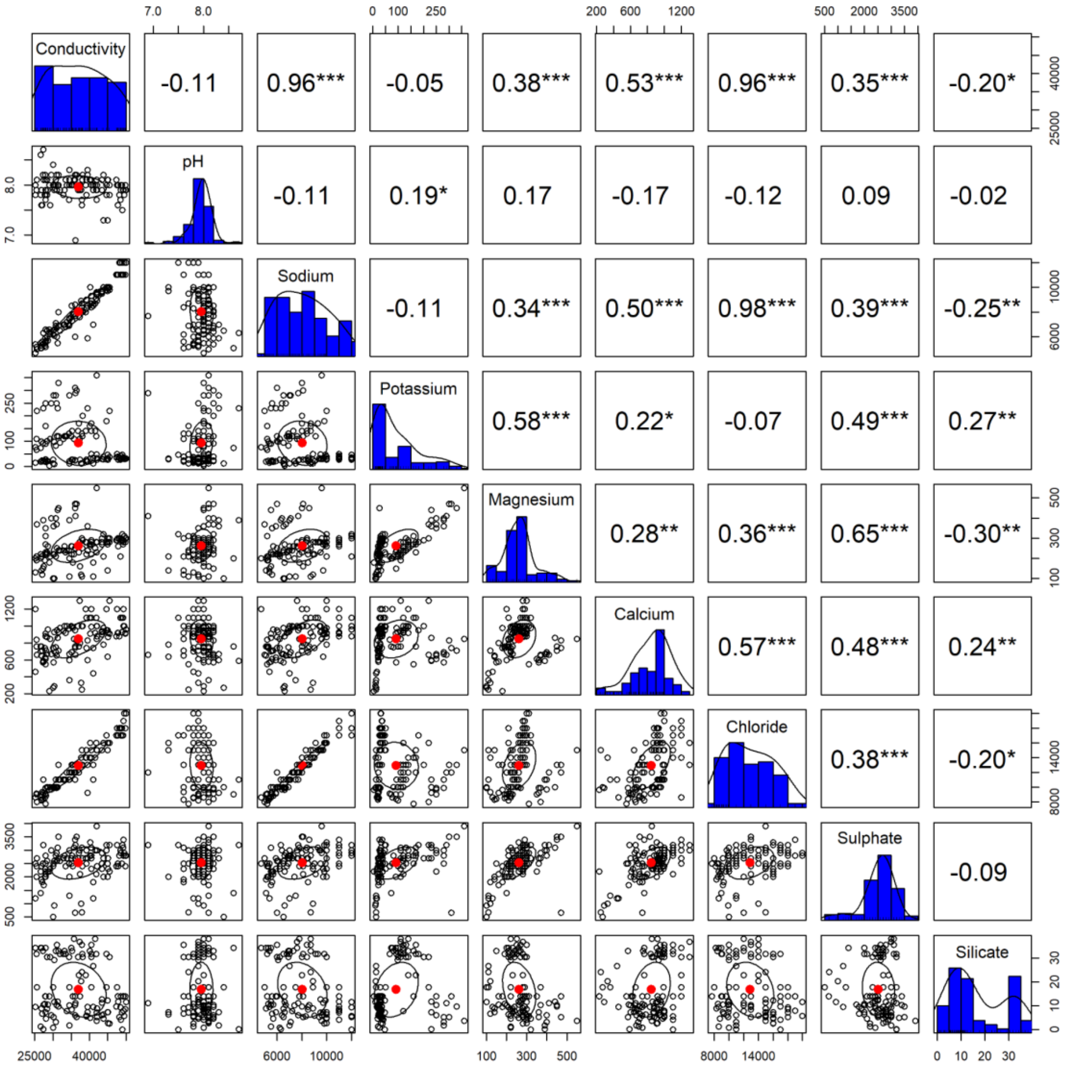

Figure C38. Correlation plot of electrical conductivity (EC) ceiling values and ion concentrations in the GlobSalt dataset, including inter-ion correlations. Red dots indicate the strength of correlation between variables (stronger intensity represents higher correlation, while weaker intensity represents lower correlation).

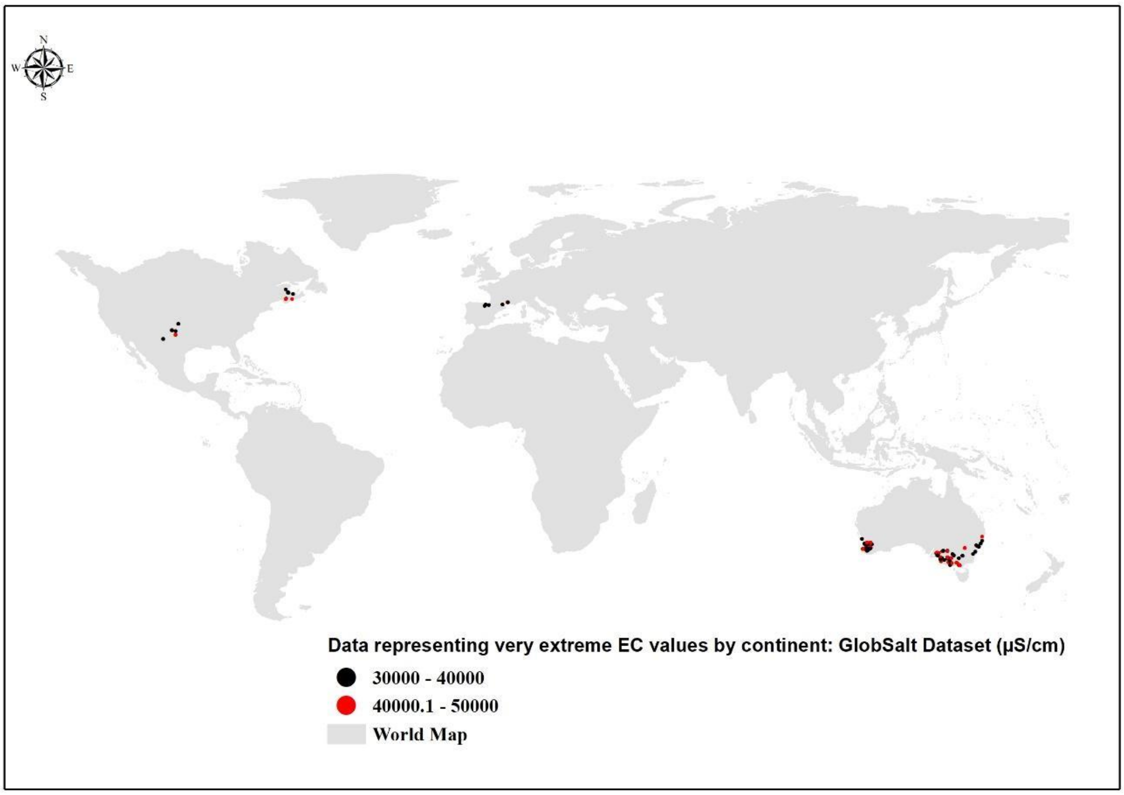

Figure C39. Extreme values of electrical conductivity by continent. The map illustrates the highest and moderately high EC values measured per continent, based on observations included in the GlobSalt database, over the entire data period (1980-2023). Black dots indicate very high EC values, while red dots indicate the highest EC values. Both black and red dots represent extreme EC values, providing insights into the range of EC across continents (Europe n = 39, North America n = 160, South America n = 1895).

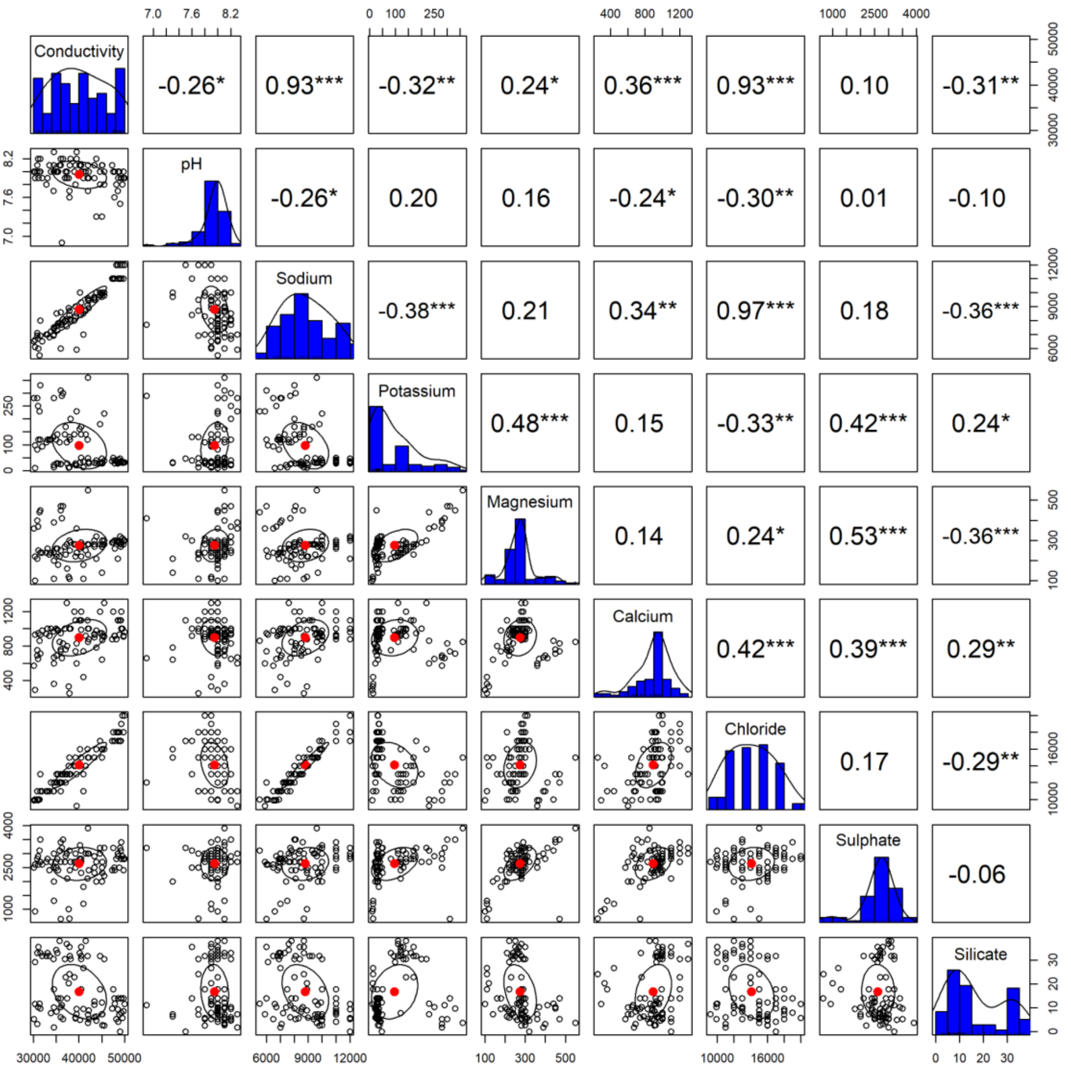

Figure C40. Correlation plot of electrical conductivity (EC) extreme values and ion concentrations in the GlobSalt dataset, including inter-ion correlations. Red dots indicate the strength of correlation between variables (stronger intensity represents higher correlation, while weaker intensity represents lower correlation).

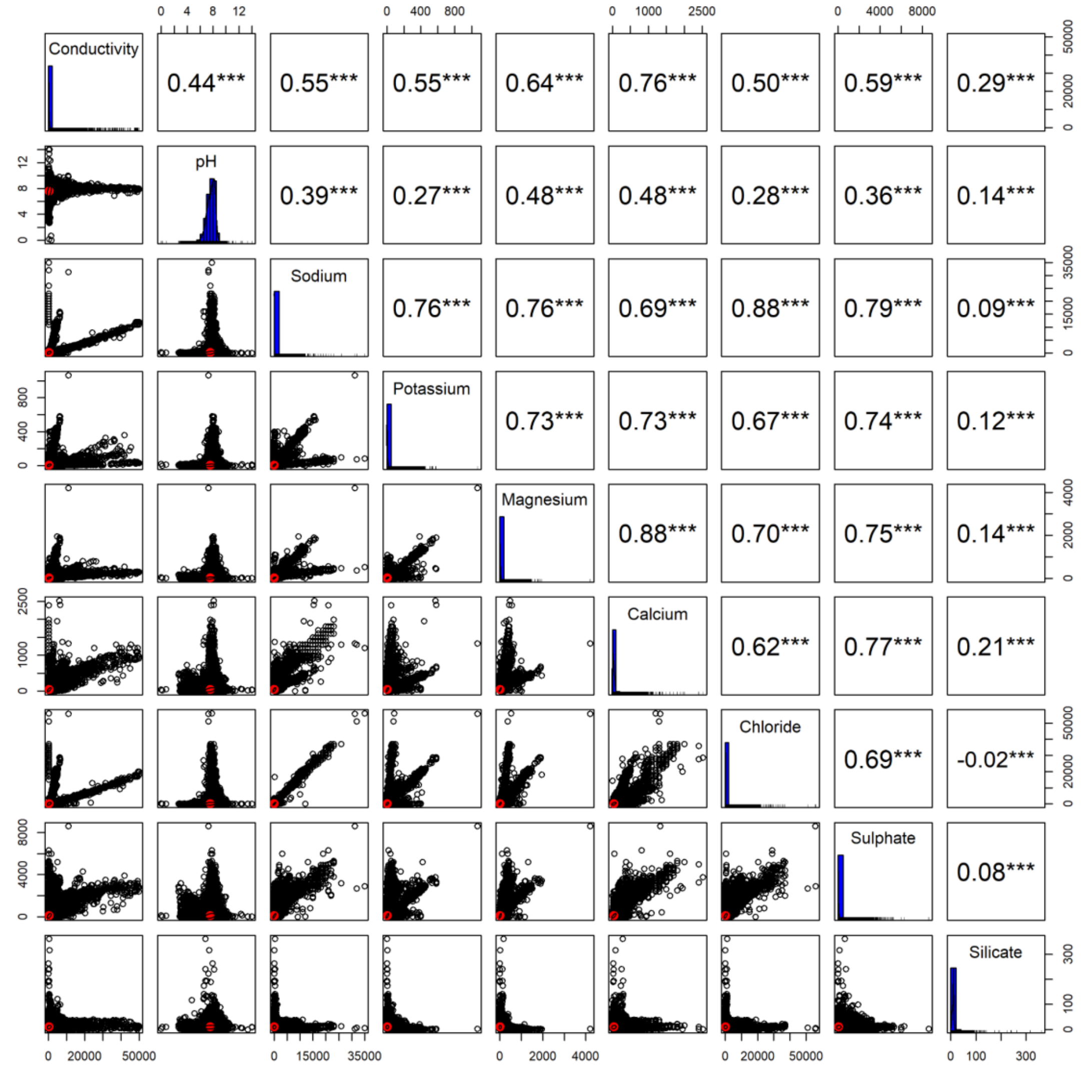

Figure C41. Correlation plot of electrical conductivity (EC) and ion concentrations in the GlobSalt dataset (unfiltered potential data errors), including inter-ion correlations (ions unit, mg L^{-1}). Red dots indicate the strength of correlation between variables. The location and intensity of the red dots can provide further insights into the relationship between the variables (e.g., stronger intensity of red color represents higher correlation, while weaker intensity suggests lower correlation).
